# Risk factors for dementia and cognitive impairment within 5 years after stroke: a prospective multicentre cohort study

**DOI:** 10.1016/j.lanepe.2025.101428

**Published:** 2025-08-19

**Authors:** Jule Filler, Marios K. Georgakis, Daniel Janowitz, Marco Duering, Rong Fang, Anna Dewenter, Felix J. Bode, Sebastian Stoesser, Christine Kindler, Peter Hermann, Christian H. Nolte, Thomas G. Liman, Lucia Kerti, Kathleen Bernkopf, Benno Ikenberg, Wenzel Glanz, Michael Wagner, Annika Spottke, Karin Waegemann, Michael Goertler, Silke Wunderlich, Matthias Endres, Inga Zerr, Gabor C. Petzold, Martin Dichgans, DEMDAS Investigators, Tatjana Wittenberg, Jan F. Scheitz, Harald Prüss, Pia Sophie Sperber, Alexander H. Nave, Anna Kufner Ibaroule, Julius N. Meißner, Taraneh Ebrahimi, Julia Nordsiek, Niklas Beckonert, Matthias Schmitz, Stefan Goebel, Timothy Bunck, Julia Schütte-Schmidt, Sabine Nuhn, Corinna Volpers, Peter Dechent, Matthias Bähr, Anna Kopczak, Frank Wollenweber, Christiane Huber, Holger Poppert, Tony Stöcker, Katja Neumann, Oliver Speck

**Affiliations:** a Institute for Stroke and Dementia Research (ISD), LMU University Hospital, LMU Munich, Munich, Germany; b Graduate School for Systemic Neurosciences, Ludwig-Maximilians-University, Munich, Germany; c Program in Medical and Population Genetics, Broad Institute of MIT and Harvard, Cambridge, MA, USA; d Munich Cluster for Systems Neurology (SyNergy), Munich, Germany; e Medical Image Analysis Center (MIAC AG) and Department of Biomedical Engineering, University of Basel, Basel, Switzerland; f German Center for Neurodegenerative Diseases (DZNE), Bonn, Germany; g Department of Vascular Neurology, University Hospital Bonn, Bonn, Germany; h Department of Neurology, University Medical Center Göttingen, Göttingen, Germany; i German Center for Neurodegenerative Diseases (DZNE, Berlin), Berlin, Germany; j Department of Neurology with Experimental Neurology, Charité - Univeristätsmedizin Berlin, Berlin, Germany; k Berlin Institute of Health (BIH), Berlin, Germany; l Center for Stroke Research Berlin (CSB), Charité – Universitätsmedizin Berlin, Berlin, Germany; m German Centre for Cardiovascular Research (DZHK), Partner Site Berlin, Berlin, Germany; n Department of Neurology, Carl Von Ossietzky University, Oldenburg, Germany; o Department of Neurology, TUM School of Medicine, Technical University of Munich, Munich, Germany; p Department of Neurology, University Hospital, Otto-von-Guericke University Magdeburg, Magdeburg, Germany; q German Center for Neurodegenerative Diseases (DZNE), Magdeburg, Germany; r Department of Old Age Psychiatry and Cognitive Disorders, University Hospital Bonn, Bonn, Germany; s German Center for Neurodegenerative Diseases (DZNE, Munich), Munich, Germany; t German Center for Mental Health (DZPG), Partner Site Berlin, Berlin, Germany; u Klinik und Hochschulambulanz für Neurologie, Charité – Universitätsmedizin Berlin, Berlin, Germany; v German Center for Neurodegenerative Diseases (DZNE), Göttingen, Germany; w German Centre for Cardiovascular Research (DZHK, Munich), Munich, Germany

**Keywords:** Stroke, Brain ischaemia, Risk factors, Stroke outcomes, Stroke epidemiology, Dementia, Vascular dementia, Dementia epidemiology, Post-stroke dementia, Post-stroke cognitive impairment, Cognitive decline, Diabetes, Metabolic syndrome, Small vessel disease

## Abstract

**Background:**

Stroke survivors frequently experience subsequent cognitive impairment or dementia. We aimed to identify risk factors for post-stroke dementia (PSD) and cognitive impairment (PSCI) within 5 years after stroke.

**Methods:**

The DEMDAS (German Center for Neurological Diseases (DZNE) mechanisms of dementia after stroke) study is a prospective cohort of stroke patients admitted to six German tertiary stroke centres between May 1, 2011 and January 31, 2019. Eligible dementia-free patients with ischaemic or haemorrhagic stroke underwent baseline examinations and regular clinical, neuropsychological, and neuroimaging follow-ups over 5 years, with the last follow-ups completed in January 2024. PSD was the primary outcome, determined by comprehensive cognitive testing, patient and informant interviews, and review of medical records. The secondary outcomes were early-onset PSD (3–6 months), delayed-onset PSD (>6 months), and PSCI. Associations between baseline risk factors and PSD were assessed using Cox regression models adjusted for age, sex, education, and stroke severity.

**Findings:**

Of 736 patients (245 [33%] female, mean age 68·0 years [SD 11·2], median admission National Institutes of Health Stroke Scale (NIHSS) 3 [IQR 1–5]), 557 (76%) were followed up until death or the end of the study, and 706 (96%) contributed to the PSD analysis. During a median of 5·0 years [IQR 3·3–5·1] of follow-up, 55 new dementia cases were diagnosed (6-month incidence: 3·1% [1·8–4·5], 5-year incidence: 8·8% [6·5–11·1]), of which 21 (38%) were classified as early-onset PSD. The 5-year risk of PSD was associated with older age (HR 1·13 [95% CI 1·08–1·18] per year), higher stroke severity (1·08 [1·03–1·13] per point on NIHSS), lower educational attainment (1·16 [1·05–1·28] per year), acute phase cognitive impairment (5·86 [2·21–15·58]), lower Barthel Index (1·10 [1·05–1·16] per 5 points less), atrial fibrillation (1·91 [1·10–3·30]), metabolic syndrome (MetS, 2·05 [1·15–3·64]), particularly reduced high-density lipoprotein cholesterol (HDL-C, 2·61 [1·50–4·52]) and pre-/diabetes mellitus (2·13 [1·13–4·00]), imaging markers of small vessel disease, and stroke recurrence during follow-up (2·36 [1·16–4·83]). Patients who received acute reperfusion treatment had a 65% lower risk of PSD than those who did not (0·35 [0·16–0·77]). While factors related to the severity of the index stroke were more strongly associated with early-onset PSD, MetS showed a stronger association with delayed-onset PSD. The association between MetS and PSD was independent of stroke recurrence and consistent across age subgroups, with 5-year cumulative incidence ranging from 1·7% (0·0–4·0) in patients ≤65 years without MetS to 24·5% (14·3–33·4) in patients ≥74 years with MetS.

**Interpretation:**

The risk of dementia after stroke is multifactorial, with differing risk profiles for early-onset and delayed-onset PSD. Metabolic syndrome, including reduced HDL-C, emerged as a novel risk factor and potential target for PSD prevention.

**Funding:**

10.13039/501100005224German Center for Neurodegenerative Diseases (DZNE).

**Research in context**

**Evidence before this study**

We updated our previous systematic review on risk factors for post-stroke dementia (PSD) and cognitive impairment (PSCI), which originally searched MEDLINE and the Cochrane Library from database inception to Sept 15, 2023. The updated search added Embase and extended coverage to Dec 10, 2024. Eligible English-language articles reported associations between baseline risk factors and longitudinal PSD or PSCI risk. Search terms included “prospective”, “longitudinal”, “risk factors”, “stroke”, “dementia”, and “cognitive impairment”. While few baseline risk factors have been consistently identified in large, prospective cohort studies, robust evidence existed for older age, greater stroke severity, prior stroke, lower educational attainment, acute phase cognitive impairment, APOE-ε4 carrier status, lacunes, and white matter hyperintensities. Diabetes mellitus and atrial fibrillation were the most established vascular risk factors, but evidence for other modifiable factors remained inconclusive. The most robust evidence came from few population-based studies, which provide results that are more generalisable to the general stroke patient population. In contrast, reports from hospital-based studies, which allow for deeper phenotyping and identifying novel risk factors, were limited in quality and follow-up length. PSD incidence was highest early post-stroke, but data on risk factors for delayed-onset PSD (>6 months) were particularly limited, despite indications of differing mechanisms underlying early- and delayed-onset PSD.

**Added value of this study**

In this 5-year multicentre prospective hospital-based cohort of well-characterised patients with minor or major stroke, we used a standardised methodology for baseline and follow-up examinations, allowing precise evaluation of cognitive decline and dementia onset. Risk for PSD or PSCI varied substantially across sociodemographic, clinical, cardiometabolic, and neuroimaging factors. We identified a previously unrecognised association between PSD and metabolic syndrome, specifically its components diabetes and reduced HDL-C, independent of stroke recurrence. Patients who received acute reperfusion treatment had a significantly lower risk of PSD. The PSD incidence rate was 4·2 times higher in the early phase (3–6 months, 5·86/100 person-years) compared to the later phase (>6 months, 1·39/100 person-years). Early-onset PSD was predominantly linked to factors related to the stroke itself and prior brain health, while delayed-onset PSD was more strongly associated with cardiometabolic risk and stroke recurrence.

**Implications of all the available evidence**

The risk of post-stroke dementia and cognitive impairment is significantly influenced by factors related to poor pre-stroke brain health, greater stroke severity, vascular and metabolic risk, recurrent stroke, and cerebral small vessel disease. While the risk of PSD is highest early after stroke, a substantial risk persists over the long term. The importance of individual risk factors varies for early-onset PSD and delayed-onset PSD. Identifying these risk factors for PSD in the short- and long-term is essential for predicting individual risk, providing tailored counselling to patients and their families, and guiding the selection of participants for clinical trials. Cardiometabolic risk factors are associated with PSD regardless of stroke recurrence. These findings underscore the importance of focussing research efforts on modifiable risk factors and of prioritising dementia as a key outcome in clinical trials of secondary prevention in stroke patients.

## Introduction

Over the past three decades, global stroke mortality rates have steadily declined,^1^ shifting focus towards long-term outcomes following stroke.^2^, ^3^, ^4^ Cognitive impairment and dementia are among the most serious consequences, affecting patients, their families, and healthcare systems. Five to 40% of stroke survivors develop post-stroke dementia (PSD) within the first year, and 8–80% within 5 years, depending on risk profiles.^3^^,^^5^ A better understanding of the factors that predispose stroke patients to cognitive decline and dementia is needed to identify high-risk individuals, develop effective prevention and monitoring strategies, and counsel patients and caregivers.

A large-scale population-based study has shown that PSD incidence rates vary substantially with risk factors such as age, stroke severity, prior stroke, or APOE-ε4 genotype.^3^^,^^6^ Hospital-based prospective studies allow recruitment of well-characterised patient subgroups, deep risk factor profiling, detailed acute phase assessment, identification of novel risk factors, and standardised follow-up with comprehensive cognitive assessments. However, reliable results from studies with long-term follow-up remain limited.^4^^,^^7^

There is particular interest in risk factors that could be modified and explored in clinical trials. Currently, diabetes mellitus and atrial fibrillation are the most established modifiable risk factors for PSD, although their role in the development of cognitive impairment remains insufficiently understood.^3^^,^^5^^,^^7^^,^^8^ Many patients with diabetes also have a cluster of cardiometabolic risk factors, known as metabolic syndrome (MetS).^9^^,^^10^ MetS is diagnosed when three out of five markers are present: abdominal obesity, elevated triglycerides, reduced HDL-C, hypertension, and hyperglycaemia.^9^ MetS has been associated with higher risks of cardiovascular disease^10^ and dementia in population-based studies,^11^, ^12^, ^13^ but its role in PSD remains unexplored. With novel available treatments against metabolic dysfunction, such as obesity and diabetes, investigating how MetS components affect PSD risk could inform new preventive strategies for stroke survivors.

Dementia diagnosed between 3 and 6 months after stroke, termed early-onset PSD, has been primarily related to the severity of the vascular insult and reduced reserve or resilience (including factors such as age, cognitive reserve, atrophy, cerebral small vessel disease (SVD) burden, or previous brain injuries).^14^ However, even in patients who do not develop dementia in the first 6 months after stroke, a significant risk of delayed-onset PSD persists.^14^ Few studies have explored risk factors for delayed-onset PSD after excluding early-onset cases.^6^^,^^14^, ^15^, ^16^, ^17^, ^18^ These studies suggest delayed-onset PSD is mainly associated with imaging markers of SVD burden, while the role of stroke recurrence, other vascular factors, and contributing pathologies remains unclear.^14^, ^15^, ^16^, ^17^, ^18^ Patients at higher risk of delayed-onset PSD could particularly benefit from targeted preventive interventions,^14^ underscoring the importance of identifying the modifiable risk factors for delayed-onset PSD.

Here, we report the main results of the prospective hospital-based German Center for Neurological Diseases (DZNE) mechanisms of dementia after stroke (DEMDAS) study, which was designed to determine the risk factors for PSD and identify possible new targets for PSD prevention. We further sought to investigate the different risk factors for early-onset and delayed-onset PSD and to examine the prevalence and predictors of post-stroke cognitive impairment.

## Methods

### Study design

The DZNE Mechanisms of Dementia After Stroke study (DEMDAS) is a prospective, multicentre, hospital-based cohort study aimed at understanding the determinants and mechanisms of dementia after stroke. Initially launched as a pilot study at LMU Munich, Germany (recruiting 136 participants between May 2011 and November 2013), the study was expanded to include an additional 600 patients across six tertiary stroke centres in Munich, Berlin, Bonn, Göttingen, and Magdeburg, Germany (Table S1). Participants were recruited from January 2014 to January 2019 and followed up for 5 years after stroke. The study was conducted in accordance with the Declaration of Helsinki, and ethics approval was obtained at each participating site prior to the start of the study (ethics committee of the medical faculty, LMU Munich [035–11 and 201–13], ethics committee of the medical faculty, Rhenish Friedrich-Wilhelms-University, Bonn [116/13], ethics committee of the university medicine Göttingen [21/1/12], ethics committee of the Technical University Munich [93/14 S], ethics committee of the Otto-Von-Guericke-University at the medical faculty and the university hospital Magdeburg [66,13]; the site at Charité university medicine Berlin participated in the study with the ethics vote of the LMU Munich, according to the Professional Code of Conduct of the Berlin Medical Association of September 2009, Section 15 [2]). The DEMDAS study is registered at http://www.clinicaltrials.gov (NCT01334749) and the detailed methodologies have been previously described.^4^^,^^19^^,^^20^

### Participants

Participants aged 18 years or older were included if hospitalised at any of the participating study centres for an acute ischaemic or haemorrhagic stroke, defined as a focal neurological deficit with symptom onset within the last five days before admission combined with an acute ischaemic infarct as documented by either a diffusion-weighted imaging positive lesion on cranial magnetic resonance imaging (MRI) or a new lesion on a delayed computed tomography (CT) scan; or an intracerebral haemorrhage as documented on CT or MRI. Participants were required to have an available informant. Exclusion criteria included pre-stroke dementia or significant cognitive decline (Informant Questionnaire on Cognitive Decline in the Elderly [IQCODE] score >64),^21^ malignant disease with a life expectancy <3 years, MRI contraindications, cerebral venous thrombosis, traumatic haemorrhage, haemorrhage from vascular malformations, or isolated meningeal or intraventricular haemorrhage. Participants and their informants were re-examined in person at 6, 12, 36, and 60 months post-stroke by trained study nurses and clinicians. Written informed consent was obtained from all patients or their legal guardians before study entry.

### Procedures

At baseline, participants underwent standardised evaluations by a study clinician and a study nurse shortly after hospitalisation. These included interviews, clinical and cognitive assessments, laboratory tests, and neuroimaging.^4^^,^^19^^,^^20^ The data collected covered sociodemographic information, medical and family history, medication use, and physiological measurements (e.g., blood pressure, BMI). Sex was self-reported as male or female. Genetic ancestry was analysed by comparing participant genotype data against the 1000 Genomes Project (1kG) Phase 3 reference panel (Supplementary Methods). Acute-phase neurological, functional, and cognitive status were assessed using clinical scales (National Institutes of Health Stroke Scale [NIHSS], modified Rankin Scale [mRS], Barthel Index, Delirium Rating Scale) and cognitive screening tests (Mini-Mental State Examination [MMSE], Montreal Cognitive Assessment [MoCA]). Metabolic syndrome was defined as the presence of three or more predefined criteria (Supplementary Methods).^9^ As part of the study protocol, cranial 3-T MRIs were conducted within 3–5 days post-stroke in all patients whenever feasible, enabling the assessment of multiple neuroimaging variables, including brain volume, infarct volume, conventional SVD markers (lacunes, white matter hyperintensities, cerebral microbleeds, and perivascular spaces), and mean skeletonised mean diffusivity (MSMD, details in Supplementary Methods and as reported previously^4^).

To minimise attrition and bias related to dementia outcome assessment, follow-up visits were conducted at 6, 12, 36, and 60 months via in-person visits at the study centres, home visits, or, if needed, telephone or mail. Additional telephone interviews were performed at 3, 24, and 48 months. In-person follow-ups included comprehensive cognitive and functional evaluations, which are described in detail in the Supplementary Methods.

The primary outcome, post-stroke dementia (PSD), was defined according to the DSM-5 criteria for major neurocognitive disorder, encompassing all incident dementia regardless of cause or time of onset, as detailed in the Supplementary Methods. Cognitive outcomes at each follow-up were evaluated by a committee of neurologists and memory clinic physicians using a tiered protocol (Supplementary Methods). Dementia diagnosis dates were determined after reviewing all medical records, cognitive and functional test results, and reports from patients and/or informants. The secondary outcomes were early-onset PSD (diagnosed 3–6 months post-stroke), delayed-onset PSD (>6 months), and post-stroke cognitive impairment (PSCI). The distinction between early- and delayed-onset PSD was not part of the original study protocol,^19^ but was included following work published in 2016 by Mok and colleagues.^14^^,^^15^^,^^18^ Most cognitive recovery occurs within the first 6 months post-stroke, though improvement can continue up to 12 months and beyond.^22^ The 6-month cut-off reflects this clinically relevant early recovery window, but given the absence of a universally accepted threshold, we conducted a sensitivity analysis using a 12-month cut-off for early-onset PSD. PSCI was defined as the combined endpoint of dementia and mild cognitive impairment.^23^

### Statistical analysis

Baseline characteristics were compared between patients with and without PSD using two-tailed t-tests for normally distributed continuous variables, Wilcoxon-rank-sum tests for non-normally distributed continuous variables, or χ^2^ tests for categorical variables. We calculated the cumulative PSD incidence rates and 95% CIs using a Kaplan–Meier estimator accounting for the competing risk of death for the total sample and stratified by risk factors. To improve interpretability, age, education, and NIHSS score were categorised for these analyses (Supplementary Methods), and cumulative incidence rates were compared using Gray's test. Patients were censored at the last follow-up examination before they were lost to follow-up. The exact onset of dementia symptoms between follow-up visits was often unknown. Hence, we imputed onset dates using the mean interval between visits and conducted a sensitivity analysis with multiple imputation. We used standard and competing-risk Cox regression models to calculate cause-specific and subdistribution hazard ratios, respectively, evaluating the relationships between baseline factors and the 5-year risk of incident PSD.^24^ Models were adjusted for age, sex, education, and stroke severity,^3^^,^^5^ with death as the competing risk. The proportional hazards assumption was tested using the Grambsch and Therneau test.^25^ In case of violation (p < 0·05), we employed flexible parametric survival models with natural splines to model non-proportional hazards and time-varying effects (Table S19).^26^ For analysing associations with the secondary outcomes early-onset and delayed-onset PSD, we split follow-up into an early (≤6 months) and a later period (>6 months; Supplementary Methods). Patients with early-onset PSD were excluded from the analysis of delayed-onset PSD. Population attributable fractions (PAFs) for early- and delayed-onset PSD, along with their CIs, were estimated using bootstrap resampling (10,000 iterations, Supplementary Methods). Differences in PAFs between early- and delayed-onset PSD were calculated for each bootstrap iteration with 95% CIs derived from the 2·5th and 97·5th percentiles of the bootstrap distribution of PAF differences. Predictors for PSCI across the 5-year study period were assessed using generalised estimating equations (GEE) logistic regression models. All PSCI models were adjusted for age, sex, education, and stroke severity. We performed subgroup analyses of the PSD analysis stratified by sex. A priori and post-hoc power calculations are detailed in the Supplementary Methods. Sensitivity analyses included adjustments for acute stroke treatment, stroke recurrence, and acute phase cognitive impairment. p-values of <0·05 were considered statistically significant and we accounted for multiple comparisons using the false discovery rate (FDR) method for p-values derived from the main analyses. All statistical analyses were conducted in RStudio (version 2023·06·1).

### Role of the funding source

The funder of the study had no role in study design, data collection, data analysis, data interpretation, or writing of the report.

## Results

**Fig. 1:**
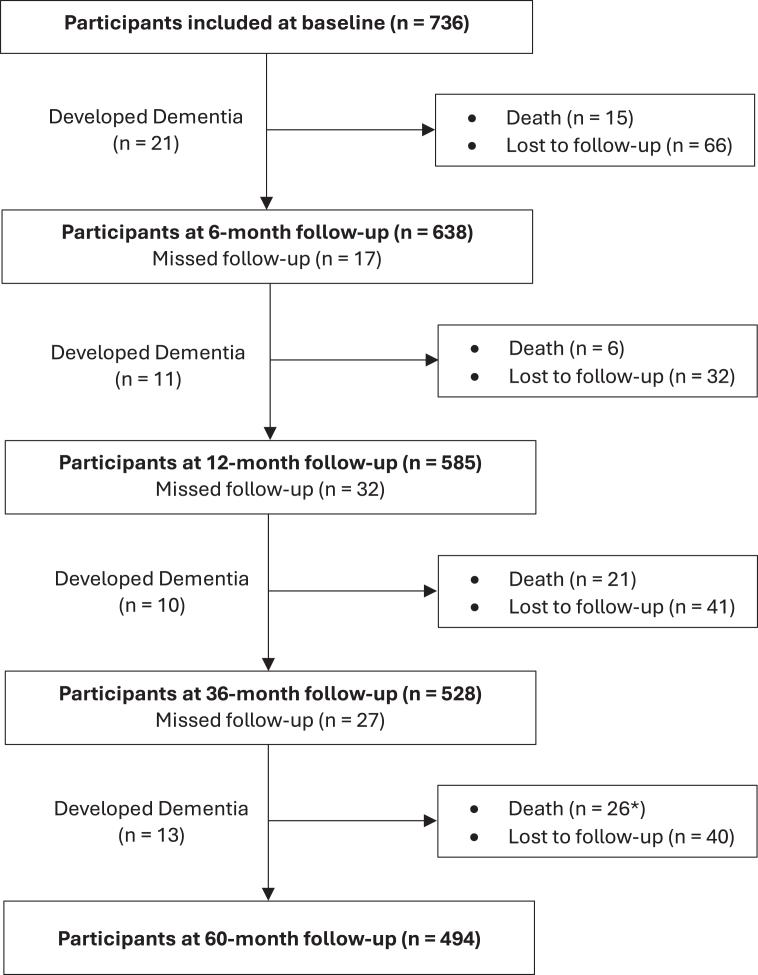
**Participant flow chart for the 5-year follow-up period.** Follow-ups via telephone at 3, 24, and 48 months exist but are not shown here. ∗Five deaths were recorded after participants were lost to follow-up.

Of 736 stroke patients recruited (mean age 68·0 [SD 11·2], 245 [33·3%] female), 706 (95·9%) underwent at least one follow-up examination and were included in the primary outcome analysis. A total of 557 (75·7%) patients were followed until death or end of study. Patient flow is detailed in Fig. 1, and baseline characteristics are presented in Table S1 (total sample) and in Table S2 (by sex). Missing values for baseline variables ranged from 0% (most clinical characteristics) to 19% (APOE genotype; Table S1). The median admission NIHSS score was 3 (IQR 1–5). 79 (10·7%) patients had a history of prior stroke, and 363 (49·3%) met the criteria for metabolic syndrome (MetS).

Patients were followed for a total of 2899 person-years (median 5·0 [IQR 3·3–5·1]), during which 68 (9·2%) died and 179 (24·3%) were lost to follow-up. Table S3 presents the number of patients who were lost to follow-up or died, broken down by study centre and follow-up period. Reasons for death or loss to follow-up are detailed in Table S4. Retained participants were younger, more educated, less dependent, had lower rates of hypertension and atrial fibrillation, better pre-stroke and acute phase cognitive performance, higher HDL-C, lower SVD burden, and greater brain volume than participants who died or were lost to follow-up (Table S5). The in-person follow-up visits occurred at median times of 6·2, 12·2, 36·3, and 60·5 months (Figure S1).

**Fig. 2:**
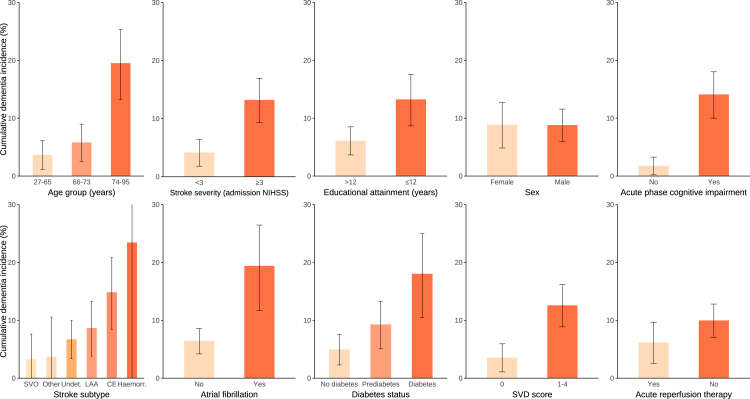
**Cumulative post-stroke dementia incidence stratified by different categorical baseline characteristics.** Acute phase cognitive impairment was defined as MoCA <26 or MMSE <27. Prediabetes was defined as HbA_1c_ ≥5·7 and <6·5. Diabetes mellitus was defined as HbA_1c_ ≥6·5 or treatment with antidiabetic medication. Acute reperfusion therapy indicates intravenous thrombolysis and/or endovascular thrombectomy. Error bars represent the 95% confidence interval for the Kaplan–Meier estimated cumulative incidence. Cumulative incidence rates were compared using Grey's test. CE = cardioembolism. Haemorr. = haemorrhagic stroke. LAA = large artery atherosclerosis. NIHSS = National Institutes of Health Stroke Scale. SVD = cerebral small vessel disease. SVO = small vessel occlusion. TOAST = Trial of Org 10172 in Acute Stroke Treatment. Undet. = stroke of undetermined aetiology.

During follow-up, 55 participants developed incident dementia (6-month incidence: 3·2% [1·8–4·5], 5-year incidence: 8·8% [95% CI 6·5–11·1]; Figures S3 and S4). Table 1 details baseline characteristics stratified by patients who did and did not develop PSD. Twenty patients with diagnosed dementia died before reaching the 5-year follow-up. Fig. 2 presents the 5-year cumulative incidence of PSD, stratified by key baseline categorical risk factors. PSD incidence was higher in the oldest age tertile (≥74 years; 19·5% [13·2–25·4]) than in the middle (66–73 years; 5·8% [2·5–9·0], p < 0·0001) and lowest tertiles (<66 years; 3·7% [1·1–6·1], p < 0·0001); among patients with admission NIHSS ≥3 compared to those with NIHSS <3 (13·2% [9·3–16·9] vs 4·2% [1·8–6·4], p = 0·0001); among patients with ≤12 years of educational attainment compared to those with more than 12 years (13·2% [8·7–17·6] vs 6·1% [3·6–8·5], p = 0·01); and among those with acute phase cognitive impairment (MoCA < 26 or MMSE < 27) compared to those without (14·1% [10·0–18·0] vs 1·7% [0·2–3·3], p < 0·0001). There were no significant differences in the unadjusted cumulative PSD incidence rates between female and male participants (8·9% [4·8–12·7] vs 8·8% [6·0–11·6], p = 0·99) and between those who did and did not receive acute reperfusion therapy (6·2% [2·6–9·7] vs 10·0% [7·0–12·8], p = 0·15). When stratified by stroke aetiology, patients with haemorrhagic stroke had the highest PSD incidence (23·5% [0·04–41·4]), followed by those with cardioembolic (14·9% [8·4–20·9]), large artery (8·7% [3·8–13·3]), undetermined (6·7% [3·4–10·0]), other aetiology (3·7% [0·0–10·6]), and small vessel stroke (3·3% [0·0–7·6], p = 0·006).

**Table 1:** Baseline characteristics of stroke survivors who did and did not develop post-stroke dementia.

	No PSD (n = 681)	PSD (n = 55)	p value
**Sociodemographic variables**			
Age (years)	67·3 ± 11·0	76·5 ± 9·3	<0·0001
Age ≥74 years	223 (32·7%)	38 (69·1%)	<0·0001
Female^a^	226 (33·2%)	19 (34·5%)	0·95
Male^a^	455 (66·8%)	36 (65·5%)	0·95
Education (years)	13 (12–16)	12 (11–13)	0·005
Education ≤12 years	262 (38·5%)	30 (54·5%)	0·03
**Genetic ancestry**^b^			1·00
European	552/554 (99·6%)	45/45 (100%)	
Ad mixed American	1 (0·2%)	0 (0·0%)	
East Asian	1 (0·2%)	0 (0·0%)	
**Clinical/cognitive acute phase deficits**			
Admission NIHSS score	2 (1–5)	4 (3–7)	0·001
Admission NIHSS ≥3	345 (50·7%)	42 (76·4%)	0·0004
Barthel index score	100 (85–100)	75 (55–90)	<0·0001
Delirium rating scale score	0 (0–1)	0 (0–1)	0·15
Acute phase MoCA score	25 (23–28)	21 (19–24)	<0·0001
Acute phase cognitive impairment^c^	338/660 (51·2%)	44/49 (89·8%)	<0·0001
**Cardiovascular risk factors**			
Hypertension	523 (76·8%)	48 (87·3%)	0·10
Diabetes mellitus	129 (18·9%)	21 (38·2%)	0·001
Dyslipidaemia	204 (30·0%)	25 (45·5%)	0·03
Current smoking	165 (24·2%)	6 (10·9%)	0·04
Regular alcohol consumption	517 (72·7%)	40 (71·4%)	0·71
Atrial fibrillation	126 (18·5%)	22 (40·0%)	0·0002
Prior history of stroke	68 (10·0%)	11 (20·0%)	0·04
Ischaemic heart disease	69 (10·0%)	12 (21·8%)	0·01
BMI (kg/m^2^)	27·1 ± 4·3	26·4 ± 4·2	0·23
Systolic blood pressure (mmHg)	139 (129–150)	146 (130–152)	0·17
Diastolic blood pressure (mmHg)	80 (71–86)	79 (73–85)	0·55
HbA_1c_ (%)	5·7 (5·4–6·1)	5·8 (5·5–6·7)	0·03
LDL cholesterol (mg/dL)	127 (104–153)	113 (89–154)	0·13
HDL cholesterol (mg/dL)	48 (40–58)	43 (36–58)	0·03
Triglycerides (mg/dL)	121 (91–167)	108 (88–207)	0·95
**Criteria for metabolic syndrome**^d^			
Abdominal obesity	363/641 (56·7%)	28/48 (58·3%)	0·94
Elevated triglycerides	215/640 (33·6%)	18/52 (34·6%)	1·00
Reduced HDL cholesterol	204/658 (31·0%)	27/52 (51·9%)	0·003
Elevated blood pressure	603/680 (88·7%)	50/55 (90·9%)	0·78
Prediabetes/diabetes mellitus	347/643 (54·0%)	39/53 (73·6%)	0·009
Metabolic syndrome (≥3 of the above components present)	329 (48·2%)	36 (65·5%)	0·02
**Index stroke classification**			
Ischaemic stroke	664 (97·5%)	51 (92·7%)	0·10
TOAST classification of acute ischaemic stroke subtype			0·03
Large artery atherosclerosis	154 (22·6%)	12 (21·8%)	–
Cardioembolism	144 (21·1%)	20 (36·4%)	–
Small artery occlusion	84 (12·3%)	2 (3·6%)	–
Other determined aetiology	28 (4·1%)	1 (1·8%)	–
Undetermined aetiology	254 (37·3%)	16 (29·1%)	–
Haemorrhagic stroke	17 (2·5%)	4 (7·3%)	0·10
**Acute stroke treatment**			
Intravenous thrombolysis (IVT)	178 (26·1%)	10 (18·2%)	0·30
Endovascular thrombectomy (EVT)	71 (10·4%)	7 (14·6%)	0·80
Any reperfusion therapy (IVT and/or EVT)	198 (29·1%)	11 (20·0%)	0·20
**Neuroimaging parameters**			
Normalised brain volume (%)	68·0 (64·6–71·8)	63·6 (61·4–66·3)	<0·0001
Infarct volume (mm^3^)	2248 (8520–11760)	2488 (600–14632)	0·68
Normalised stroke lesion volume (%)	0·15 (0·03–0·76)	0·17 (0·04–0·96)	0·63
Small vessel disease score			0·001
0	251/615 (40·8%)	8/51 (15·7%)	–
1	179/615 (29·1%)	22/51 (43·1%)	–
2	125/615 (20·3%)	11/51 (21·6%)	–
3	48/615 (7·8%)	6/51 (11·8%)	–
4	12/615 (1·9%)	4/51 (7·8%)	–
Lacune count	0 (0–0)	0 (0–0)	0·01
≥3 lacunes	7/618 (1·1%)	5/53 (9·4%)	0·0001
Normalised white matter hyperintensity volume (%)	0·21 (0·07–0·50)	0·43 (0·23–1·36)	<0·0001
Cerebral microbleed count	0 (0–0)	0 (0–0)	0·10
Perivascular space grade	1 (1–2)	2 (1–3)	0·004
Mean skeletonised mean diffusivity (z-score)	−0·19 (−0·78–0·51)	0·86 (−0·01-1·97)	<0·0001
**Genetic risk factors**			
APOE genotype			0·18
0 ε4 allele	431/551 (78·2%)	31/43 (72·1%)	–
1 ε4 allele	112/511 (20·3%)	10/43 (23·3%)	–
2 ε4 alleles	7/511 (1·3%)	2/43 (4·6%)	–
**Pre-stroke clinical/cognitive function**			
mRS before stroke	0 (0–0)	0 (0–0)	0·36
IQCODE score	48 (48–49)	49 (48–51)	0·002

Data are n (%), median (IQR), mean (SD), or n/N (%).

APOE = apolipoprotein E. BMI = body-mass index. EVT = Endovascular thrombectomy. HbA_1c_ = glycated haemoglobin. HDL = high-density lipoprotein. IQCODE = Informant Questionnaire on Cognitive Decline in the Elderly. IVT = Intravenous thrombolysis. LDL = low-density lipoprotein. MoCA = Montreal Cognitive Assessment. mRS = Modified Rankin Scale. NIHSS = National Institutes of Health Stroke Scale. TOAST = Trial of Org 10172 in Acute Stroke Treatment.

a Sex was self-reported as male or female.

b Genetic ancestry was analysed comparing participant genotype data against the 1000 Genomes Project (1kG) Phase 3 reference panel (Supplementary Methods).

c MoCA <26 or mini-mental state examination <27 when MoCA was not available (n = 73).

d Defined according to Alberti et al.^2^

Among cardiovascular risk factors, PSD incidence was higher in patients with atrial fibrillation than in those without (19·4% [11·7–26·5] vs 6·4% [4·2–8·6], p < 0·0001) and in patients with diabetes mellitus (18·0% [10·5–25·0]) than in those with prediabetes (9·3% [5·1–13·3], p = 0·02) and no diabetes (5·0% [2·3–7·6], p < 0·0001). Also, patients with signs of small vessel disease on MRI (SVD score ≥ 1) had a higher incidence of PSD than those without (12·6% [8·9–16·2] vs 3·5% [1·1–5·9], p = 0·0001).

**Fig. 3:**
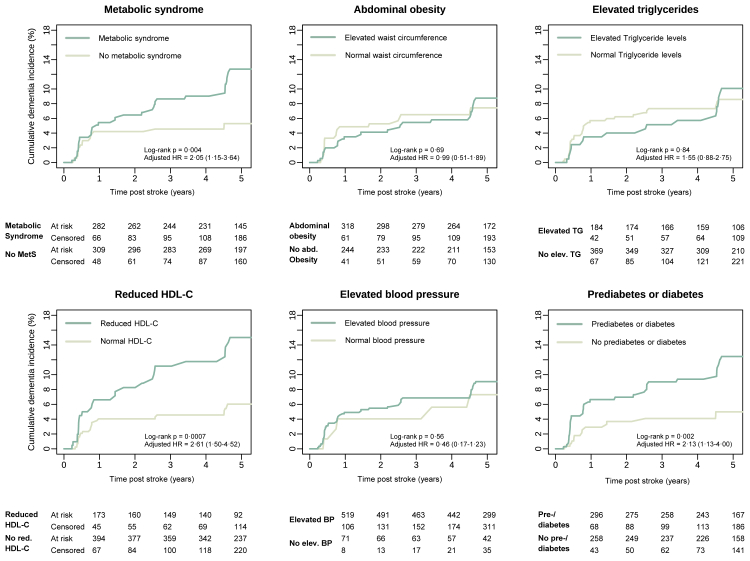
**Cumulative incidence curves for post-stroke dementia stratified by the presence of metabolic syndrome (top left panel) and individual metabolic syndrome components (top middle to bottom right panel).** Metabolic Syndrome was defined as the presence of three or more of the five criteria (Supplementary Methods).^9^ BP = Blood pressure. HDL-C = high-density lipoprotein cholesterol. MetS = Metabolic Syndrome. NIHSS = National Institutes of Health Stroke Scale. TG = Triglycerides.

**Fig. 4:**
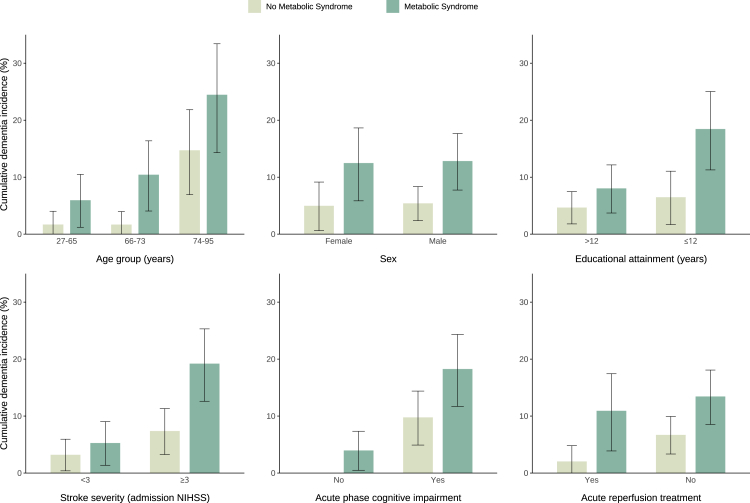
**Cumulative incidence rates for post-stroke dementia stratified by metabolic syndrome and other relevant baseline factors.** Error bars represent the 95% confidence interval for the Kaplan–Meier estimated cumulative incidence. Formal interaction tests showed no significant interactions (all p > 0·05). NIHSS = National Institutes of Health Stroke Scale.

Patients with MetS had a higher 5-year incidence of PSD compared to those without MetS (12·7% [8·7–16·5] vs 5·3% [2·8–7·7], p = 0·004; Fig. 3). This difference was maintained when further stratifying by age tertiles, sex, educational attainment, stroke severity, acute phase cognitive impairment, and acute reperfusion treatment (Fig. 4). Among the five MetS markers, the 5-year cumulative PSD incidence was significantly higher in patients with reduced HDL-C (15·0% [9·6–20·1] vs 6·0% [3·6–8·3], p = 0·0008) and prediabetes or diabetes mellitus (12·4% [8·6–16·1] vs 4·9% [2·3–7·6], p = 0·002), but did not differ significantly when stratifying by the remaining three MetS components (Fig. 3).

In Cox regression models (Table 2), older age and lower educational attainment were important sociodemographic risk factors for PSD. Further, patients with higher admission NIHSS scores, lower Barthel Index scores, lower MoCA scores, or acute phase cognitive impairment were at an increased PSD risk. Major vascular and metabolic risk factors included diabetes mellitus, atrial fibrillation, prior stroke, higher triglycerides, and MetS (≥3 components, per additional component, reduced HDL-C, and prediabetes or diabetes mellitus). Acute reperfusion therapy was associated with a lower PSD risk. Significant neuroimaging markers included lower normalised brain volume, higher lacune and cerebral microbleed count, greater normalised white matter hyperintensity volume, and higher mean skeletonised mean diffusivity. PSD risk was further related to APOE-ε4 homozygosity and recurrent stroke during follow-up. Results for PSCI aligned with those for PSD, with additional risk factors including lower HDL-C, higher infarct volume, and perivascular space grade (Table 2).

**Table 2:** Baseline factors associated with 5-year risk of incident post-stroke dementia (PSD) and post-stroke cognitive impairment (PSCI).

Risk factors	Post-stroke dementia				Post-stroke cognitive impairment		
	Cases/N	Adjusted hazard ratio (95% CI)	p value	FDR-p	Adjusted odds ratio (95% CI)	p value	FDR-p
**Sociodemographic factors**							
Age (per year)	55/706	1·13 (1·08–1·18)	<0·0001	<0·0001	1·03 (1·02–1·04)	<0·0001	<0·0001
Age ≥ 74	55/706	4·76 (2·65–8·55)	<0·0001	<0·0001	2·08 (1·69–2·60)	<0·0001	<0·0001
Female sex	55/706	0·47 (0·24–0·91)	0·02	0·05	0·99 (0·79–1·23)	0·91	0·91
Education (per year)	55/706	0·86 (0·78–0·95)	0·003	0·009	0·92 (0·89–0·96)	<0·0001	<0·0001
Education ≤ 12	55/706	1·89 (1·05–3·40)	0·03	0·06	1·83 (1·48–2·26)	<0·0001	<0·0001
**Clinical/cognitive acute phase deficits**							
Stroke severity (per point on admission NIHSS)	55/706	1·08 (1·03–1·13)	0·002	0·008	1·04 (1·02–1·06)	0·0008	0·002
Admission NIHSS ≥ 3	55/706	2·68 (1·44–4·97)	0·002	0·007	1·40 (1·14–1·71)	0·001	0·003
Barthel index (per 5 points)	55/704	0·90 (0·85–0·95)	<0·0001	0·0005	0·98 (0·97–0·98)	<0·0001	<0·0001
Delirious symptoms (per point on DRS)	55/706	1·17 (1·02–1·34)	0·03	0·05	1·06 (0·99–1·14)	0·09	0·12
Acute phase cognitive function (per point on MoCA)	41/625	0·83 (0·76–0·90)	<0·0001	0·0001	0·80 (0·77–0·83)	<0·0001	<0·0001
Acute phase cognitive impairment^a^	49/683	5·86 (2·21–15·58)	0·0004	0·002	3·17 (2·73–3·67)	<0·0001	<0·0001
**Vascular risk factors**							
Hypertension	55/706	1·05 (0·45–2·44)	0·92	0·95	0·92 (0·71–1·18)	0·49	0·52
Diabetes mellitus	55/706	2·28 (1·33–3·91)	0·003	0·009	1·62 (1·28–2·06)	<0·0001	0·0002
Dyslipidaemia	55/706	1·35 (0·77–2·34)	0·29	0·38	1·09 (0·88–1·34)	0·43	0·47
Current smoking	55/706	0·85 (0·36–1·97)	0·70	0·80	1·12 (0·87–1·44)	0·39	0·44
Regular alcohol consumption	55/706	0·73 (0·40–1·33)	0·30	0·38	0·89 (0·70–1·13)	0·33	0·38
Atrial fibrillation	55/706	1·91 (1·10–3·30)	0·02	0·04	1·60 (1·24–2·08)	0·0004	0·0009
Prior history of stroke	55/706	2·05 (1·08–3·88)	0·03	0·05	1·46 (1·08–1·97)	0·01	0·03
Ischaemic heart disease	55/706	1·98 (1·04–3·76)	0·04	0·06	1·81 (1·34–2·43)	<0·0001	0·0003
BMI (per 5 units [kg/m^2^])	55/706	0·98 (0·63–1·52)	0·93	0·95	1·01 (0·99–1·04)	0·31	0·36
Systolic blood pressure (per 10 mmHg)	55/701	1·01 (0·88–1·17)	0·84	0·94	0·92 (0·88–0·97)	0·003	0·005
Diastolic blood pressure (per 10 mmHg)	55/701	1·05 (0·86–1·28)	0·63	0·74	0·89 (0·85–0·94)	<0·0001	<0·0001
HbA_1c_ (per %)	52/658	1·06 (0·99–1·14)	0·09	0·15	1·04 (1·00–1·10)	0·08	0·11
LDL cholesterol (per 10 mg/dL)	53/684	1·01 (0·93–1·08)	0·88	0·94	1·00 (1·00–1·00)	0·11	0·15
HDL cholesterol (per 10 mg/dL)	52/679	0·81 (0·62–1·05)	0·11	0·17	0·89 (0·84–0·95)	0·01	0·03
Triglycerides (per 10 mg/dL)	52/663	1·03 (1·01-1·06)	0·02	0·04	1·01 (1·00–1·02)	0·07	0·11
**Metabolic syndrome components**^b^							
Abdominal obesity	48/666	0·99 (0·51–1·89)	0·97	0·97	1·17 (0·94–1·46)	0·15	0·20
Elevated triglycerides	52/663	1·55 (0·88–2·75)	0·13	0·19	1·22 (0·97–1·53)	0·09	0·12
Reduced HDL cholesterol	52/679	2·61 (1·50–4·52)	0·0006	0·003	1·25 (1·00–1·55)	0·05	0·08
Elevated blood pressure	55/705	0·46 (0·17–1·23)	0·12	0·18	0·67 (0·49–0·92)	0·01	0·03
Prediabetes or diabetes mellitus	53/666	2·13 (1·13–4·00)	0·02	0·04	1·27 (1·02–1·57)	0·03	0·05
Metabolic syndrome (≥3 of the above components present)	55/706	2·05 (1·15–3·64)	0·01	0·04	1·13 (0·92–1·38)	0·25	0·30
Per count of components increase	55/706	1·30 (1·04–1·63)	0·02	0·04	1·05 (0·97–1·14)	0·23	0·29
**Index stroke classification**							
Ischaemic stroke	55/706	1	0·06	0·09		0·72	0·74
Haemorrhagic stroke	55/706	2·69 (0·97–7·43)	–	–	1·11 (0·63–1·94)	–	–
**Acute stroke treatment**							
Any reperfusion therapy (IVT and/or EVT)	55/706	0·35 (0·16–0·77)	0·009	0·03	0·50 (0·37–0·67)	<0·0001	<0·0001
**Neuroimaging parameters**							
Normalised brain volume (per SD)	50/634	0·60 (0·41–0·89)	0·01	0·03	0·65 (0·56–0·75)	<0·0001	<0·0001
Normalised infarct volume (per SD)	50/634	1·19 (0·93–1·51)	0·16	0·22	1·12 (1·02–1·23)	0·02	0·04
Total small vessel disease score (per SD)	51/642	1·25 (0·90–1·73)	0·18	0·24	1·27 (1·13–1·42)	<0·0001	0·0002
Lacune count (per SD)	53/647	1·36 (1·26–1·47)	<0·0001	<0·0001	1·38 (1·20–1·60)	<0·0001	<0·0001
Presence of ≥3 lacunes	53/647	11·00 (4·92–24·60)	<0·0001	<0·0001	8·20 (3·36–20·01)	<0·0001	<0·0001
Normalised WMH volume (per SD)	48/633	1·42 (1·19–1·68)	<0·0001	0·0005	1·50 (1·32–1·70)	<0·0001	<0·0001
Cerebral microbleed count (per SD)	51/642	1·17 (1·07–1·27)	0·0008	0·004	1·03 (0·95–1·13)	0·44	0·47
Perivascular space grade (per SD)	53/646	1·23 (0·93–163)	0·14	0·20	1·15 (1·02–1·28)	0·02	0·03
Mean skeletonised mean diffusivity (per SD)	45/606	1·94 (1·39–2·70)	<0·0001	0·0006	1·76 (1·55–2·00)	<0·0001	<0·0001
**APOE genotype**							
0 ε4 alleles	43/563	1	–	–	1	–	–
1 ε4 allele	43/576	1·11 (0·52–2·36)	0·78	0·87	0·90 (0·76–1·06)	0·21	0·26
2 ε4 alleles	43/576	4·94 (1·36–11·79)	0·01	0·04	2·81 (1·74–4·54)	<0·0001	<0·0001
**Pre-stroke clinical/cognitive function**							
Modified Rankin Scale score before stroke	55/706	1·10 (0·75–1·62)	0·62	0·74	1·15 (0·98–1·36)	0·09	0·12
IQCODE score	49/655	1·07 (0·88–1·31)	0·48	0·58	1·07 (1·00–1·14)	0·04	0·06
**Recurrent events**							
Stroke recurrence	55/757	2·36 (1·16–4·83)	0·02	0·04	–	–	–

Associations with PSD were calculated using cox proportional hazards models with death as a competing risk. Associations with PSCI across the 6-, 12-, 36-, and 60-month follow-ups were calculated with logistic regression models using generalised estimating equations (GEE). Hazard ratios and odds ratios were adjusted for age, sex, education, and admission NIHSS score. At the 6-, 12-, 36-, and 60-month follow-ups, 180 (24·4%), 132 (17·9%), 102 (13·9%), and 112 (15·2%) participants had PSCI, respectively. The analysis for stroke recurrence could only be performed for the PSD endpoint and included only cases that were dementia-free at the time of the recurrent stroke.

APOE = apolipoprotein E. BMI = body-mass index. DRS = Delirium Rating Scale. EVT = Endovascular thrombectomy. HbA_1c_ = glycated haemoglobin. HDL = high-density lipoprotein. IQCODE = Informant Questionnaire on Cognitive Decline in the Elderly. IVT = Intravenous thrombolysis. LDL = low-density lipoprotein. MoCA = Montreal Cognitive Assessment. NIHSS = National Institutes of Health Stroke Scale.

a MoCA <26 or mini-mental state examination <27 when MoCA was not available (n = 73).

b Defined according to Alberti et al.^9^

Of the 55 incident dementia cases, 34 (61·8%) were classified as delayed-onset PSD. At baseline, patients who developed delayed-onset PSD had significantly higher MoCA scores compared to those with early-onset PSD (Table S6). Associations of baseline risk factors with early-onset and delayed-onset PSD are presented in Table S7. Risk factors significantly associated with early-onset PSD that did not reach statistical significance for delayed-onset PSD included atrial fibrillation, prior stroke, higher Delirium Rating Scale score, lower brain volume, and higher infarct volume (Table S7). Conversely, risk factors significantly associated with delayed-onset PSD that did not reach statistical significance for early-onset PSD included lower educational attainment, MetS, reduced HDL-C, higher triglyceride levels, acute reperfusion therapy, and greater WMH volume (Table S7). Flexible parametric survival models revealed time-varying relationships of Delirium Rating scale score and MetS (Figure S7 and Table S19), in line with the analyses stratifying by early and delayed onset.

**Fig. 5:**
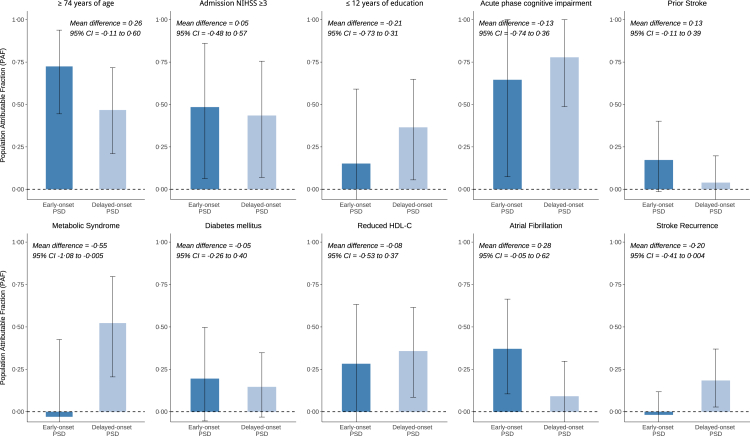
**Population attributable fractions (PAF) for different risk factors for early- and delayed-onset post-stroke dementia, defined as dementia that occurred between 3 and 6 or after 6 months, respectively.** Acute phase cognitive impairment was defined as MoCA <26 or MMSE <27, and metabolic syndrome as presence of three or more commonly used criteria (Supplementary Methods and Alberti et al.^9^). Error bars represent 95% confidence intervals, derived from 10,000 bootstrap iterations. HDL-C = high-density lipoprotein cholesterol. NIHSS = National Institutes of Health Stroke Scale.

Main contributors to early-onset PSD were age ≥74 years, acute phase cognitive impairment, admission NIHSS ≥3, and atrial fibrillation, while the main contributors to delayed-onset PSD were age ≥74 years, acute phase cognitive impairment, MetS, and admission NIHSS ≥3 (Fig. 5). Bootstrapped CIs were wide but indicated a stronger contribution of MetS to delayed-onset PSD compared to early-onset PSD.

Female participants were older, had fewer years of education, less frequently had acute phase cognitive impairment, and more frequently had abdominal obesity and cardioembolic stroke (Table S2). Sex-stratified analyses of PSD risk (Table S8) revealed overall similar trends but were likely underpowered, especially for women. Among men, age ≥74 was associated with a 6·4-fold increased risk of PSD, compared to a 3-fold increase in women. Diabetes mellitus, prior ischaemic heart disease, admission NIHSS ≥3, and educational attainment ≤12 years were strong predictors for PSD in men, but not women, whereas atrial fibrillation and pre-stroke IQCODE were strong predictors of PSD in women but not in men.

During follow-up, 56 (7·6%) patients experienced at least one recurrent stroke (Figure S5, Table S8); 10 (17·9%) developed dementia afterwards, while three (5·4%) had developed dementia before recurrence. Recurrent stroke before dementia diagnosis was associated with higher 5-year and delayed-onset PSD risk (HR 2·36 [1·16–4·83] and 3·94 [1·76–8·82], respectively; Table S9). Sensitivity analyses confirmed overall consistent associations between baseline variables and PSD risk, even after adjusting for acute reperfusion treatment, recurrent stroke, or acute phase cognitive impairment (Tables S10–S15) and when using 12 months as the cut-off for early- vs delayed-onset PSD (Table S16), as well as after multiple imputation for dementia onset date (Table S17). After adjusting for acute treatment, admission NIHSS emerged as a strong predictor for both early- and delayed-onset PSD. The associations of PSD with atrial fibrillation, prediabetes/diabetes, and MetS were also strengthened, while the associations with prior stroke and APOE-ε4 homozygosity were attenuated.

## Discussion

This study not only provides estimates of the association between reported risk factors and 5-year PSD risk, but also highlights a previously unrecognised association with metabolic syndrome (MetS), particularly its components reduced HDL-C and pre-/diabetes. MetS was a risk factor for delayed-onset PSD (>6 months), but not for early-onset PSD (≤6 months). Conversely, early-onset PSD was more strongly associated with older age, factors related to the stroke and its severity, and atrial fibrillation than delayed-onset PSD. Collectively, our findings highlight the multifactorial nature of PSD risk and emphasise time-dependent differences in the importance of individual risk factors.

We identified a set of binary risk factors, each of which was strongly associated with an increased PSD risk (HRs >2): age ≥74 years, admission NIHSS ≥3, acute phase cognitive impairment, diabetes mellitus, MetS, reduced HDL-C, presence of ≥3 lacunes, and stroke recurrence. Additionally, acute stroke treatment was associated with a 65% lower risk of PSD. These findings could inform both the development of prediction tools for long-term PSD risk and the selection of patients for PSD prevention trials. Overall, our results emphasise poor prior brain health, greater stroke severity, cardiometabolic risk factors, recurrent stroke, and SVD as the key contributors to PSD risk, which is largely consistent with previous findings.^3^^,^^5^^,^^7^ Modifiable risk factors are particularly relevant for designing secondary prevention trials and were therefore a focus in our analysis.

Baseline MetS was associated with a twofold increase in the risk of 5-year PSD and a 3·5-fold increase in the risk of delayed-onset PSD. This effect was independent of stroke recurrence and consistent across subgroups of age, with 5-year cumulative incidence rates ranging from 1·7% in younger patients (≤65 years) without MetS to 24·5% in older patients (≥74 years) with MetS. Reduced HDL-C and diabetes mellitus were the two most important individual MetS components contributing to this association. However, we also found a 30% increase in PSD risk with each additional MetS component, suggesting a potential dose-dependent relationship that extends beyond the effects of these two factors. To the best of our knowledge, the relationship between MetS and dementia has not been studied in the post-stroke setting, although MetS has been recognised as a potentially modifiable risk factor for all-cause dementia,^11^^,^^13^^,^^27^^,^^28^ vascular dementia,^27^^,^^28^ and Alzheimer's disease.^27^^,^^28^ The prevalence of MetS in our cohort (49·3%) was about twice as high as that in the European general population,^29^ but comparable to other stroke cohorts of similar age.^30^^,^^31^

Diabetes mellitus is an established modifiable risk factor for PSD,^3^^,^^5^^,^^7^^,^^14^^,^^15^^,^^17^^,^^18^ that contributes primarily by exacerbating vascular complications.^8^ Although it remains uncertain whether diabetes management reduces dementia risk, a recent study that combined RCT data and Danish nationwide registry data suggested a beneficial effect of glucagon-like peptide-1 (GLP-1) on dementia risk in patients with type 2 diabetes.^32^ This approach should also be investigated in stroke patients with diabetes. Considering recent findings,^33^ it is further worth exploring whether GLP-1 or dual GIP/GLP-1 receptor agonists could prevent dementia in stroke patients with prediabetes and obesity by preventing the progression to diabetes. Given the high prevalence of MetS in our and other stroke cohorts, such therapeutic strategies could hold potential for PSD prevention, particularly if future studies confirm the role of cardiometabolic risk factors in long-term cognitive decline.

We found that the relationships between cardiometabolic risk factors and PSD remained robust with minimal changes in effect sizes after adjusting for recurrent stroke. While most secondary prevention trials use stroke recurrence as the single neurological endpoint,^34^ our findings suggest that the relationship between PSD and modifiable factors like diabetes mellitus and MetS is largely independent of stroke recurrence. This highlights the importance of including dementia as a primary outcome in secondary prevention trials for stroke patients.

Compared to previous studies, the incidence and prevalence of PSD in our cohort were substantially lower.^3^^,^^35^ For example, the 1-year cumulative incidence was approximately 5% in our study versus 17% in OxVasc.^3^ Several differences in study design and sample characteristics likely explain this discrepancy. First, OxVasc included all acute vascular events in Oxfordshire, capturing patients with severe strokes who may not have reached tertiary care or were managed in community settings. By contrast, DEMDAS enrolled only patients referred to tertiary stroke centres, likely underrepresenting such cases. Second, nearly 30% of our participants received acute reperfusion therapy, including thrombolysis and thrombectomy, which became standard practice in the early to mid-2000s and after 2015, respectively, and were infrequently used in earlier cohorts. In our study, patients who received reperfusion therapy had a 65% lower risk of PSD than those who did not. Although observational, this finding suggests that timely treatment may lower long-term dementia risk. Third, our cohort also had a younger median age (68 vs 73 years in the general European stroke population^36^) and lower NIHSS scores at admission, both known predictors of PSD, which may further explain the lower observed incidence and reflect selection effects inherent to our study population.

Our findings suggest sex-specific differences in the risk profile for PSD. Although women in our sample were older and had lower educational attainment, they had a lower overall risk of PSD compared to men. This may reflect weaker associations of age, education, stroke severity, and vascular risk factors, particularly diabetes and ischaemic heart disease, with PSD in women. Conversely, atrial fibrillation was more strongly associated with PSD in women, possibly due to their higher rate of cardioembolic stroke, which has been reported previously.^37^ A similar sex difference was found in a study from the U.S. National Alzheimer's Coordinating Center (NACC) cohort.^38^ These results underscore the importance of considering sex differences for individual risk prediction and clinical trial design.

In our study, dementia incidence was higher in the early compared to the late phase post-stroke, but more than 60% of PSD cases manifested with a delayed onset. The low prevalence of severe strokes in our sample likely contributed to the smaller proportion of early-onset PSD.^3^^,^^14^ Importantly, PSD risk remained elevated beyond the early phase after stroke, across all stroke severity levels, as was also apparent in 5-year data from the OxVasc study.^3^ Overall, these findings emphasise a persistent PSD risk beyond the acute phase, underscoring the need to understand long-term cognitive trajectories.

Our results imply a difference in the importance of baseline risk factors for early- compared to delayed-onset PSD. In keeping with previous findings,^3^^,^^5^^,^^7^^,^^14^^,^^17^ we found early-onset PSD to show stronger associations with acute stroke-related deficits and parameters related to prior reserve or resilience. In contrast, delayed-onset PSD was more strongly associated with MetS, reduced HDL-C, diabetes mellitus, acute phase cognitive impairment, and lower educational attainment. Results from our PAF analyses indicated that MetS contributed to 53% of delayed-onset PSD cases, exceeding the PAF for age ≥74 years. Whether post-stroke interventions targeting MetS or its components reduces long-term PSD remains unknown, but identifying high-risk patients opens opportunities for targeted interventions. Recurrent stroke was associated with delayed-onset, but not with early-onset PSD, which aligns with some, but not all, previous studies.^14^, ^15^, ^16^^,^^18^ Discrepancies may be partly explained by a slightly higher incidence of recurrent stroke in our cohort than in others.^16^^,^^18^

Atrial fibrillation was associated only with early-onset PSD in our study, with a stronger overall association observed in women, consistent with recent findings from the NACC.^38^ While previous reports on the association between atrial fibrillation and PSD have been inconsistent,^7^ our results suggest that its role may be more pronounced early after stroke and potentially modulated by sex-specific factors. Hypertension, a risk factor for recurrent stroke,^1^ was not associated with a higher PSD risk in our cohort, consistent with previous meta-analyses.^5^^,^^7^ This may reflect good baseline blood pressure control among the participants.

The association between lower HDL-C and PSD became apparent only when using sex-specific cut-offs for HDL-C (<40 mg/dL for males, <50 mg/dL for females) related to MetS. Low HDL-C has been identified as a risk factor for Alzheimer's Disease in Mendelian randomisation meta-analyses and the Framingham Heart Study.^39^^,^^40^ Possible mechanisms linking HDL-C to dementia include its vascular-protective, anti-inflammatory, and cholesterol efflux-enhancing properties,^8^^,^^41^ which could support post-stroke recovery and mitigate chronic vascular injury, such as SVD.^42^ Future studies should investigate whether the relationship between low HDL-C and PSD is mediated by progressive SVD burden.

Our results emphasise the importance of SVD as a predictor of both early- and delayed-onset PSD.^4^^,^^14^^,^^15^^,^^43^ The relationship was evident for both conventional SVD markers (lacune count, WMH volume, and CMB count) and MSMD, a marker sensitive to early microvascular injury. Of twelve patients who presented with ≥3 lacunes on baseline MRI, five developed PSD, corresponding to an 11·3 times higher PSD risk compared to patients with 0–2 lacunes. Clinical trials are needed to assess if targeting SVD progression improves post-stroke cognitive outcomes.^44^ Our findings further suggest that even mild delirium symptoms are associated with PSCI and early-onset PSD, reinforcing the role of acute phase impairments in early-onset PSD.^14^ The weaker overall association may be due to the mild symptom burden in our cohort and the limited number of patients meeting criteria (DRS ≥ 10, n = 4) for a clinical diagnosis of delirium, which has previously been linked to cognitive decline.^45^

Strengths of this study include its prospective, multicentre design with regular follow-ups across five years, the large sample size, standardised clinical and imaging protocols, central monitoring, and rigorous procedures maintained for baseline, follow-up, and end-point assessments. This study also has limitations. First, due to the demanding study protocol, which included serial MRI scanning, detailed cognitive testing, and the requirement of an informant, patients with milder strokes were overrepresented. However, this also reflects a population that is most likely to benefit from interventions targeting long-term outcomes. Our findings are limited to a highly selected hospital-based study cohort and require replication in larger, more inclusive, population-based, and ethnically more diverse cohorts to achieve generalisability. Second, the attrition rate was comparably high, which may have introduced bias. At baseline, patients lost to follow-up had poorer brain health, greater acute phase impairment, and more cardiovascular comorbidity, potentially limiting the generalisability of our findings to healthier stroke survivors. Third, as cognitive, health, and mortality data could not be obtained for many patients who revoked consent, PSD incidence, PSCI prevalence, and mortality may have been underestimated. Fourth, we were unable to perform subgroup analyses by dementia subtype. Although initially planned, difficulties in obtaining definitive diagnoses for dementia subtypes and the limited statistical power due to the low number of dementia cases led us to exclude these analyses. Additionally, we chose to discontinue amyloid-β positron emission tomography (PET) imaging after an interim analysis on 56 patients.^46^ Fifth, female participants were underrepresented, which mirrors a broader issue in stroke studies.^47^ This imbalance may have been influenced by factors such as greater disability and lower likelihood of having an informant among women, which could limit generalisability of the findings across sexes. Lastly, PAF estimates should be interpreted cautiously due to the observational nature of the study and uncertain causality.

Altogether, our findings suggest that while acute stroke care is critical for mitigating early-onset dementia risk, sustained efforts to monitor and manage cardiometabolic risk factors are needed to lower PSD risk in the long run. Cardiometabolic risk factors may contribute to delayed-onset PSD through mechanisms beyond recurrent vascular events, highlighting the importance of including PSD as a key outcome for clinical post-stroke trials. Further studies should explore whether targeting metabolic dysfunction reduces long-term PSD risk.

## Contributors

JF contributed to data preparation and interpretation, performed the statistical analysis, and drafted the manuscript. MKG critically reviewed and edited the manuscript; contributed to data interpretation; provided advice on the analyses; and was part of the endpoint committee for ascertaining dementia cases. DJ ascertained dementia cases as part of the endpoint committee. MDu conceptualised the neuroimaging protocol and established the central imaging platform. RF contributed significantly to data preparation. AD contributed to data preparation, description of MRI methods, and provided advice on the analysis of the MRI data. MKG, FB, SS, CK, PH, CHN, TGL, KB, and BI contributed to data acquisition as study physicians. LK managed the on-site study coordination. MW contributed to conceptualisation of the neuropsychological test battery. AS administered the clinical research platform of DZNE. KW coordinated the study and contributed to data collection, cleaning, preparation, and quality control. GP, IZ, ME, SW, and MG contributed to the conception, design, and funding acquisition for the DEMDAS study, as well as to data collection. MDi contributed to data interpretation; co-wrote the manuscript; was part of the endpoint committee; and initiated, designed, obtained funding for, and coordinated the DEDEMAS-DEMDAS study. All authors had full access to all the data in the study, approved the final version of the manuscript, and had final responsibility for the decision to submit for publication. JF, MKG, and MDi take full responsibility for the reported results, having verified the data and ensured the integrity of the data and the accuracy of the analyses.

## Data sharing statement

Upon publication, de-identified participant data and software code will be made available to researchers upon reasonable request to martin.dichgans@med.uni-muenchen.de.

## Declaration of interests

Dr. Georgakis reports consulting for Tourmaline Bio and the Gerson Lehrman Group (GLG), all outside the submitted work. Dr. Endres reported receiving grants from Bayer and fees paid to the Charité – Universitätsmedizin Berlin from Amgen, AstraZeneca, Bayer Healthcare, Boehringer Ingelheim, BMS, Daiichi Sankyo, Sanofi, and Pfizer, all outside the submitted work. Dr. Wunderlich reports being part of the steering committees of DEMDAS, German Stroke Registry, and ARCTIC-I (ESAIC-CTN), being a member of the guidelines commission “Post-stroke care” (DGN), and fees paid to the Technical University Munich from Philips, Phenox, Abbott, and MicroVention, all outside the submitted work. Dr. Zerr reports consulting for IONIS, outside the submitted work. Dr. Nolte reports honoraria for lectures from Alexion, Astra Zeneca, Bayer, BMS, Novartis, and Pfizer, a payment for a testimony at the Hanseatisches Oberlandesgericht Hamburg, Germany, and being a member of the guidelines committee of the European Stroke Organisation (ESO), all outside the submitted work. Dr. Dichgans reports consulting for Woolsey pharmaceuticals and NEUVASQ Biotechnologies SA, an issued patent “Means and methods for determining the potential extent of brain injury” (PCT/EP2024/075417), being an unpaid member of the steering committee of DEMDAS, DGN, AHA/ASA, ESO, German Center for Cardiovascular Research (DZHK), and being an unpaid fellow of the EAN and WSO, all outside the submitted work, and having a paid personal contract as a Principal Investigator with the German Center for Neurodegenerative Diseases (DZNE). All other authors declare no conflicts of interest.

## Appendix A Supplementary data

Supplementary Materials

DEMDAS Investigators

German Abstract
