# Supplementary material for: Risk factors for dementia and cognitive impairment within 5 years after stroke: a prospective multicentre cohort study
Source: Lancet Reg Health Eur. 2025 Aug 19;56:101428. doi: 10.1016/j.lanepe.2025.101428 (PMC12396445; doi:10.1016/j.lanepe.2025.101428)
Supplement: Supplementary Materials [file mmc1.pdf]

Supplementary Material

Risk factors for dementia and cognitive impairment within 5 years after stroke: a prospective multicentre cohort study

Authors: Jule Filler[1,2], Marios K Georgakis[1,3,4], Daniel Janowitz[1], Marco Duering[1,5], Rong Fang[1], Anna Dewenter[1], Felix Bode[6,7], Sebastian Stoesser[6,7], Christine Kindler[6,7], Peter Hermann[8], Christian H Nolte[9,10,11,12,13], Thomas G Liman[12,9,14], Lucia Kerti[12,9], Kathleen Bernkopf[15], Benno Ikenberg[15], Wenzel Glanz[16,17], Michael Wagner[6,18], Annika Spottke[6,7], Karin Waegemann[1,19], Michael Goertler[16,17], Silke Wunderlich[15], Matthias Endres[12,9,13,20,21], Inga Zerr[8,22], Gabor C Petzold[6,7], Martin Dichgans[1,19,4,23]

Affiliations:
[1]Institute for Stroke and Dementia Research (ISD), LMU University Hospital, LMU Munich, Munich, Germany
[2]Graduate School for Systemic Neurosciences, Ludwig-Maximilians-University, Munich, Germany
[3]Program in Medical and Population Genetics, Broad Institute of MIT and Harvard, Cambridge, Massachusetts, USA
[4]Munich Cluster for Systems Neurology (SyNergy), Munich, Germany
[5]Medical Image Analysis Center (MIAC AG) and Department of Biomedical Engineering, University of Basel, Basel, Switzerland
[6]German Center for Neurodegenerative Diseases (DZNE), Bonn, Germany
[7]Department of Vascular Neurology, University Hospital Bonn, Bonn, Germany
[8]Department of Neurology, University Medical Center Göttingen, Göttingen, Germany
[9]German Center for Neurodegenerative Diseases (DZNE, Berlin), Berlin, Germany
[10]Department of Neurology with Experimental Neurology, Charité - Univeristätsmedizin Berlin, Berlin, Germany
[11]Berlin Institute of Health (BIH), Berlin, Germany
[12]Center for Stroke Research Berlin (CSB), Charité – Universitätsmedizin Berlin, Berlin, Germany
[13]German Centre for Cardiovascular Research (DZHK), partner site Berlin, Berlin, Germany
[14]Department of Neurology, Carl Von Ossietzky University, Oldenburg, Germany
[15]Department of Neurology, TUM School of Medicine, Technical University of Munich, Munich, Germany
[16]Department of Neurology, University Hospital, Otto-von-Guericke University Magdeburg, Magdeburg, Germany
[17]German Center for Neurodegenerative Diseases (DZNE), Magdeburg, Germany
[18]Department of Old Age Psychiatry and Cognitive Disorders, University Hospital Bonn, Bonn, Germany
[19]German Center for Neurodegenerative Diseases (DZNE, Munich), Munich, Germany
[20]German Center for Mental Health (DZPG), partner site Berlin, Berlin, Germany
[21]Klinik und Hochschulambulanz für Neurologie, Charité – Universitätsmedizin Berlin, Berlin, Germany
[22]Cerman Center for Neurodegenerative Diseases (DZNE), Göttingen, Germany
[23]German Centre for Cardiovascular Research (DZHK, Munich), Munich, Germany

Corresponding author:
Prof Martin Dichgans MD, Institute for Stroke and Dementia Research, University Hospital of Ludwig-Maximilians-University (LMU), Munich 81377, Germany
e-mail: martin.dichgans@med.uni-muenchen.de

Supplementary Methods 4
Figure S1: Distribution of time since stroke for each follow-up time point 12
Figure S2: Genetic ancestry of the study cohort confirmed by PCA with 1000 genomes 13
Figure S3: Cumulative incidence curve for dementia over 5 years post stroke 14
Figure S4: Cumulative incidence for dementia and death over 5 years post stroke 15
Figure S5: Cumulative incidence curve for stroke recurrence over 5 years post stroke 16
Figure S6: Proportion of endpoints per follow-up time point 17
Figure S7: Time-varying associations with PSD risk 18
Table S1: Baseline characteristics of the entire DEDEMAS-DEMDAS study sample ... 19
Table S2: Baseline characteristics of DEDEMAS-DEMDAS stratified by sex 21
Table S3: Loss to follow-up and death by study centre 23
Table S4: Reasons for death and loss to follow-up during 5 years of follow-up 23
Table S5: Baseline characteristics of stroke survivors who dropped out of the study due to death or loss to follow-up and those who did not 24
Table S6: Baseline characteristics of stroke survivors with early- versus delayed-onset PSD 26
Table S7: Risk factors for post-stroke dementia diagnosed before and after 6 months . 28
Table S8: Subgroup analysis stratifying the main analyses by sex 30
Table S9: Stroke recurrence during 5 years of follow-up 32
Table S10: Sensitivity analysis additionally adjusting the main analyses for acute stroke treatment 33
Table S11: Sensitivity analysis additionally adjusting the analysis split by post-stroke time period for age, sex, education, NIHSS, and acute stroke treatment 35
Table S12: Sensitivity analysis additionally adjusting the main analyses for stroke recurrence 37
Table S13: Sensitivity analysis adjusting the analysis split by post-stroke time period for age, sex, education, NIHSS, and stroke recurrence 39
Table S14: Sensitivity analysis additionally adjusting the 5-year PSD analyses for acute phase cognitive impairment 41
Table S15: Sensitivity analysis adjusting the analysis split by post-stroke time period for age, sex, education, NIHSS, and acute phase cognitive impairment 43
Table S16: Sensitivity analysis using the cut-off of 12 months for early- vs. delayed-onset dementia 45
Table S17: Sensitivity analysis with multiple imputation of the dementia onset date ... 47
Table S18: Comparison of subdistribution and cause-specific hazard ratios for 5-year PSD risk 49
Table S19: Time-dependent hazard ratios for PSD for selected risk factors 51

Table S20: Risk factors associated with PSD and PSCI showing unadjusted hazard ratios and odds ratios 52

Table S21: STROBE checklist for the reporting of observational studies in epidemiology 54

Table S22: The banner list of DEMDAS investigators... 55

References 62

Supplementary Methods

DEMDAS study centres

DEMDAS was conducted at seven tertiary stroke centres located in major German cities: the interdisciplinary stroke center including the Institute for Stroke and Dementia Research (coordinating institution) and the Department of Neurology, University Hospital, LMU Munich; the Department of Neurology, Klinikum rechts der Isar, School of Medicine, Technical University of Munich; the Division of Vascular Neurology, Department of Neurology, University Hospital Bonn; the University Medical Center, the Department of Neurology, Göttingen; and the Department of Neurology and Institute of Cognitive Neurology and Dementia Research, Otto von Guericke University Magdeburg; the Center for Stroke Research Berlin and the Department of Neurology of the Charité - Universitätsmedizin Berlin. These centres operate within larger regional networks of stroke care and typically serve a diverse mix of patients, including both direct admissions and transfers requiring advanced treatment.

Pathways of patient admission to the centres

- **Direct presentation**: Some patients self-presented to emergency departments.
- **EMS referral**: Others were transported directly by emergency medical services based on regional triage protocols, treatment indication, and hospital capacities.
- **Inter-hospital transfers**: Patients initially admitted to smaller or non-specialized hospitals were often transferred to tertiary centers when specialized treatment such as mechanical thrombectomy was indicated.

The proportion of patients entering through each pathway varied by location. For example, the Magdeburg site, being the only comprehensive stroke centre in a large catchment area, admitted patients from a wider geographic range. In contrast, metropolitan areas like Berlin, Munich, or Bonn have multiple tertiary centres, which may lead to a more diverse mix of local and referred patients, including both nearby residents and patients transferred from outside the immediate city due to the centres' specialized expertise or capacity.
Regardless of referral pathway, all participants were treated in dedicated stroke units within the participating hospitals, ensuring standardized acute management and study enrollment procedures.

Baseline assessments

At enrolment, a comprehensive interview and assessments were conducted using standardised protocols. Data collection included sociodemographic information, family history, medical history of previous diagnoses, medication use, and vascular risk factors. Participants were counted as having a cardiovascular risk factor (hypertension, dyslipidaemia, diabetes mellitus, atrial fibrillation, prior stroke, or ischaemic heart disease) when they had ever received a respective diagnosis (before or at the time of hospitalisation for the stroke after which they were recruited into DEDEMAS/DEMDAS), representing a history of or currently having that risk factor. Diabetes mellitus included both type 1 and type 2 diabetes. Clinical evaluations included physiological measurements (e.g. blood pressure and BMI measurement) and scales such as the National Institutes of Health Stroke Scale (NIHSS), Modified Rankin Scale (mRS), Glasgow Coma Scale (GCS). For cognitive testing, the Mini Mental State Examination (MMSE) and Montreal Cognitive Assessment (MoCA) were applied. Peripheral blood samples were collected from all patients and biochemical assessments were performed as part of the clinical routine. Ischaemic stroke subtyping was performed according to the Trial of Org 10172 in Acute Stroke Treatment (TOAST)[1] classification by trained neurologists at each of the participating centres.

Biochemical assessments and biobanking

Peripheral blood assessments included complete blood count, LDL-, HDL-, and total cholesterol, triglycerides, fasting glucose, glycated haemoglobin A1c, electrolytes, transaminases, creatinine, high-sensitivity C-reactive protein, fibrinogen, procalcitonin, homocysteine, thyroid hormones, vitamin B12, folate, total and MB-creatinine kinase, troponin T, and routine coagulation markers. Additional blood samples were collected for biobanking (serum, plasma, DNA, and miRNA) at baseline and follow-ups according to standard operating procedures. All samples were centralised in the coordinating centre in Munich, where they were double-pseudonymised and managed via a secure data integration system (DIS) developed by the Munich Biotech Cluster m4 with maintenance and support by Bitcare GmbH. Data integrity was ensured by independent verification by two blinded data managers.

Genetic ancestry analysis

To confirm the continental-level genetic ancestry of the study cohort and perform quality control, participant genotype data (genome build hg19) was compared against the 1000 Genomes Project (1kG) Phase 3 reference panel (N=2504 samples, hg19). All analyses were conducted using PLINK (v1.9) and R (v4.4.3]).

Prior to merging, both the study cohort data and the 1kG reference panel underwent quality control using PLINK. Filters included removal of non-autosomal SNPs, SNPs with minor allele frequency < 0.01, SNP call rate < 95%, sample call rate < 95%, and SNPs significantly deviating from Hardy-Weinberg equilibrium ($p < 1x10^{-6}$). The QC'd datasets were then harmonized based on genomic position (hg19). Allele consistency (A1/A2) and potential strand issues were checked during merging with PLINK; SNPs with irresolvable mismatches were excluded. The harmonized and merged dataset was subsequently LD-pruned using PLINK (--indep-pairwise 50 5 0.2), removing SNPs in high linkage disequilibrium ($r^2 > 0.2$) within a 50kb window, stepping 5 SNPs at a time. Principal Component Analysis (PCA) was performed on the final merged, QC'd, and pruned dataset using the --pca function in PLINK, calculating the top 20 principal components (PCs). The first two PCs were visualized using ggplot2 in R (Figure S2), with samples colored by their 1kG super-population label (AFR, AMR, EAS, EUR, SAS) or study cohort membership, allowing for visual inspection of ancestry clustering.
To quantitatively assess ancestry, a Random Forest classifier was built using the randomForest R package. The model was trained using the first 10 PCs of the 1kG reference samples as predictor variables and their known super-population labels as the outcome. This trained model was then applied to the principal components of the study cohort samples to predict their probabilities of belonging to each of the five 1kG super-populations. The results were used to confirm the expected European ancestry of the study cohort.

Definition of criteria for metabolic syndrome
Metabolic syndrome was defined as the presence of 3 or more of the following 5 criteria at baseline, as defined by Alberti et al.[2]

1. *Elevated waist circumference:* ≥102 cm in males or ≥88 cm in females
2. *Elevated triglyceride levels:* ≥150 mg/dL or current pharmacotherapeutic treatment for elevated triglycerides
3. *Reduced HDL-C levels:* <40 in males or <50 in females or current pharmacotherapeutic treatment for low HDL-C
4. *Elevated blood pressure:* Systolic ≥130 and/or diastolic ≥85 mm Hg or current pharmacotherapeutic treatment for hypertension
5. *Elevated blood glucose:* HbA_{1c} ≥5·7 or current pharmacotherapeutic treatment for elevated glucose

Brain MRI acquisition
Patients underwent cranial MRI examinations at baseline within three days (DEDEMAS) or five days (DEMDAS) of stroke onset. All examinations were scanned on 3-Tesla systems (Siemens Healthineers, Erlangen, Germany). The following imaging sequences were acquired: 3D T1- weighted (T1w) magnetisation prepared rapid gradient echo (MPRAGE), 3D fluid-attenuated inversion recovery (FLAIR), diffusion-weighted imaging (DWI) with multiple diffusion directions, T2-weighted (T2w) turbo spin echo, and T2*-weighted (T2*w) fast low angle shot (FLASH) gradient echo. The protocols used per sequence have been described in detail previously.[3] There were differences between the imaging protocols used for the run-in phase study (DEDEMAS) and the multicentre DEMDAS study. These differences are minor (not relevant for analyses) due to differences in scanner hardware and software across sites with the exception of the first 18 patients that were recruited in DEDEMAS, who were scanned with a different protocol.[3] There were no major imaging protocol deviations, which led to an exclusion of one or more image series.

Brain volume and primary infarct volume
Normalised brain volume was defined as (brain volume + infarct volume) / total intracranial volume. Normalised infarct volume was defined as infarct volume / total intracranial volume.[3]

Assessment of small vessel disease markers
Conventional SVD markers on baseline MRI were assessed semi-quantitatively using widely accepted consensus criteria.[4,5] The following individual SVD markers were assessed: lacunes, white matter hyperintensities, cerebral microbleeds, and perivascular spaces. In earlier work, the assessment of these markers has been described in detail.[3] Normalised WMH volume was defined as WMH volume / total intracranial volume.

Assessment of diffusion MRI data
Microstructural tissue integrity was assessed using mean skeletonised mean diffusivity (MSMD) based on a single-shell diffusion-weighted imaging sequence. Diffusion MRI data were visually assessed and preprocessed including denoising, Gibbs artefact removal, and correction for head motion and eddy current-induced distortions. This was done using tools from MRtrix3 (mrtrix.org/, 'dwidenoise', 'mrdegibbs') and the Functional Magnetic Resonance Imaging of the Brain Software Library (FSL; version 5.0.11, 'eddy_correct'). To compute MSMD, we then employed a tract-based spatial statistics pipeline on DTI maps and a custom white matter skeleton mask, as done previously.[6,7]

Follow-up assessments

Participants and their informants were invited for in-person follow-up visits at 6, 12, 36, and 60 months post-stroke, during which they underwent comprehensive cognitive and functional assessments conducted by trained neuropsychologists, study nurses, and physicians. Additionally, telephone interviews were conducted at 3, 24, and 48 months post-stroke to collect clinical and cognitive data. A detailed battery of neuropsychological tests, covering five cognitive domains (executive function, memory, language, attention, and visuospatial function), and functional tests, including the modified Rankin Scale (mRS), Barthel Index (BI), and Instrumental Activities of Daily Living (IADL), were administered during in-person follow-ups. Standardised questionnaires were used to document new clinical events, medical treatments, and cardiovascular risk factors at the follow-ups.

To minimize attrition and missing data, a standardised protocol was followed to contact participants or their informants for follow-ups.[3] Initially, a trained study nurse contacted participants by telephone prior to each follow-up timepoint to schedule an in-person visit. If participants could not be reached by telephone, the nurse called their informants. In cases where neither the participant nor the informant could be reached, an invitation for an in-person visit was sent by mail. If there was still no response, the data manager contacted the local registration office to confirm whether the participant was alive or had changed addresses. If a new address was obtained, the steps were repeated to establish contact.

For participants who could be reached but were unable or unwilling to attend in-person visits, two alternative options were provided: first, they were offered the opportunity to complete portions of the study questionnaires via telephone interviews with study nurses. If this was not feasible, they were mailed the questionnaires with a request to complete and return them to the study site.

Cognitive follow-up assessments

At the in-person follow-up visits at 6, 12, 36, and 60 months post-stroke cognitive performance was assessed in five domains via a detailed neuropsychological test battery:

1. *Executive function*
   - "Trail Making Test Part B" from the "Consortium to Establish a Registry for Alzheimer's Disease Plus (CERAD-Plus)[8]" battery
   - "Stroop Colour-Word-Interference Test"[9]
2. *Memory*
   - "Word List Learning/Recall and Recognition" and "Figure Recall" from CERAD-Plus[8]
   - immediate and delayed recall of the "Rey-Osterrieth Complex Figure (ROCF)"[10]
3. *Language*
   - "Semantic and Phonemic Fluency" and "Boston Naming Test" from CERAD-Plus[8]
4. *Attention*
   - "Trail Making Test Part A" from CERAD-Plus[8]
   - "Digit-Symbol-Substitution Test of the Wechsler Intelligence Scale"[11]
5. *Visuospatial function*
   - "Figure Drawing Test" from CERAD-Plus[8]
   - copy test of ROCF[10]

We calculated test-specific z-scores based on published norms: (1) Z-scores of the CERAD test battery were based on published norms using a standardised program.[12] (2) Z-scores of Rey-Osterrieth complex figure-copy, immediate and delayed recall were calculated based on published norms corrected for age, sex, and education.[13] (3) Z-scores of the Stroop test were calculated based on published norms corrected for age, sex, and education.[14] (4) Z-scores of the number symbol test were calculated based on normative scores of the Wechsler Adult Intelligence Scale, Third Edition (WAID-III).[15]

Furthermore, the "Clinical Dementia Rating Scale (CDR)[16]" was completed by both the study participant and their informant to assess dementia severity at each in-person follow-up visit. Short screening tests (Mini-Mental State Examination (MMSE) and Montreal Cognitive Assessment (MoCA)) were repeatedly applied at baseline and in-person follow-up visits. The modified German version of the "Telephone Interview for Cognitive Status" (TICS) and a telephone version of the MoCA[17] were applied at the telephone interviews at 3, 24, and 48 months. All tests were performed and rated by centrally trained investigators.

If patients were unable or not willing to undergo the comprehensive neuropsychological test battery, the following hierarchical procedure to reduce the volume of cognitive testing was applied to minimize attrition and missing data:

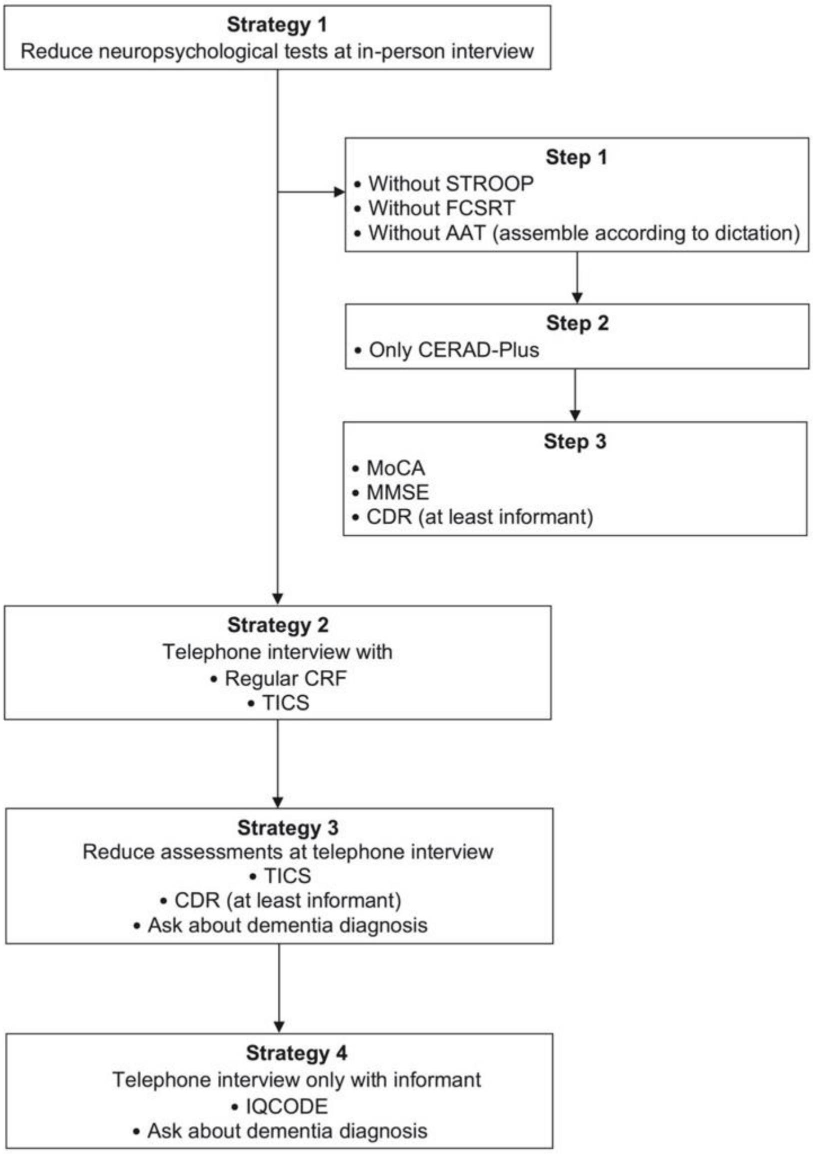
Strategy 1
Reduce neuropsychological tests at in-person interview
Step 1
• Without STROOP
• Without FCSRT
• Without AAT (assemble according to dictation)
Step 2
• Only CERAD-Plus
Step 3
• MoCA
• MMSE
• CDR (at least informant)
Strategy 2
Telephone interview with
• Regular CRF
• TICS
Strategy 3
Reduce assessments at telephone interview
• TICS
• CDR (at least informant)
• Ask about dementia diagnosis
Strategy 4
Telephone interview only with informant
• IQCODE
• Ask about dementia diagnosis

Step-by-step process of endpoint assessment

1. Identification of patients with cognitive impairment

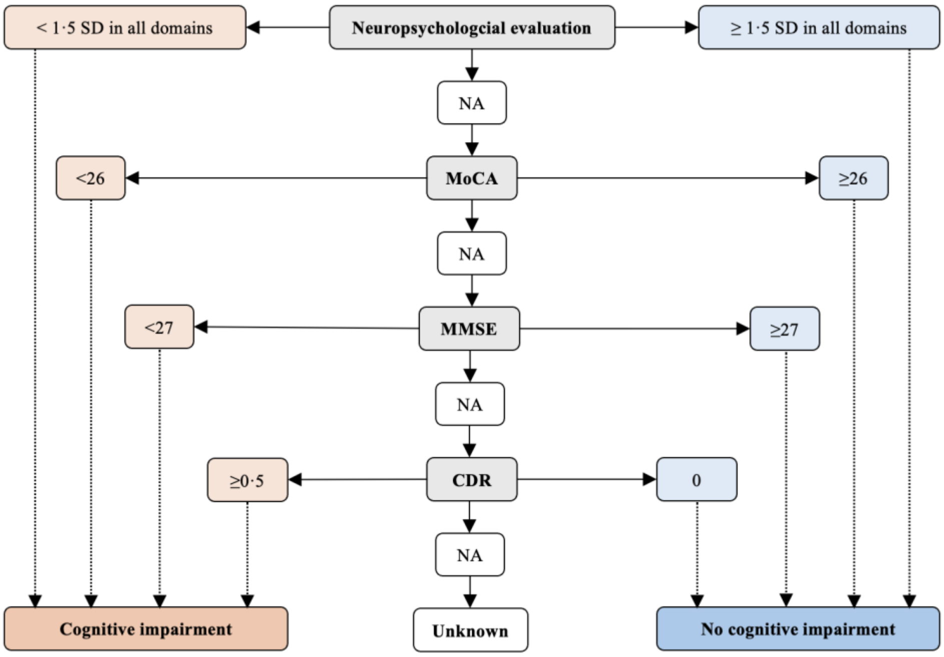

2. Screening for functional impairment → isolated MCI or MCI + functional impairment

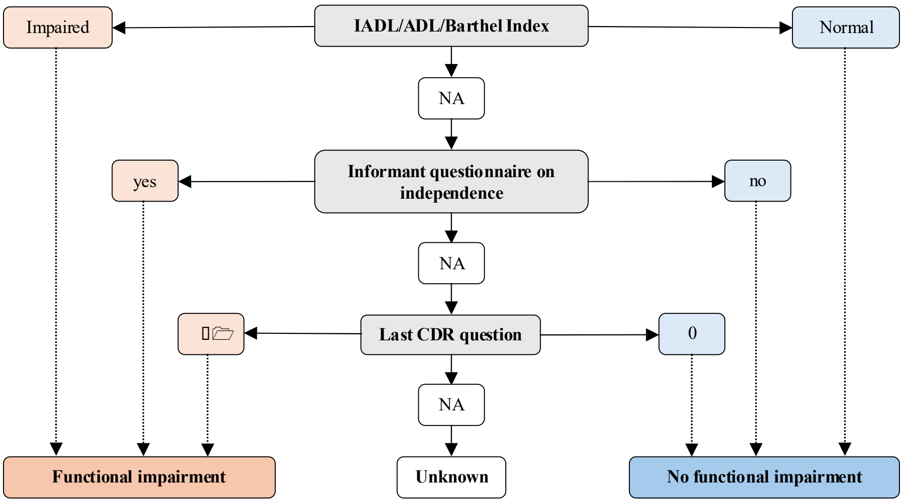

3. Consensus committee meeting assessing the DSM-V criteria → final differential diagnosis: MCI vs. dementia
    - Ruling out depression (in psychiatric assessment or CES-D ≥ 16) and delirium (physical examination or DRS > 15)
    - Determination if functional impairment is the result of cognitive decline
    - Definition of date of dementia diagnosis
    - Screening of medical notes of patients that had only home visits
    - Screening of medical files of all patients who died or were lost to follow-up

Loss to follow-up

Participants were considered lost to follow-up if they revoked consent to participate in the study or could not be contacted after multiple attempts via telephone, mail, or their informant. At study entry, participants provided consent for the investigators to access medical records and retrieve mortality data from the resident registration office. However, if consent was later revoked, obtaining this information was legally prohibited.

Data management and quality control

Demographic, clinical, and neuropsychological data from both baseline and follow-up visits, as well as telephone interviews, were initially collected by participating study sites using Case Report Forms (CRFs) specifically designed for this study. Completed CRFs were then sent to the coordinating center at the Institute for Stroke and Dementia Research (ISD), LMU Munich. Trained data managers conducted comprehensive quality control procedures. As a first step, each CRF was manually reviewed for completeness and screened for potential outliers and implausible values. Any discrepancies or missing data were resolved by contacting the study nurses at the respective sites. Data from the CRFs were subsequently digitised into a central database using TeleForm (Electric Paper GmbH, Lüneburg, Germany). Centralised plausibility checks were performed regularly using standardised algorithms to identify outliers or implausible entries. Whenever issues were detected, study nurses at the corresponding sites were consulted to verify and correct the data. Data management and central quality control were conducted using SAS version 9·4 (SAS Institute Inc., Cary, NC). Details of MRI image quality control have been reported previously.[3]

Supplementary statistical methods
Handling of quantitative variables

	**Sociodemographic variables**	**Clinical/cognitive acute phase deficits**	**Vascular and metabolic risk factors**	**Neuroimaging parameters**	**Pre-stroke cognition/function**
**Continuous variables**	age, educational attainment	NIHSS score, Barthel Index, Delirium Rating Scale score, MoCA score	BMI, systolic blood pressure, diastolic blood pressure, HbA_{1c}, LDL-C, HDL-C, triglycerides, count of metabolic syndrome components	normalised brain volume, normalised infarct volume, SVD score, lacune count, normalised WMH volume, CMB count, perivascular space grade, mean diffusivity	Modified Rankin Scale score, IQCODE score
**Categorical variables**	age: (a) tertiles [≤65 *vs* 66-73 *vs* ≥74], (b) dichotomous [<74 *vs* ≥74], educational attainment: dichotomous [≤12 *vs* >12]	NIHSS score: dichotomous [0-2 *vs* ≥3], acute phase cognitive impairment: dichotomous [MoCA <26 or MMSE <27 *vs* MoCA ≥26 or MMSE ≥27		lacune count: dichotomous [<3 *vs* ≥3]	

Age tertiles were determined based on the study sample. Age ≥74 represents the highest age tertile in our sample and is similar to previously used categorisation.[18] Educational attainment was categorised using a pre-defined and previously reported cut-off.[18-20] The admission NIHSS score cut-off was identified within the study sample using the maxstat.test function from the "maxstat" package in R, which determines the optimal cut point for separating groups based on the survival outcome.[21] An admission NIHSS ≥3, identified as the optimal cut-off, aligns with the "major stroke" definition used in the OxVasc study.[18] The lacune count cut-off was selected based on earlier reports.[22-24]

Primary outcome
Post-stroke dementia

Since death is a competing risk for PSD, we calculated the 5-year cumulative incidence of PSD using a Kaplan-Meier-estimator, adjusted for the competing risk of death.[25] Differences in cumulative incidence between subgroups of risk factors were evaluated using Gray's test.[26] Associations between baseline risk factors and 5-year PSD risk were assessed using cause-specific and Fine-Gray subdistribution Cox proportional hazard models, accounting for the competing risk of death.[27] The proportional hazards (PH) assumption was tested using the Grambsch and Therneau test based on Schoenfeld residuals and reported in Table S18. In cases where the PH assumption was violated, we used flexible parametric survival models with natural splines.[28] Risk factors were

selected based on previous (conflicting) evidence regarding their association with PSD and PSCI[18,20,29-32] or with dementia in non-stroke populations.[33,34]All multivariate Cox regression models included the covariables age, sex, education, and admission NIHSS score, based on previous evidence on the importance of these risk factors.[18,20]

Secondary outcomes

Early-onset and delayed-onset PSD

To explore the relationships between baseline risk factors and early-onset PSD (dementia diagnosed 3-6 months post-stroke) and delayed-onset PSD (diagnosis >6 months post-stroke), we used the "survSplit" function in R's "survival" package to divide the follow-up period into two discrete intervals: an early phase (≤6 months) and a later phase (>6 months).[25,32] The first part of the model assessed the relationship between baseline risk factors and early-onset PSD, while the second part evaluated their association with delayed-onset PSD. For the second part, individuals with early-onset PSD or those censored before 6 months post-stroke were excluded.[22,23] This effectively reset the 6-months mark as an arbitrary "new" T0, disregarding events that occurred prior.
We set the cut-point at 195 days (approximately 6·4 months) to account for the fact that most individuals completed their 6-month follow-up slightly later (median 6·2 [IQR 5·9-6·7]) than the exact 183-day mark. Sensitivity analyses applying earlier or later cut points yielded consistent results. Overall, 706 patients contributed to the analysis for early-onset PSD, and 617 of these also contributed to the analysis for delayed-onset PSD.

Following the Cox regression analysis, we calculated population attributable fractions (PAFs) for relevant binary risk factors for early-onset and delayed-onset PSD. PAFs represent the proportion of dementia cases in our study population that could theoretically have been prevented if the specified risk factor had been absent or eliminated. For each binary risk factor, PAFs and their 95% confidence intervals were calculated for 10,000 bootstrap resamples using the baseline prevalence of the risk factor in the respective subsample (706 and 617 patients for early- and delayed-onset PSD, respectively) and the adjusted HRs derived from multivariable Cox proportional hazards models adjusted for age, sex, education, admission NIHSS, and stroke recurrence. The difference in PAFs between the two periods was computed for each bootstrap iteration. The 2.5th and 97.5th percentiles of the bootstrap distribution of PAF differences were used to derive the 95% confidence interval.

Post-stroke cognitive impairment

Associations between baseline risk factors and post-stroke cognitive impairment (PSCI) were assessed over 60 months post-stroke as a combination of mild-cognitive impairment and dementia at the 6-, 12-, 36-, and 60-month follow-ups. To account for repeated measurements within individuals, we employed generalised estimating equations (GEEs) with a first-order autoregressive working correlation structure and robust SEs. GEE models were adjusted for age, sex, education, and admission NIHSS score.

False Discovery Rate (FDR) Correction

To account for multiple comparisons in the main analyses, we applied FDR correction using the Benjamini-Hochberg procedure across all tested risk factors for each outcome (PSD and PSCI). Specifically, we corrected for 48 statistical comparisons for PSD and 47 for PSCI, including cases where a single risk factor was represented as both a continuous and categorical variable.

Sensitivity and subgroup analyses

Subgroup analyses stratified the Cox regression models examining baseline risk factors for PSD by sex.
Sensitivity analyses extended the Cox models for 5-year PSD risk, early-onset, and delayed-onset PSD by including the additional covariates acute stroke treatment, recurrent stroke, and acute phase cognitive impairment. Recurrent stroke introduces a time-dependent risk for PSD, as it constitutes a separate time-to-event outcome. Therefore, for sensitivity analyses incorporating recurrent stroke, we divided the follow-up period for patients who experienced a recurrent stroke without a prior dementia diagnosis into two phases: pre-recurrence and post-recurrence.[25] This approach accounted for different PSD risk before and after the recurrence, resulting in a larger sample size for the sensitivity analysis. Additionally, the analysis for early- versus delayed-onset PSD was conducted with a 12-month instead of the original 6-moth cut-off.

Missing data

Baseline variables generally had missing value rates below 10%, except for acute phase MoCA score, normalised brain volume, normalised infarct volume, normalised WMH volume, and APOE genotype (**Table S1**). Data for the model covariates age, sex, education, and admission NIHSS score were complete. Missingness was assumed to be at random, and the main analyses were conducted after excluding patients with missing values for the main independent variable.

Power calculation
A priori power calculations for survival analysis estimated that a sample size of 600 would enable us to detect associations between risk factors (10% exposure rate) and PSD with a HR of 2·0 with a power of 91%.[19]

Post-hoc, we calculated the minimum detectable effect size (HR) for different risk factor prevalence rates for binary variables and for different variances for continuous variables (per SD), respectively. The graphs and tables below illustrate the effect sizes detectable with 80% power and a 5% significance level, given the final sample size of 706 patients contributing to survival analysis and the cumulative 5-year PSD incidence of 8·3%. This observed incidence was lower than initially anticipated in the a priori power calculation.

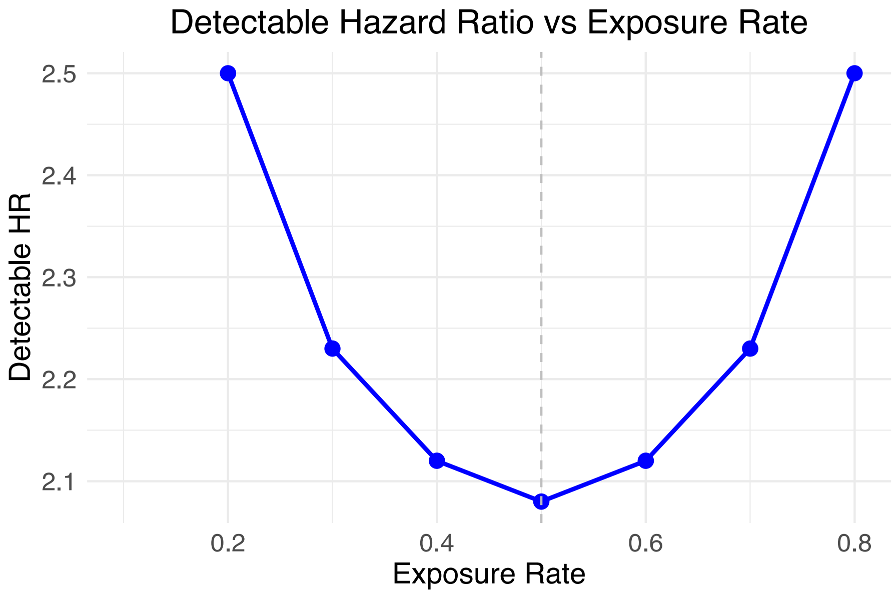

Risk factor prevalence	10%	20%	30%	40%	50%	60%	70%	80%
**Detectable hazard ratio**	NA	2·50	2·23	2·12	2·08	2·12	2·23	2·50

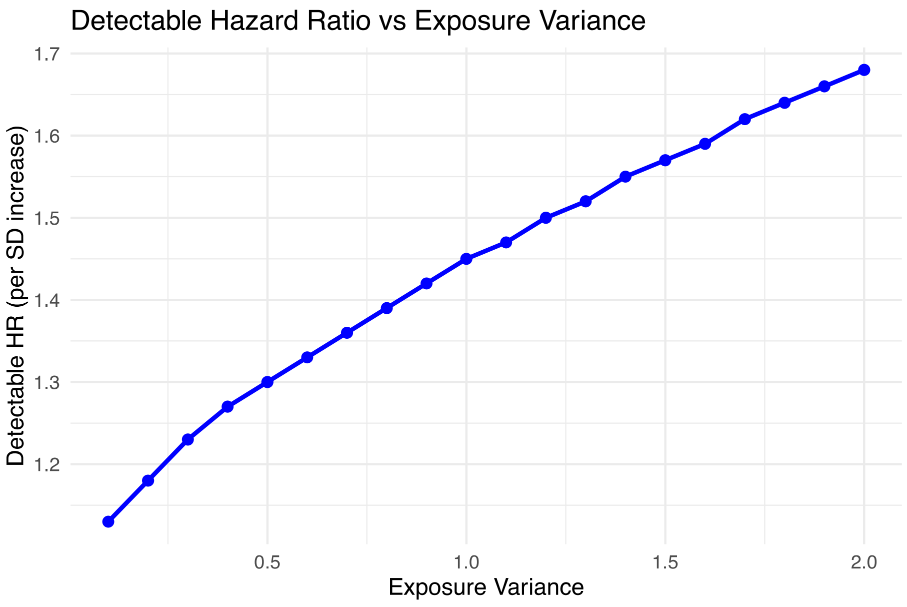

Risk factor variance	0·25	0·5	0·75	1·0	1·25	1·5	1·75	2·0
**Detectable hazard ratio (per SD increase)**	1·21	1·30	1·38	1·45	1·51	1·57	1·63	1·68

Figure S1: Distribution of time since stroke for each follow-up time point

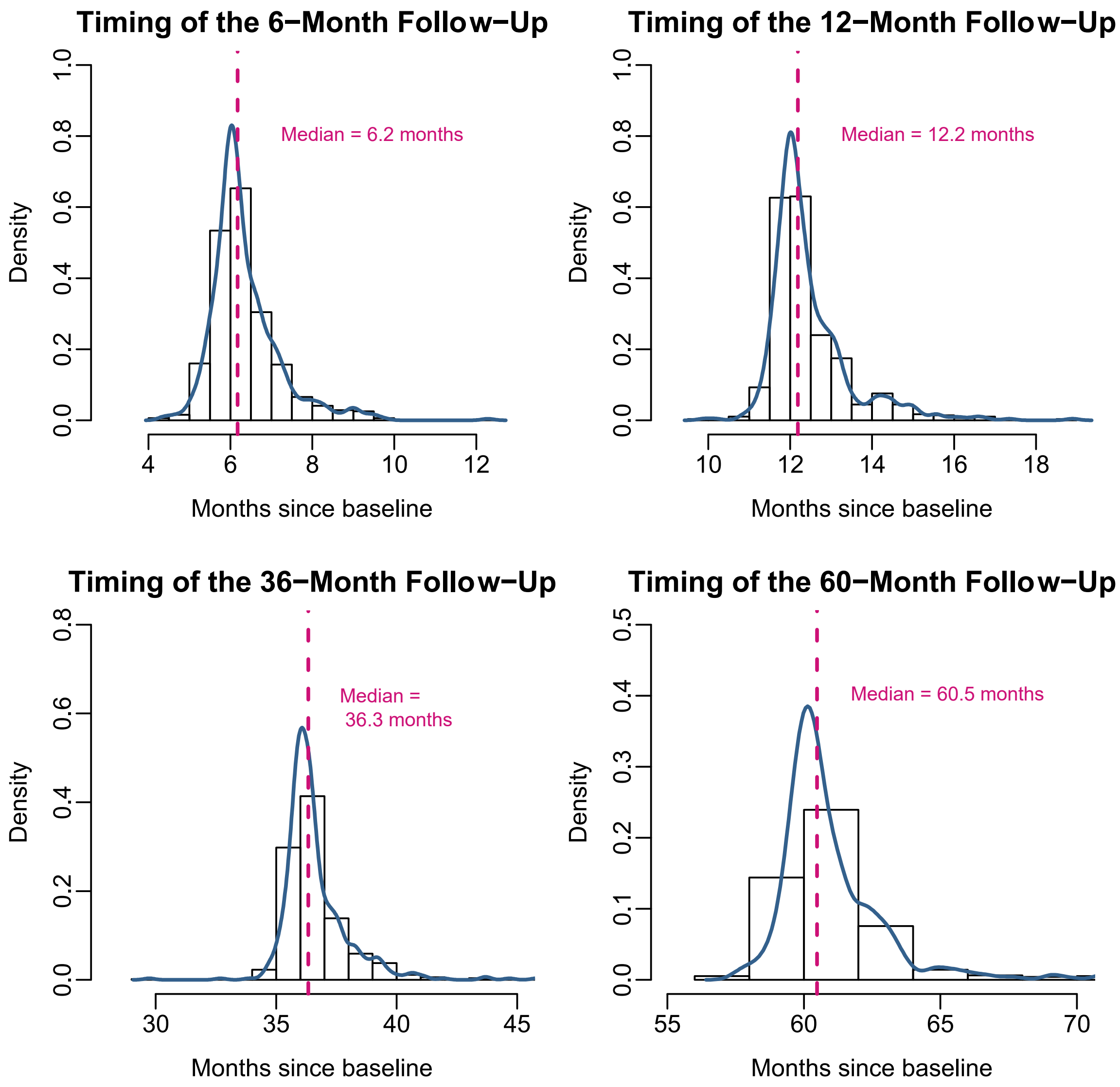

Distribution and density curve of time since baseline for each of the distinct in-person follow-up time points including the respective study sample medians (vertical dashed lines). Exact data on time since stroke were available for 637 participants at 6 months, for 584 at 12 months, for 527 at 36 months, and for 489 at 60 months.

Figure S2: Genetic ancestry of the study cohort confirmed by PCA with 1000 genomes

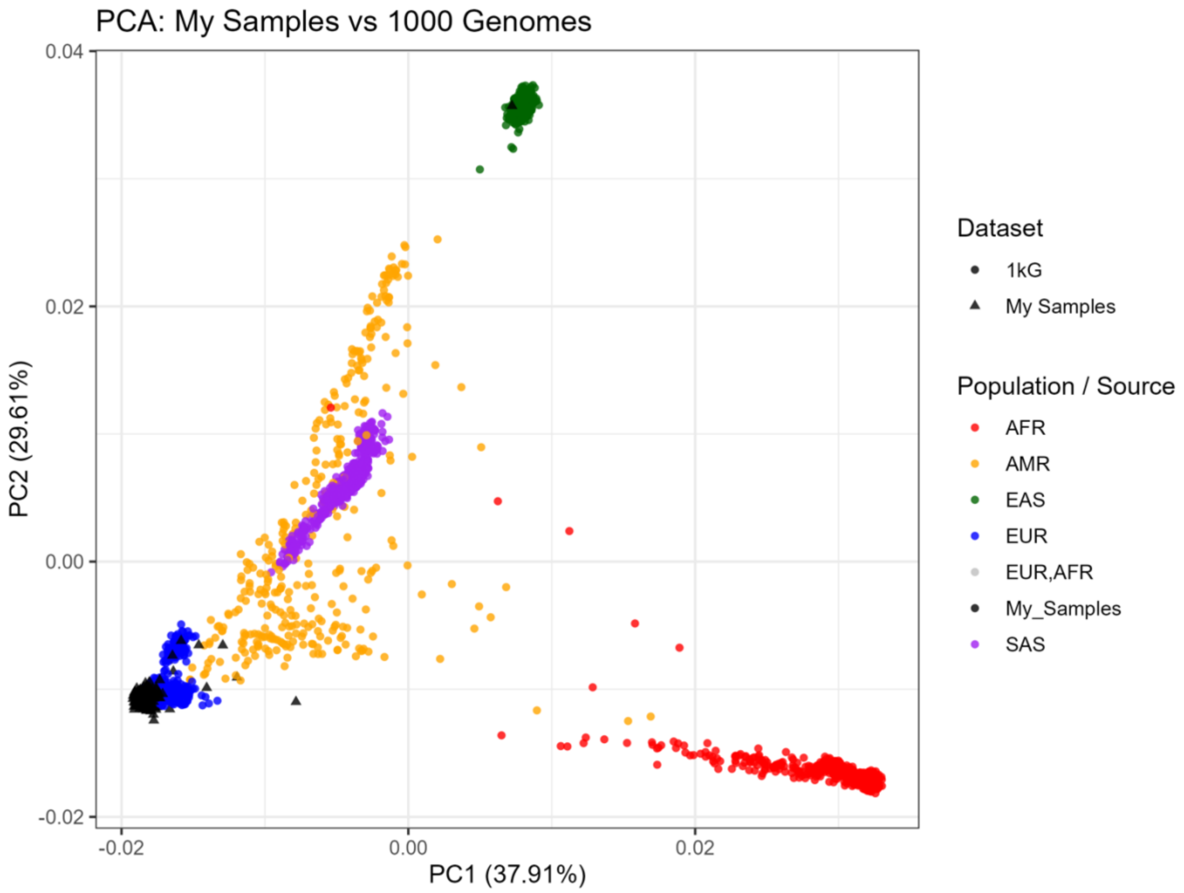

Principal Component Analysis (PCA) of the DEDEMAS/DEMDAS study cohort (n=599) and 1000 Genomes Project (1kG) Phase 3 reference panel (N=2,504) to assess continental-level ancestry. PCA was performed using pruned, quality-controlled genotype data (hg19) with the top two PCs plotted. Each point represents an individual from either the 1kG panel (colored by super-population) or the study cohort (black triangles). The study samples cluster closely with the European (EUR) reference group, confirming the cohort's expected genetic ancestry. PCA was conducted in PLINK v1.9 and visualized in R v4.4.3 using ggplot2.

Figure S3: Cumulative incidence curve for dementia over 5 years post stroke

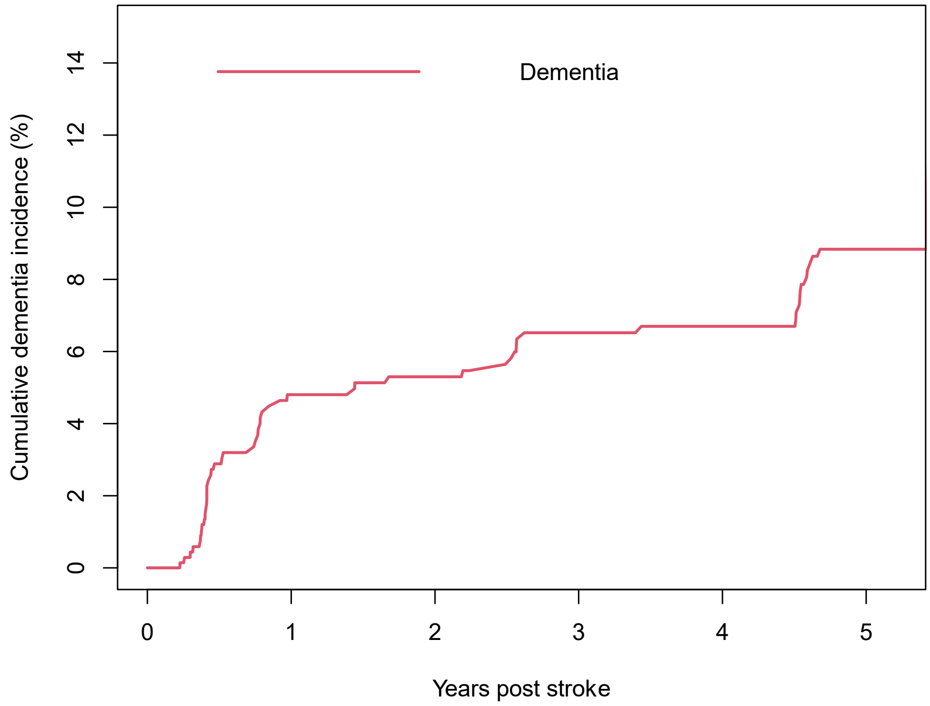

**Number at risk**	706	591	558	527	500	342

Cumulative Kaplan-Meier curve of dementia incidence during five years after stroke in the total DEDEMAS-DEMDAS cohort (N=706).

Figure S4: Cumulative incidence for dementia and death over 5 years post stroke

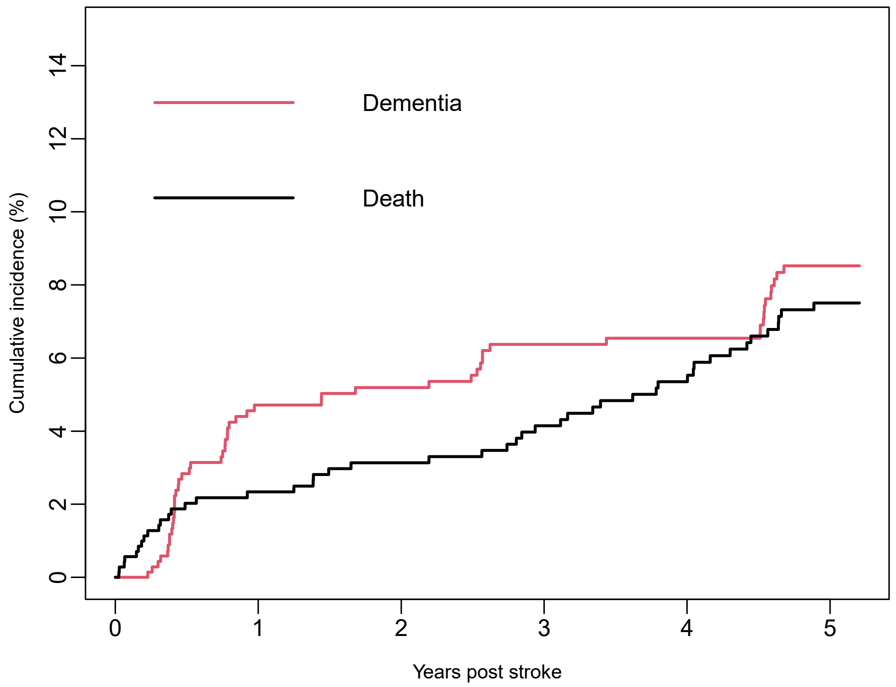

Number at risk	706	591	558	527	501	343

Competing risks cumulative incidence for dementia and death during five years after stroke.

Figure S5: Cumulative incidence curve for stroke recurrence over 5 years post stroke

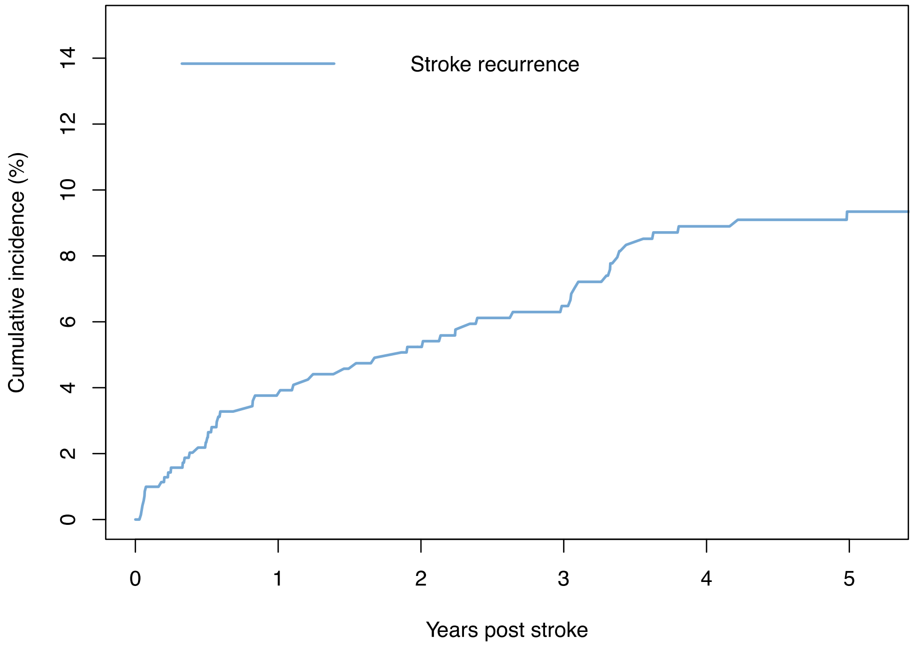

Number at risk	706	595	548	515	484	370

Kaplan-Meier cumulative incidence curve for stroke recurrence during five years after stroke.

Figure S6: Proportion of endpoints per follow-up time point

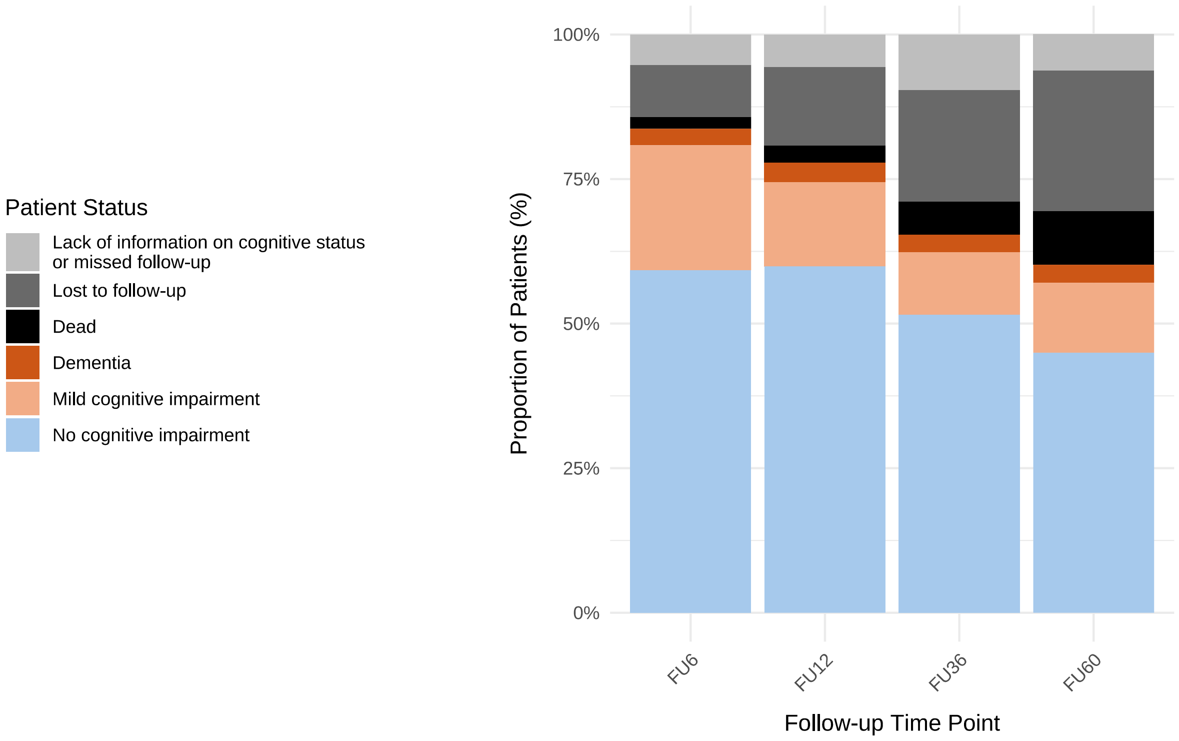

Proportion of the initial 736 patients at each in-person follow-up who had no cognitive impairment, mild cognitive impairment, or dementia; who were dead or lost to follow-up; or for who information was missing because they missed follow-up assessment or information on cognitive status were insufficient. At the 6-, 12-, 36-, and 60-month follow-up, 638, 585, 528, and 494 patients were assessed, respectively.

Figure S7: Time-varying associations with PSD risk

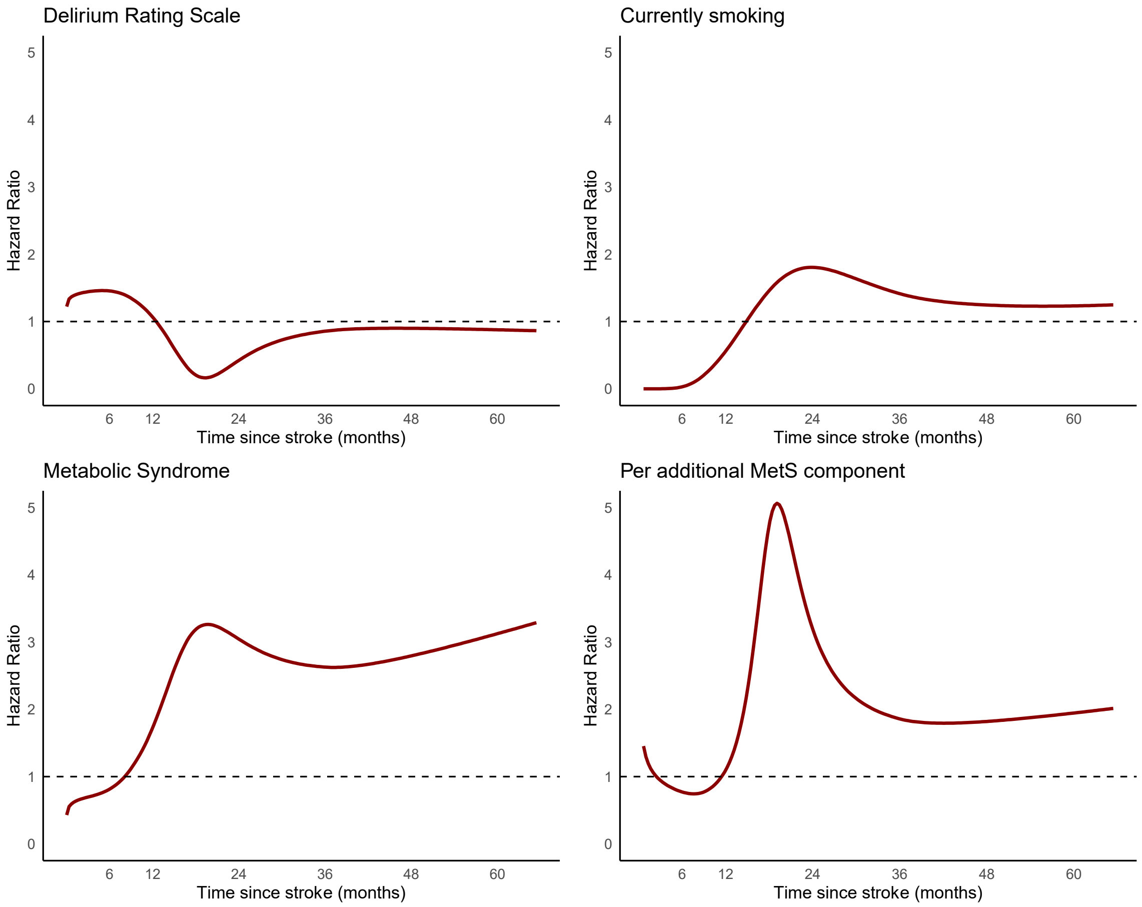

Time-varying hazard ratios for post-stroke dementia (PSD) by Delirium Rating Scale score (top left), currently smoking at the time of stroke (top right), Metabolic Syndrome (bottom left), and per additional MetS component (bottom right). Each panel displays the estimated hazard ratio (HR, solid line) over time since stroke, with 95% confidence intervals (shaded area), based on flexible parametric survival models[28] allowing for time-varying effects (detailed in **Table S19**). While Delirium Rating Scale score, Metabolic Syndrome, and count of MetS components show significant time-dependent associations with PSD risk, smoking showed no significant association at any time point. Models were adjusted for age, sex, education, and admission NIHSS. MetS=Metabolic Syndrome. NIHSS=National Institutes of Health Stroke Scale.

Table S1: Baseline characteristics of the entire DEDEMAS-DEMDAS study sample

	Total sample (N = 736)	Missing data
**Study**		
DEDEMAS[a]	136 (18·5%)	··
DEMDAS	600 (81·5%)	··
Munich-LMU[a]	219 (29·7%)	··
Munich-TUM[b]	69 (9·4%)	··
Berlin-1[c]	33 (4·5%)	··
Berlin-2[d]	38 (5·2%)	··
Bonn[e]	105 (14·3%)	··
Göttingen[f]	81 (11·0%)	··
Magdeburg[g]	55 (7·5%)	··
**Sociodemographic factors**		
Age (years)	68·0 (11·2)	0 (0%)
Age ≥ 74 years	261 (35·5%)	0 (0%)
Female sex	245 (33·3%)	0 (0%)
Education (years)	13 (12-16)	0 (0%)
Education ≤ 12 years	292 (39·7%)	0 (0%)
**Pre-stroke employment status**		5 (0·7%)
Working full-time	185 (25·3%)	··
Working part-time	53 (7·2%)	··
On sick leave	4 (0·5%)	··
Retired	479 (65·5%)	··
Unemployed (seeking employment)	10 (1·4%)	··
**Pre-stroke living situation***[1]		0 (0%)
Private household, living alone	191 (25·9%)	··
Private household, with spouse/life partner	522 (70·9%)	··
Private household, with children	56 (7·6%)	··
Private household, with other person/s	6 (0·8%)	··
Retirement home	2 (0·3%)	··
**Genetic ancestry**		137 (18·6%)
European	597 (99·7%)	··
Ad Mixed American	1 (0·2%)	··
East Asian	1 (0·2%)	··
**Clinical/cognitive acute phase deficits**		
Admission NIHSS score	3 (1-5)	0 (0%)
Admission NIHSS ≥3	387 (52·6%)	0 (0%)
Barthel Index score	100 (80-100)	4 (0·5%)
Delirium Rating Scale score	0 (0-1)	0 (0%)
Acute phase MoCA score	25 (23-28)	89 (12·1%)
Acute phase cognitive impairment*[2]	382/709 (53·9%)	27 (3·7%)
**Cardiovascular risk factors**		
Hypertension	571 (77·6%)	0 (0%)
Diabetes mellitus	150 (20·4%)	0 (0%)
Dyslipidaemia	229 (31·1%)	0 (0%)
Current smoking	171 (23·2%)	0 (0%)
Regular alcohol consumption	557 (75·7%)	0 (0%)
Atrial fibrillation	148 (20·1%)	0 (0%)
Prior history of stroke	79 (10·7%)	0 (0%)
Ischaemic heart disease	80 (10·9%)	0 (0%)
BMI (kg/m^2)	27·0 (4·3)	1 (0·1%)
Systolic blood pressure (mmHg)	139 (129-150)	5 (0·7%)
Diastolic blood pressure (mmHg)	80 (71-86)	5 (0·7%)
HbA_{1c} (%)	5·7 (5·4-6·1)	50 (6·8%)
LDL cholesterol (mg/dL)	126 (103-154)	22 (3·0%)
HDL cholesterol (mg/dL)	48 (40-58)	27 (3·7%)
Triglycerides (mg/dL)	121 (91-170)	44 (6·0%)
**Metabolic syndrome components***[3]		
Abdominal obesity	391/689 (56·7%)	47 (6·4%)
Elevated triglycerides	233/692 (33·7%)	44 (6·0%)

Reduced HDL cholesterol	231/709 (32·6%)	27 (3·7%)
Elevated blood pressure	653/735 (88·8%)	1 (0·1%)
Prediabetes or diabetes mellitus	386/686 (56·3%)	50 (6·8%)
Metabolic syndrome (≥ 3 of the above components present)	365 (49·3%)	0 (0%)
**Index stroke classification**		
Ischaemic stroke	715 (97·1%)	0 (0%)
**TOAST classification of acute ischaemic stroke subtype**		0 (0%)
Large artery atherosclerosis	166 (22·6%)	··
Cardioembolism	164 (22·3%)	··
Small artery occlusion	86 (11·7%)	··
Other determined aetiology	29 (3·9%)	··
Undetermined aetiology	270 (36·7%)	··
Haemorrhagic stroke	21 (2·8%)	··
**Acute stroke treatment**		
Intravenous thrombolysis (IVT)	188 (25·5%)	0 (0%)
Endovascular thrombectomy (EVT)	78 (10·6%)	0 (0%)
IVT + EVT	57 (7·7%)	0 (0%)
Any reperfusion therapy (IVT and/or EVT)	209 (28·4%)	0 (0%)
**Neuroimaging parameters**		
Normalised brain volume (%)	67·8 (64·1-71·6)	79 (10·7%)
Stroke lesion volume (mm^3)	2288 (526-12408)	72 (9·8%)
Normalised infarct volume (%)	0·15 (0·03-0·78)	78 (10·6%)
Small vessel disease score		70 (9·5%)
0	259/666 (38·9%)	··
1	201/666 (30·2%)	··
2	136/666 (20·4%)	··
3	54/666 (8·1%)	··
4	16/666 (2·4%)	··
Lacune count	0 (0-0)	65 (8·8%)
≥3 lacunes	12 (1·8%)	65 (8·8%)
Normalised white matter hyperintensity volume (%)	0·22 (0·08-0·52)	78 (10·6%)
Cerebral microbleed count	0 (0-0)	70 (9·5%)
Perivascular space grade	1 (1-2)	66 (9·0%)
Mean skeletonised mean diffusivity (z-score)	-0·12 (-0·69-0·63)	108 (14·7%)
**Genetic risk factors**		
APOE genotype		142 (19·3%)
0 ε4 allele	463/594 (77·9%)	··
1 ε4 allele	122/594 (20·5%)	··
2 ε4 alleles	9/594 (1·5%)	··
**Pre-stroke clinical/cognitive function**		
Modified Rankin Scale score before stroke	0 (0-0)	0 (0%)
IQCODE score	48 (48-49)	60 (8·1%)

Data are n (%), median (IQR), mean (SD), or n/N (%). DEDEMAS (Determinants of Dementia After Stroke) represents the pilot phase of the DEMDAS study. APOE=apolipoprotein E. BMI=body-mass index. EVT=Endovascular thrombectomy. HbA_{1c}=glycated haemoglobin. HDL=high-density lipoprotein. IQCODE=Informant Questionnaire on Cognitive Decline in the Elderly. IVT=Intravenous thrombolysis. LDL=low-density lipoprotein. MoCA=Montreal Cognitive Assessment. NIHSS=National Institutes of Health Stroke Scale. TOAST=Trial of Org 10172 in Acute Stroke Treatment.

*[1] More than one can apply

*[2] MoCA <26 or mini-mental state examination <27 when MoCA was not available (n=73).

*[3] Defined according to Alberti et al.[2]

[a]Institute for Stroke and Dementia Research, Klinikum der Universität München

[b]Klinik für Neurologie, Klinikum rechts der Isar, Technische Universität München

[c]Center for Stroke Research Berlin, Department of Neuroloy – Campus Charité Mitte, Charité – Universitätsmedizin Berlin

[d]Center for Stroke Research Berlin, Department of Neurology – Campus Benjamin Franklin, Charité – Universitätsmedizin Berlin

[e]Klinik und Poliklinik für Neurologie, Universitätsklinikum Bonn

[f]Klinik für Neurologie, University Medical Center Göttingen

[g]Universitätsklinikum Magdeburg

Table S2: Baseline characteristics of DEDEMAS-DEMDAS stratified by sex

	Male (n = 491)	Female (n = 245)	P-value
**Sociodemographic variables**			
Age (years)	67·2±10·8	69·6±11·7	0·007
Age ≥74 years	159 (32·4%)	102 (41·6%)	0·02
Education (years)	14 (12-17)	12 (11-14)	<0·0001
Education ≤12 years	162 (33·0%)	130 (53·1%)	<0·0001
**Clinical/cognitive acute phase deficits**			
Admission NIHSS score	3 (1-5)	3 (1-5)	0·43
Admission NIHSS ≥3	252 (51·3%)	135 (55·1%)	0·37
Barthel Index score	100 (85-100)	100 (80-100)	0·30
Delirium rating scale score	0 (0-1)	0 (0-1)	0·70
Acute phase MoCA score	25 (22-27)	26 (23-28)	0·04
Acute phase cognitive impairment*[1]	273/476 (57·4%)	109/233 (46·8%)	0·01
**Cardiovascular risk factors**			
Hypertension	375 (76·4%)	196 (80·0%)	0·30
Diabetes mellitus	105 (21·4%)	45 (18·4%)	0·40
Dyslipidaemia	152 (31·0%)	77 (31·4%)	1·00
Current smoking	119 (24·2%)	52 (21·2%)	0·40
Regular alcohol consumption	396 (80·7%)	161 (65·7%)	< 0·0001
Atrial fibrillation	91 (18·5%)	57 (23·3%)	0·20
Prior history of stroke	52 (10·6%)	27 (11·0%)	1·00
Ischaemic heart disease	61 (12·4%)	19 (7·8%)	0·07
BMI (kg/m^2)	27·1±4·0	26·7±4·8	0·30
Systolic blood pressure (mmHg)	140 (129-151)	138 (128-150)	0·20
Diastolic blood pressure (mmHg)	80 (73-88)	78 (70-84)	0·0002
HbA_{1c} (%)	5·7 (5·4-6·2)	5·7 (5·4-6·1)	0·30
LDL cholesterol (mg/dL)	124 (102-150)	132 (104-156)	0·02
HDL cholesterol (mg/dL)	45 (38-52)	56 (46·4-64·5)	<0·0001
Triglycerides (mg/dL)	122 (91-177)	120 (91-151)	0·20
**Criteria for Metabolic syndrome*[2]**			
Abdominal obesity	225/457 (49·2%)	166/232 (71·6%)	<0·0001
Elevated triglycerides	168/463 (36·3%)	65/229 (28·4%)	0·05
Reduced HDL cholesterol	150/475 (31·6%)	81/235 (34·5%)	0·50
Elevated blood pressure	432/490 (88·2%)	221/245 (90·2%)	0·48
Prediabetes/Diabetes mellitus	263/462 (56·9%)	123/233 (52·8%)	0·34
Metabolic syndrome (≥3 of the above components present)	237 (48·3%)	128 (52·2%)	0·35
**Index stroke classification**			
Ischaemic stroke	475 (96·7%)	240 (98·0%)	0·48
TOAST classification of acute ischaemic stroke subtype			0·03
Large artery atherosclerosis	117 (23·8%)	49 (20·0%)	··
Cardioembolism	97 (19·8%)	67 (27·3%)	··
Small artery occlusion	55 (11·2%)	31 (12·7%)	··
Other determined aetiology	25 (5·1%)	4 (1·6%)	··
Undetermined aetiology	181 (36·9%)	89 (36·3%)	··
Haemorrhagic stroke	16 (3·3%)	5 (2·0%)	0·48
**Acute stroke treatment**			
Any reperfusion therapy (IVT and/or EVT)	139 (28·3%)	70 (28·6%)	1·00
**Neuroimaging parameters**			
Normalised brain volume (%)	67·8 (64·4-71·6)	67·6 (63·9-71·6)	0·74
Infarct volume (mm^3)	2352 (528-14960)	2168 (520-7256)	0·03
Normalised stroke lesion volume (%)	0·16 (0·03-0·97)	0·15 (0·03-0·54)	0·12
Small vessel disease score			0·17
0	180/444 (40·5%)	79/222 (35·6%)	··
1	126/444 (28·4%)	75/222 (33·8%)	··
2	85/444 (19·1%)	51/222 (23·0%)	··
3	42/444 (8·5%)	12/222 (5·4%)	··
4	11/444 (2·5%)	5/222 (2·2%)	··
Lacune count	0 (0-0)	0 (0-0)	0·01
≥3 lacunes	10/446 (2·2%)	2/225 (0·9%)	0·30

Normalised white matter hyperintensity volume (%)	0·20 (0·06-0·50)	0·24 (0·10-0·60)	0·01
Cerebral microbleed count	0 (0-0)	0 (0-0)	0·23
Perivascular space grade	1 (1-2)	1 (1-2)	0·50
Mean skeletonised mean diffusivity (z-score)	-0·22 (-0·81-0·48)	-0·05 (-0·63-0·77)	0·009
**Genetic risk factors**			
**APOE genotype**			0·36
0 ε4 allele	307/400 (76·7%)	156/194 (80·4%)	··
1 ε4 allele	88/400 (22·0%)	34/194 (17·5%)	··
2 ε4 alleles	5/400 (1·2%)	4/194 (2·1%)	··
**Pre-stroke clinical/cognitive function**			
mRS before stroke	0 (0-0)	0 (0-0)	0·71
IQCODE score	48 (48-49)	48 (48-49)	0·68

Data are n (%), median (IQR), mean (SD), or n/N (%). APOE=apolipoprotein E. BMI=body-mass index. EVT=endovascular thrombectomy. HbA_{1c}=glycated haemoglobin. HDL=high-density lipoprotein. IQCODE=Informant Questionnaire on Cognitive Decline in the Elderly. IVT=intravenous thrombolysis. LDL=low-density lipoprotein. MoCA=Montreal Cognitive Assessment. mRS=Modified Rankin Scale. NIHSS=National Institutes of Health Stroke Scale. TOAST=Trial of Org 10172 in Acute Stroke Treatment.

*[1] MoCA <26 or mini-mental state examination <27 when MoCA was not available (n=73).

*[2] Defined according to Alberti et al.[2]

Table S3: Loss to follow-up and death by study centre

	Loss to follow-up (N = 179)					
**Study centre**	**Initial N**	**Between BL and FU6**	**Between FU6 and FU12**	**Between FU12 and FU36**	**Between FU36 and FU60**	**Total lost**
DEDEMAS	136	4	7	3	10	24 (17·6%)
DEMDAS	600					
Munich-LMU	219	19	6	18	12	55 (25·1%)
Munich-TUM	69	11	3	3	2	19 (27·5%)
Berlin-1	33	0	2	0	1	3 (9·1%)
Berlin-2	38	5	0	2	0	7 (18·4%)
Bonn	105	10	8	5	6	29 (27·6%)
Göttingen	81	8	3	3	8	22 (27·2%)
Magdeburg	55	9	3	7	1	20 (36·4%)
**Total**	736	66	32	41	40	179 (24·3%)

Death (before LTFU, N = 63)

**Study centre**	**Initial N**	**Between BL and FU6**	**Between FU6 and FU12**	**Between FU12 and FU36**	**Between FU36 and FU60**	**Total died**
DEDEMAS	136	3	1	9	2	15 (11·0%)
DEMDAS	600					
Munich-LMU	219	9	2	7	10	28 (13·2%)
Munich-TUM	69	1	2	··	1	4 (5·8%)
Berlin-1	33	··	1	1	2	4 (12·1%)
Berlin-2	38	··	··	··	4	4 (10·5%)
Bonn	105	··	··	1	1	2 (1·9%)
Göttingen	81	1	··	2	··	3 (3·7%)
Magdeburg	55	1	··	1	1	3 (5·4%)
**Total**	736	15	6	21	21	63 (8·6%)

Breakdown of patients lost to follow-up (top) and who died (bottom) during the 5-year study period, by centre and follow-up period. Deaths do not include 5 deaths which occurred after the patients were lost to follow-up.

Table S4: Reasons for death and loss to follow-up during 5 years of follow-up

**Reason for drop-out**	**Cause/Reason**	**n (%)**
**Death (n = 68) ***	Brain haemorrhage	3 (4·4)
	Cardiac failure/arrest	4 (5·9)
	Consequences of SARS-CoV2 infection	1 (1·5)
	Infections	3 (4·4)
	Malignant neoplasms	13 (19·1)
	Myocardial infarction	4 (5·9)
	Pulmonary fibrosis	1 (1·5)
	Recurrent infarct	1 (1·5)
	Sepsis/multiorgan failure	10 (14·7)
	Stroke-related complications	2 (2·9)
	Unknown	26 (38·2)
**Loss to Follow-up (n = 179)**	Failed attempt to get in contact	21 (11·7)
	Moved/distance too long	8 (4·5)
	Other disease	5 (2·8)
	Other/Unknown	5 (2·8)
	Poor general condition	36 (20·1)
	Psychiatric disorder	2 (1·1)
	Revocation of consent	102 (57·0)

*Five deaths were recorded after the patients were lost to follow-up, three due to multiorgan failure, the other two due to an unknown cause

Table S5: Baseline characteristics of stroke survivors who dropped out of the study due to death or loss to follow-up and those who did not

	Followed up until the end of the study (n = 494)	Dropped out early (death or LTFU, n = 242)	P-value
**Sociodemographic variables**			
Age (years)	66·4±10·8	71·4±11·1	< 0·0001
Age ≥74 years	148 (30·0%)	113 (46·7%)	< 0·0001
Female sex	173 (35·0%)	72 (29·8%)	0·18
Education (years)	13 (12-17)	13 (11-15)	0·02
Education ≤12 years	187 (37·9%)	105 (43·4%)	0·17
**Clinical/cognitive acute phase deficits**			
Admission NIHSS score	3 (1-5)	3 (1-5)	0·57
Admission NIHSS ≥3	250 (50·6%)	137 (56·6%)	0·15
Barthel Index score	100 (85-100)	95 (72-100)	0·0003
Delirium rating scale score	0 (0-1)	0 (0-1)	0·31
Acute phase MoCA score	26 (23-28)	24 (22-26)	< 0·0001
Acute phase cognitive impairment*[1]	225 (46·3%)	157 (70·4%)	< 0·0001
**Cardiovascular risk factors**			
Hypertension	372 (75·3%)	199 (82·2%)	0·04
Diabetes mellitus	90 (18·2%)	60 (24·8%)	0·05
Dyslipidaemia	147 (29·8%)	82 (33·9%)	0·29
Current smoking	1110 (22·3%)	61 (25·2%)	0·43
Regular alcohol consumption	377 (76·3%)	180 (74·4%)	0·63
Atrial fibrillation	84 (17·0%)	64 (26·4%)	0·004
Prior history of stroke	45 (9·1%)	34 (14·0%)	0·06
Ischaemic heart disease	51 (10·3%)	29 (12·0%)	0·58
BMI (kg/m^2)	27·1±4·2	26·9±4·4	0·48
Systolic blood pressure (mmHg)	138 (128-150)	142 (130-152)	0·03
Diastolic blood pressure (mmHg)	80 (70-86)	80 (73-88)	0·30
HbA_{1c} (%)	5·7 (5·4-6·1)	5·8 (5·5-6·2)	0·04
LDL cholesterol (mg/dL)	124 (103-152)	129 (103-156)	0·47
HDL cholesterol (mg/dL)	49 (40-59)	45 (38-5)	0·01
Triglycerides (mg/dL)	122 (91-172)	118 (90-163)	0·52
**Criteria for Metabolic syndrome*[2]**			
Abdominal obesity	266/469 (56·7%)	125/220 (56·8%)	1·00
Elevated triglycerides	158/462 (34·2%)	75/230 (32·6%)	0·74
Reduced HDL cholesterol	144/476 (30·3%)	87/233 (37·4%)	0·07
Elevated blood pressure	434/493 (88·0%)	219/242 (90·5%)	0·38
Prediabetes/Diabetes mellitus	247/463 (53·3%)	139/232 (59·9%)	0·12
Metabolic syndrome (≥3 of the above components present)	233 (47·2%)	132 (54·5%)	0·07
**Index stroke classification**			
Ischaemic stroke	480 (97·2%)	235 (97·1%)	0·83
TOAST classification of acute ischaemic stroke subtype			0·40
Large artery atherosclerosis	113 (22·9%)	53 (21·9%)	··
Cardioembolism	98 (20·0%)	65 (26·9%)	··
Small artery occlusion	57 (11·5%)	29 (12·0%)	··
Other determined aetiology	20 (4·0%)	9 (3·7%)	··
Undetermined aetiology	191 (38·7%)	79 (32·6%)	··
Haemorrhagic stroke	14 (2·8%)	7 (2·9%)	0·83
**Acute stroke treatment**			
Any reperfusion therapy (IVT and/or EVT)	152 (30·8%)	57 (23·6%)	0.05
**Neuroimaging parameters**			
Normalised brain volume (%)	68·6 (65·1-72·3)	66·0 (62·5-69·9)	< 0·0001
Infarct volume (mm^3)	2484 (512-13122)	2088 (546-9830)	0·57
Normalised stroke lesion volume (%)	0·16 (0·03-0·81)	0·14 (0·04-0·62)	0·59
Small vessel disease score			0·002
0	194/456 (42·5%)	65/210 (31·0%)	··
1	141/456 (30·9%)	60/210 (28·6%)	··
2	84/456 (18·4%)	52/210 (24·8%)	··
3	30/456 (6·6%)	24/210 (11·4%)	··
4	7/456 (1·5%)	9/210 (4·3%)	··
Lacune count	0 (0-0)	0 (0-0)	0·003
≥3 lacunes	4/457 (0·9%)	8/214 (3·7%)	0·02
Normalised white matter hyperintensity volume (%)	0·20 (0·06-0·43)	0·33 (0·11-0·81)	< 0·0001
Cerebral microbleed count	0 (0-0)	0 (0-0)	0·47
Perivascular space grade	1 (1-2)	1 (1-2)	0·0002
Mean skeletonised mean diffusivity (z-score)	-0·26 (-0·88-0·43)	0·19 (-0·45-1·02)	< 0·0001
**Genetic risk factors**			
APOE genotype			0·06
0 ε4 allele	324/406 (79·8%)	139/188 (73·9%)	··
1 ε4 allele	74/406 (18·2%)	48/188 (25·5%)	··
2 ε4 alleles	8/406 (2·0%)	1/188 (0·53%)	··
**Pre-stroke clinical/cognitive function**			

mRS before stroke	0 (0-0)	0 (0-0)	0·12
IQCODE score	48 (48-49)	48 (48-50)	0·03

Data are n (%), median (IQR), mean (SD), or n/N (%). APOE=apolipoprotein E. BMI=body-mass index. EVT=endovascular thrombectomy. HbA_{1c}=glycated haemoglobin. HDL=high-density lipoprotein. IQCODE=Informant Questionnaire on Cognitive Decline in the Elderly. IVT=intravenous thrombolysis. LDL=low-density lipoprotein. MoCA=Montreal Cognitive Assessment. mRS=Modified Rankin Scale. NIHSS=National Institutes of Health Stroke Scale. TOAST=Trial of Org 10172 in Acute Stroke Treatment.

*[1] MoCA <26 or mini-mental state examination <27 when MoCA was not available (n=73).

*[2] Defined according to Alberti et al.[2]

Table S6: Baseline characteristics of stroke survivors with early- versus delayed-onset PSD

	Early-onset PSD (n = 21)	Delayed-onset PSD (n = 34)	P-value
**Sociodemographic variables**			
Age (years)	79·4 (8·6)	74·7 (9·4)	0·07
Age ≥74 years	17 (81·0%)	21 (61·8%)	0·20
Female sex	7 (33·3%)	12 (35·3%)	1·00
Education (years)	13 (11-14)	12 (11-13)	0·70
Education ≤12 years	10 (58·8%)	20 (47·6%)	0·60
**Clinical/cognitive acute phase deficits**			
Admission NIHSS score	4 (3-9)	3 (3-6)	0·14
Admission NIHSS ≥3	16 (76·2%)	26 (76·5%)	1·00
Barthel Index score	65 (55-75)	85 (60-95)	0·05
Delirium rating scale score	0 (0-1)	0 (0-4)	0·20
Acute phase MoCA score	19 (17-22)	23 (20-25)	0·04
Acute phase cognitive impairment*[1]	16/20 (90·0%)	26/29 (89·7%)	1·00
**Cardiovascular risk factors**			
Hypertension	18 (85·7%)	30 (88·2%)	0·10
Diabetes mellitus	9 (42·9%)	12 (35·5%)	0·80
Dyslipidaemia	8 (38·1%)	17 (50·0%)	0·60
Current smoking	0 (0·0%)	6 (17·6%)	0·10
Atrial fibrillation	12 (57·1%)	10 (29·4%)	0·08
Prior history of stroke	6 (28·6%)	5 (14·7%)	0·40
Ischaemic heart disease	5 (23·8%)	7 (20·6%)	1·00
BMI (kg/m^2)	26·3 (4·0)	26·4 (4·5)	0·90
Systolic blood pressure (mmHg)	149 (136-155)	144 (126-150)	0·10
Diastolic blood pressure (mmHg)	79 (75-85)	80 (72-85)	0·80
HbA_{1c} (%)	5·9 (5·6-6·8)	5·7 (5·6-6·8)	0·60
LDL cholesterol (mg/dL)	101 (86-129)	125 (92-166)	0·10
HDL cholesterol (mg/dL)	42 (36-54)	43 (36-58)	0·90
Triglycerides (mg/dL)	103 (74-148)	118 (95-219)	0·30
**Metabolic syndrome components*[2]**			
Abdominal obesity	7/16 (43·8%)	21/32 (65·6%)	0·30
Elevated triglycerides	5/20 (25·0%)	13/32 (40·6%)	0·40
Reduced HDL cholesterol	10/20 (50·0%)	17/32 (53·1%)	1·00
Elevated blood pressure	20/21 (95·2%)	30/34 (88·2%)	0·70
Prediabetes/Diabetes mellitus	15/20 (75·0%)	24/33 (72·7%)	1·00
Metabolic syndrome (≥3 of the above components present)	11 (52·4%)	25 (73·5%)	0·20
**Index stroke classification**			
Ischaemic stroke	20 (95·2%)	31 (91·2%)	1·00
TOAST classification of acute ischaemic stroke subtype			0·28
Large artery atherosclerosis	6 (28·6%)	6 (17·6%)	··
Cardioembolism	9 (42·9%)	11 (32·4%)	··
Small artery occlusion	0 (0·0%)	2 (5·9%)	··
Other determined aetiology	1 (4·8%)	0 (0·0%)	··
Undetermined aetiology	4 (19·0%)	12 (35·3%)	··
Haemorrhagic stroke	1 (4·8%)	3 (8·8%)	1·00
**Acute stroke treatment**			
**Any reperfusion therapy (IVT and/or EVT)**	5 (23·8%)	6 (17·6%)	0·83
**Neuroimaging parameters**			
Normalised brain volume (%)	62·4 (61·3-64·9)	64·3 (61·5-66·7)	0·30
Infarct volume (mm^3)	3752 (1092-29020)	1720 (460-7140)	0·20
Normalised stroke lesion volume (%)	0·28 (0·07-1·83)	0·13 (0·03-0·47)	0·10
Small vessel disease score			0·20
0	4/20 (20·0%)	4/31(12·9%)	··
1	5/20 (25·0%)	17/31(54·8%)	··
2	5/20 (25·0%)	6/31(19·4%)	··
3	3/20 (15·0%)	3/31(9·7%)	··
4	3/20 (15·0%)	1/3 (3·2%)	··
Lacune count	0 (0-0)	0 (0-1)	0·06
≥3 lacunes	2/21 (9·5%)	3/32 (9·4%)	1·00
Normalised white matter hyperintensity volume (%)	0·59 (0·24-1·53)	0·39 (0·23-1·25)	0·70
Cerebral microbleed count	0 (0-0)	0 (0-0)	0·50
Perivascular space grade	2 (1-3)	1 (1-2)	0·20
Mean skeletonised mean diffusivity (z-score)	1·03 (0·25-2·24)	0·46 (-0·18-1·79)	0·20
**Genetic risk factors**			
APOE genotype			0·60
0 ε4 allele	10/16 (62·5%)	21/27 (77·8%)	··
1 ε4 allele	5/16 (31·3%)	5/27 (18·5%)	··
2 ε4 alleles	1/16 (6·3%)	1/27 (3·7%)	··
**Pre-stroke clinical/cognitive function**			
mRS before stroke	0 (0-0)	0 (0-0)	0·80

IQCODE score	50 (48-52)	48 (48-51)	0·60

Data are n (%), median (IQR), mean (SD), or n/N (%). APOE=apolipoprotein E. BMI=body-mass index. HbA_{1c}=glycated haemoglobin. HDL=high-density lipoprotein. IQCODE=Informant Questionnaire on Cognitive Decline in the Elderly. LDL=low-density lipoprotein. MoCA=Montreal Cognitive Assessment. mRS=Modified Rankin Scale. NIHSS=National Institutes of Health Stroke Scale. TOAST=Trial of Org 10172 in Acute Stroke Treatment.

*[1] MoCA <26 or mini-mental state examination <27 when MoCA was not available (n=73).

*[2] Defined according to Alberti et al.[2]

Table S7: Risk factors for post-stroke dementia diagnosed before and after 6 months

	Early-onset dementia risk (3-6 months)			*Delayed-onset dementia risk (>6 months)*		
**Baseline risk factor**	**Cases/N**	**HR (95% CI)**	**P-value**	**Cases/N**	**HR (95% CI)**	**P-value**
Age (per year)	21/706	1·18 (1·11-1·26)	< 0·0001	34/617	1·10 (1·06- 1·15)	< 0·0001
Age ≥ 74	21/706	8·19 (2·75-24·42)	0·0002	34/617	3·63 (1·80-7·31)	0·0003
Female sex	21/706	0·49 (0·19-1·28)	0·15	34/617	0·46 (0·21-0·99)	0·05
Education (per year)	21/706	0·89 (0·78-1·02)	0·11	34/617	0·84 (0·75-0·95)	0·005
Education ≤ 12	21/706	1·40 (0·58-3·34)	0·45	34/617	2·27 (1·12-4·61)	0·02
**Clinical/cognitive acute phase deficits**						
Stroke severity (per point on admission NIHSS)	21/706	1·09 (1·01-1·18)	0·02	34/617	1·07 (1·00-1·14)	0·05
Admission NIHSS ≥3	21/706	2·70 (0·99-7·38)	0·05	34/617	2·65 (1·19-5·89)	0·02
Barthel Index (per point)	21/704	0·97 (0·96-0·99)	0·0002	34/615	0·98 (0·97-1·00)	0·01
Delirious symptoms (per point on DRS)	21/706	1·29 (1·13-1·46)	< 0·0001	34/617	1·00 (0·80-1·26)	0·96
Acute phase cognitive function (per point on MoCA)	15/625	0·79 (0·70-0·89)	< 0·0001	26/552	0·85 (0·78-0·94)	0·0009
Acute phase cognitive impairment*[1]	20/683	5·02 (1·15-21·92)	0·03	29/599	6·51 (1·95-21·75)	0·002
**Cardiovascular risk factors**						
Hypertension	21/706	0·85 (0·25-2·91)	0·79	34/617	1·20 (0·42-3·46)	0·73
Diabetes mellitus	21/706	2·44 (1·02-5·83)	0·04	34/617	2·18 (1·07-4·43)	0·03
Dyslipidaemia	21/706	0·97 (0·40-2·34)	0·95	34/617	1·64 (0·83-3·23)	0·15
Current smoking	21/706	0·00 (0·00-Inf)	0·99	34/617	1·52 (0·61-3·79)	0·37
Regular alcohol consumption	21/706	0·36 (0·15-0·86)	0·02	34/617	1·30 (0·53-3·18)	0·56
Atrial fibrillation	21/706	3·71 (1·56-8·83)	0·003	34/617	1·20 (0·57-2·53)	0·63
Prior history of stroke	21/706	2·86 (1·11-7·41)	0·03	34/617	1·54 (0·59-4·01)	0·38
Ischaemic heart disease	21/706	2·09 (0·76-5·74)	0·15	34/617	1·90 (0·82-4·38)	0·13
BMI (kg/m^2)	21/706	1·00 (0·89-1·13)	0.97	34/617	0·99 (0·90-1·09)	0·87
Systolic blood pressure (mmHg)	21/701	1·02 (0·99-1·04)	0·19	34/612	0·99 (0·97-1·01)	0·43
Diastolic blood pressure (mmHg)	21/701	1·00 (0·97-1·04)	0·76	34/612	1·00 (0·98-1·03)	0·74
HbA_{1c} (%)	20/658	1·06 (0·90-1·25)	0·47	32/573	1·06 (0·93-1·21)	0·37
LDL cholesterol (mg/dL)	20/684	0·99 (0·98-1·00)	0·19	33/598	1·00 (1·00-1·01)	0·27
HDL cholesterol (mg/dL)	20/679	0·98 (0·94-1·01)	0·18	32/593	0·98 (0·95-1·01)	0·14
Triglycerides (mg/dL)	20/663	1·00 (0·99-1·01)	0·92	32/578	1·00 (1·00-1·01)	0·01
**Metabolic syndrome components*[2]**						
Abdominal obesity	16/666	0·57 (0·21-1·55)	0·27	32/587	1·33 (0·62-2·84)	0·46
Elevated triglycerides	20/663	0·92 (0·33-2·54)	0·88	32/578	2·11 (1·02-4·35)	0·04
Reduced HDL cholesterol	20/679	2·30 (0·94-5·59)	0·07	32/593	2·82 (1·38-5·76)	0·004
Elevated blood pressure	21/705	1·02 (0·13-7·65)	0·99	34/616	0·32 (0·11-0·95)	0·04
Prediabetes/Diabetes mellitus	20/666	2·05 (0·74-5·56)	0·16	33/580	2·17 (0·98-4·80)	0·06
Metabolic syndrome (≥3 of the above components present)	21/706	1·04 (0·44-2·46)	0·93	34/617	3·46 (1·52-7·85)	0·003
Per count of components increase	21/706	1·08 (0·75-1·56)	0·67	34/617	1·46 (1·10-1·94)	0·009
**Index stroke classification**						
Haemorrhagic stroke	21/706	1·46 (0·19-10·94)	0·71	34/617	3·72 (1·12-12·41)	0·03
**Acute stroke treatment**						

Any reperfusion therapy (IVT and/or EVT)	21/706	0·47 (0·16-1·37)	0·17	34/617	0·28 (0·11-0·74	0·01
**Neuroimaging parameters**						
Normalised brain volume (per SD)	19/634	0·52 (0·29-0·92)	0·02	31/559	0·66 (0·42-1·04)	0·07
Normalised infarct volume (per SD)	19/634	1·38 (1·04-1·84)	0·03	31/559	1·00 (0·66-1·51)	0·98
Total SVD score (per SD)	20/643	1·50 (0·96-2·33)	0·07	31/567	1·09 (0·74-1·61)	0·65
Lacune count (per SD)	21/647	1·32 (1·13-1·55)	0·0005	32/570	1·49 (1·19-1·85)	0·0004
Presence of ≥3 lacunes	21/647	6·93 (1·60-30·07)	0·01	32/570	17·43 (5·08-59·76)	< 0·0001
Normalised WMH volume (per SD)	19/633	1·31 (0·97-1·76)	0·08	29/558	1·51 (1·19-1·92)	0·0008
Cerebral microbleed count (per SD)	20/642	1·10 (0·83-1·48)	0·50	31/566	1·21 (1·00-1·46)	0·06
Perivascular space grade (per SD)	21/646	1·41 (0·96-2·05)	0·08	32/569	1·12 (0·81-1·57)	0·49
Mean skeletonised mean diffusivity (per SD)	17/606	1·89 (1·21-2·93)	0·005	28/536	1·97 (1·33-2·94)	0·0008
**Genetic risk factors**						
APOE genotype						
0 ε4 alleles	ref	ref	··	ref	ref	··
1 ε4 allele	16/576	1·79 (0·61-5·30)	0·29	27/508	0·79 (0·29-2·15)	0·64
2 ε4 alleles	16/576	8·94 (1·13-70·73)	0·04	27/508	3·37 (0·45-25·54)	0·24
**Pre-stroke clinical/cognitive function**						
mRS before stroke	21/706	1·04 (0·61-1·77)	0·89	34/617	1·15 (0·74-1·79)	0·52
IQCODE score	19/655	1·04 (0·91-1·18)	0·56	30/575	1·10 (0·98-1·24)	0·10
**Recurrent events**						
Stroke recurrence	21/757	0·62 (0·08-4·67)	0·68	34/650	3·94 (1·76-8·82)	0·0009

Cox proportional hazards regression models for the association between risk factors and early-onset (left) and delayed-onset PSD (right). Recurrent stroke was included as a time-dependent variable as described in the **Supplementary Methods**.

APOE=apolipoprotein E. BMI=body-mass index. DRS=Delirious rating scale. EVT=Endovascular thrombectomy. HbA_{1c}=glycated haemoglobin. HDL=high-density lipoprotein. IQCODE=Informant Questionnaire on Cognitive Decline in the Elderly. IVT=Intravenous thrombolysis. LDL=low-density lipoprotein. MoCA=Montreal Cognitive Assessment. mRS=Modified Rankin Scale. NIHSS=National Institutes of Health Stroke Scale. WMH=white matter hyperintensity.

*[1] MoCA <26 or mini-mental state examination <27 when MoCA was not available (n=73).

*[2] Defined according to Alberti et al.[2]

Table S8: Subgroup analysis stratifying the main analyses by sex

	Male			*Female*		
**Risk Factor**	**Cases/N**	**HR (95% CI)**	**P-Value**	**Cases/N**	**HR (95% CI)**	**P-Value**
**Sociodemographic factors**						
Age (per year)	36/491	1·15 (1·09-1·20)	<0·0001	19/245	1·11 (1·04-1·18)	0·001
Age ≥74	36/491	6·38 (3·08-13·24)	<0·0001	19/245	2·95 (1·10-7·94)	0·03
Education (per year)	36/491	0·82 (0·73-0·93)	0·002	19/245	0·92 (0·79-1·07)	0·28
Education ≤12	36/491	2·87 (1·46-5·62)	0·002	19/245	0·92 (0·36-2·34)	0·85
**Clinical/cognitive acute phase deficits**						
Stroke severity (per point on admission NHSS)	36/491	1·10 (1·02-1·17)	0·009	19/245	1·06 (0·99-1·14)	0·10
Admission NIHSS ≥3	36/491	3·08 (1·42-6·70)	0·004	19/245	1·40 (0·52-3·77)	0·51
Barthel Index (per point)	36/488	0·97 (0·96-0·99)	<0·0001	19/244	0·99 (0·97-1·01)	0·36
Delirious symptoms (per point on DRS)	36/491	1·11 (0·96-1·28)	0·15	19/245	1·39 (1·12-1·74)	0·003
Acute phase cognitive function (per point on MoCA)	26/436	0·85 (0·78-0·93)	0·0005	15/211	0·78 (0·69-0·89)	0·0003
Acute phase cognitive impairment*[1]	32/476	7·61 (1·78-32·52)	0·006	17/233	5·06 (1·41-18·14)	0·01
**Vascular risk factors**						
Hypertension	36/491	0·92 (0·38-2·22)	0·84	19/245	2·02 (0·26-15·99)	0·51
Diabetes mellitus	36/491	3·56 (1·83-6·94)	0·0002	19/245	0·72 (0·21-2·53)	0·61
Dyslipidaemia	36/491	1·88 (0·98-3·63)	0·06	19/245	0·78 (0·30-2·02)	0·61
Current smoking	36/491	0·53 (0·16-1·76)	0·30	19/245	1·85 (0·50-6·88)	0·36
Regular alcohol consumption	36/491	0·97 (0·40-2·36)	0·95	19/245	0·48 (0·19-1·19)	0·11
Atrial fibrillation	36/491	1·45 (0·72-2·91)	0·29	19/245	3·19 (1·25-8·15)	0·02
Prior stroke	36/491	2·21 (0·96-5·12)	0·06	19/245	2·03 (0·66-6·26)	0·22
Ischaemic heart disease	36/491	2·80 (1·37-5·75)	0·005	19/245	0·59 (0·08-4·46)	0·61
BMI (kg/m^2)	36/490	1·07 (0·97-1·17)	0·19	19/245	0·89 (0·78-1·03)	0·12
SBP (mmHg)	36/488	1·01 (0·99-1·03)	0·32	19/243	0·99 (0·96-1·01)	0·36
DBP (mmHg)	36/488	1·00 (0·97-1·03)	0·90	19/243	1·02 (0·98-1·05)	0·38
HbA1c (%)	35/456	1·07 (0·97-1·18)	0·16	17/230	0·85 (0·42-1·70)	0·64
LDL-C (mg/dL)	35/476	1·00 (0·99-1·01)	0·62	18/238	1·01 (0·99-1·02)	0·33
HDL-C (mg/dL)	35/474	0·96 (0·93-0·99)	0·01	17/235	0·99 (0·97-1·02)	0·72
Triglycerides (mg/dL)	35/463	1·00 (1·00-1·01)	0·17	17/229	1·01 (1·00-1·01)	0·02
**Metabolic syndrome components*[2]**						
Abdominal obesity	31/457	1·18 (0·58-2·41)	0·65	17/232	0·71 (0·25-2·07)	0·53
Elevated triglycerides	35/463	1·48 (0·74-2·96)	0·26	17/229	2·03 (0·68-6·07)	0·21
Reduced HDL-C	35/474	2·75 (1·39-5·45)	0·004	17/235	2·53 (0·92-6·95)	0·07
Elevated blood pressure	36/490	0·22 (0·08-0·61)	0·004	19/245	27302244·33 (0·00-Inf)	1·00
Prediabetes or diabetes mellitus	36/462	2·42 (1·10-5·33)	0·03	17/233	1·82 (0·59-5·63)	0·30
Metabolic syndrome (≥3 of the above components present)	36/491	2·09 (1·05-4·18)	0·04	19/245	2·22 (0·75-6·58)	0·15
Per count of components increase	36/491	1·36 (1·04-1·78)	0·02	19/245	1·23 (0·80-1·89)	0·34
**Index stroke classification**						
Haemorrhagic stroke	36/491	2·98 (1·04-8·52)	0·04	19/245	0·00 (0·00-Inf)	1·00
**Acute stroke treatment**						
Any reperfusion therapy (IVT and/or EVT)	36/491	0·24 (0·08-0·69)	0·008	19/245	0.36 (0.09-1.39)	0.14

**Neuroimaging parameters**						
Normalised brain volume (per SD)	33/438	0·59 (0·37-0·94)	0·03	17/219	0·68 (0·35-1·34)	0·26
Normalised infarct volume (per SD)	33/439	1·13 (0·85-1·50)	0·40	17/219	1·85 (1·00-3·41)	0·05
Total SVD score (per SD)	34/444	1·36 (0·96-1·91)	0·08	17/222	1·08 (0·59-1·96)	0·81
Lacune count (per SD)	35/446	1·38 (1·21-1·58)	<0·0001	18/225	1·30 (0·84-2·01)	0·24
Presence of ≥3 lacunes	35/446	10·30 (3·52-30·10)	<0·0001	18/225	19·76 (2·32-168·44)	0·006
Normalised WMH volume (per SD)	32/438	1·57 (1·28-1·92)	<0·0001	16/219	1·10 (0·72-1·66)	0·66
CMB count (per SD)	34/444	1·26 (1·01-1·57)	0·04	17/222	1·14 (0·89-1·46)	0·31
PVS grade (per SD)	35/446	1·39 (1·01-1·92)	0·04	18/224	1·03 (0·65-1·61)	0·91
Mean skeletonised mean diffusivity (per SD)	29/414	1·85 (1·28-2·67)	0·001	16/214	2·29 (1·27-4·14)	0·006
**APOE genotype**						
1 ε4 allele	30/400	1·37 (0·57-3·28)	0·48	13/194	0·87 (0·22-3·54)	0·85
2 ε4 alleles	30/400	2·74 (0·36-21·04)	0·33	13/194	12·48 (1·27-122·94)	0·03
**Pre-stroke clinical/cognitive function**						
mRS before stroke	36/491	1·15 (0·74-1·79)	0·54	19/245	1·10 (0·61-2·00)	0·74
IQCODE score	35/452	0·96 (0·90-1·04)	0·32	14/224	1·34 (1·18-1·52)	<0·0001
**Recurrent events**						
Stroke recurrence	36/527	2·25 (0·98-5·17)	0·06	19/260	3·22 (0·69-15·12)	0·14

Cox proportional hazards regression models for the association between risk factors and PSD in males (left) and females (right), adjusted for age, education, and admission NIHSS. Recurrent stroke was included as a time-dependent variable as described in the **Supplementary Methods**.

APOE=apolipoprotein E. BMI=body-mass index. CMB = cerebral microbleed. DBP = diastolic blood pressure. EVT=Endovascular thrombectomy. HbA_{1c}=glycated haemoglobin. HDL-C=high-density lipoprotein cholesterol. IQCODE=Informant Questionnaire on Cognitive Decline in the Elderly. IVT=Intravenous thrombolysis. LDL-C=low-density lipoprotein cholesterol. MoCA=Montreal Cognitive Assessment. mRS=Modified Rankin Scale. NIHSS=National Institutes of Health Stroke Scale. PVS = perivascular space. SBP = systolic blood pressure. SD = standard deviation. SVD = small vessel disease. WMH = white matter hyperintensity.

*[1] MoCA <26 or mini-mental state examination <27 when MoCA was not available (n=73).

*[2] Defined according to Alberti et al.[2]

Table S9: Stroke recurrence during 5 years of follow-up

	One recurrent stroke (n=51)	Two recurrent strokes (n=5)
**Developed dementia during follow-up**		
No	27	5
Before recurrence	4	··
After recurrence	9	··
LTFU without dementia diagnosis	11	··
**Type of first recurrent stroke**		
Ischaemic	30*	4
Haemorrhagic	4	··
Unknown	17	1
**Type of second recurrent stroke**		
Ischaemic	··	4
Haemorrhagic	··	··
Unknown	··	1

Number of patients who experienced one or two recurrent strokes by dementia status and stroke subtype.
*one with haemorrhagic transformation

Table S10: Sensitivity analysis additionally adjusting the main analyses for acute stroke treatment

Baseline risk factor	Cases/N	HR (95% CI)	P-value
**Sociodemographic variables**			
Age (per year)	55/706	1·13 (1·09-1·17)	<0·0001
Age ≥ 74	55/706	4·33 (2·41-7·79)	<0·0001
Female sex	55/706	0·40 (0·21-0·77)	0·006
Education (per year)	55/706	0·86 (0·78-0·94)	0·001
Education ≤ 12	55/706	1·85 (1·06-3·23)	0·03
**Clinical/cognitive acute phase deficits**			
Stroke severity (per point on admission NIHSS)	55/706	1·12 (1·06-1·18)	<0·0001
Admission NIHSS ≥3	55/706	3·44 (1·80-6·57)	0·0002
Barthel Index (per point)	55/704	0·98 (0·97-0·99)	<0·0001
Delirious symptoms (per point on DRS)	55/706	1·15 (1·03-1·29)	0·01
Acute phase cognitive function (per point on MoCA)	41/625	0·84 (0·78-0·90)	<0·0001
Acute phase cognitive impairment*[1]	49/683	16·36 (4·36-61·39)	<0·0001
**Vascular risk factors**			
Hypertension	55/706	1·02 (0·45-2·28)	0·97
Diabetes mellitus	55/706	2·33 (1·34-4·05)	0·003
Dyslipidaemia	55/706	1·31 (0·76-2·25)	0·32
Current smoking	55/706	0·79 (0·33-1·89)	0·59
Regular alcohol consumption	55/706	0·83 (0·45-1·52)	0·54
Atrial fibrillation	55/706	2·07 (1·19-3·59)	0·01
Prior stroke	55/706	1·82 (0·91-3·63)	0·09
Ischaemic heart disease	55/706	1·86 (0·96-3·57)	0·06
BMI (kg/m^2)	55/706	1·01 (0·94-1·09)	0·79
SBP (mmHg)	55/701	1·00 (0·98-1·02)	0·97
DBP (mmHg)	55/701	1·00 (0·98-1·03)	0·80
HbA1c (%)	52/658	1·05 (0·94-1·16)	0·39
LDL-C (mg/dL)	53/684	1·00 (0·99-1·01)	0·97
HDL-C (mg/dL)	52/679	0·98 (0·96-1·00)	0·06
Triglycerides (mg/dL)	52/662	1·00 (1·00-1·01)	0·07
**Metabolic syndrome components*[2]**			
Abdominal obesity	48/666	1·17 (0·63-2·16)	0·62
Elevated triglycerides	52/662	1·49 (0·83-2·68)	0·18
Reduced HDL-C	52/679	2·68 (1·51-4·75)	0·0007
Elevated blood pressure	55/705	0·37 (0·14-0·98)	0·05
Prediabetes or diabetes	53/666	2·32 (1·23-4·38)	0·009
Metabolic syndrome (≥3 of the above components present)	55/706	2·18 (1·22-3·90)	0·008
Per count of components increase	55/706	1·33 (1·06-1·66)	0·01
**Index stroke classification**			
Haemorrhagic stroke	55/706	2·02 (0·71-5·77)	0·19
**Neuroimaging parameters**			
Normalised brain volume (per SD)	50/634	0·61 (0·42-0·90)	0·01
Normalised infarct volume (per SD)	50/634	1·19 (0·93-1·52)	0·17
Total SVD score (per SD)	51/642	1·17 (0·87-1·57)	0·31
Lacune count (per SD)	53/647	1·38 (1·21-1·57)	<0·0001
Presence of ≥3 lacunes	53/647	10·38 (3·94-27·33)	<0·0001
Normalised WMH volume (per SD)	48/633	1·37 (1·14-1·66)	0·001
CMB count (per SD)	51/642	1·15 (0·97-1·36)	0·10
PVS grade (per SD)	53/646	1·19 (0·92-1·54)	0·19
Mean skeletonised mean diffusivity (per SD)	45/606	1·88 (1·39-2·54)	<0·0001
**APOE genotype**			
1 ε4 allele	43/575	1·00 (0·47-2·13)	1·00
2 ε4 alleles	43/575	4·05 (0·93-17·57)	0·06
**Pre-stroke clinical/cognitive function**			
mRS before stroke	55/706	1·09 (0·77-1·55)	0·62
IQCODE score	49/655	1·07 (0·98-1·18)	0·13
**Recurrent events**			

Stroke recurrence	55/757	2·86 (1·37-5·93)	0·005

Cox proportional hazards regression models for the association between risk factors and post-stroke dementia, adjusted for age, sex, education, admission NIHSS, and acute stroke treatment (IVT and/or EVT). Recurrent stroke was included as a time-dependent covariable as described in the **Supplementary Methods**. APOE=apolipoprotein E. BMI=body-mass index. CMB = cerebral microbleed. DBP = diastolic blood pressure. EVT=Endovascular thrombectomy. HbA_{1c}=glycated haemoglobin. HDL-C=high-density lipoprotein cholesterol. IQCODE=Informant Questionnaire on Cognitive Decline in the Elderly. IVT=Intravenous thrombolysis. LDL-C=low-density lipoprotein cholesterol. MoCA=Montreal Cognitive Assessment. mRS=Modified Rankin Scale. NIHSS=National Institutes of Health Stroke Scale. PVS = perivascular space. SBP = systolic blood pressure. SD = standard deviation. SVD = small vessel disease. WMH = white matter hyperintensity.

*[1] MoCA <26 or mini-mental state examination <27 when MoCA was not available (n=73).

*[2] Defined according to Alberti et al.[2]

Table S11: Sensitivity analysis additionally adjusting the analysis split by post-stroke time period for age, sex, education, NIHSS, and acute stroke treatment

	Early-onset dementia risk (3-6 months)			*Delayed-onset dementia risk (>6 months)*		
**Baseline risk factor**	**Cases/N**	**HR (95% CI)**	**P-value**	**Cases/N**	**HR (95% CI)**	**P-value**
Age (per year)	21/706	1·18 (1·11-1·26)	< 0·0001	34/617	1·10 (1·05- 1·15)	< 0·0001
Age ≥ 74	21/706	7·46 (2·50-22·29)	0·0003	34/617	3·31 (1·63-6·70)	0·0009
Female sex	21/706	0·44 (0·17-1·14)	0·09	34/617	0·38 (0·17-0·85)	0·02
Education (per year)	21/706	0·89 (0·77-1·02)	0·09	34/617	0·84 (0·74-0·94)	0·004
Education ≤ 12	21/706	1·38 (0·58-3·29)	0·47	34/617	2·23 (1·10-4·52)	0·03
**Clinical/cognitive acute phase deficits**						
Stroke severity (per point on admission NIHSS)	21/706	1·14 (1·05-1·23)	0·0009	34/617	1·11 (1·04-1·19)	0·002
Admission NIHSS ≥3	21/706	3·40 (1·23-9·39)	0·02	34/617	3·47 (1·53-7·84)	0·003
Barthel Index (per point)	21/704	0·97 (0·96-0·99)	0·0001	34/615	0·98 (0·97-1·00)	0·01
Delirious symptoms (per point on DRS)	21/706	1·30 (1·14-1·48)	< 0·0001	34/617	0·98 (0·77-1·24)	0·85
Acute phase cognitive function (per point on MoCA)	15/625	0·80 (0·70-0·92)	0·001	26/552	0·86 (0·79-0·94)	0·001
Acute phase cognitive impairment*[1]	20/683	23·10 (3·83-139·19)	0·0006	29/599	8·14 (0·84-79·03)	0·07
**Cardiovascular risk factors**						
Hypertension	21/706	0·68 (0·19-2·41)	0·55	34/617	1·25 (0·43-3·60)	0·68
Diabetes mellitus	21/706	2·36 (0·97-5·71)	0·06	34/617	2·16 (1·06-4·41)	0·03
Dyslipidaemia	21/706	0·89 (0·37-2·18)	0·80	34/617	1·70 (0·86-3·39)	0·13
Current smoking	21/706	0·00 (0·00-Inf)	1·00	34/617	1·29 (0·51-3·26)	0·60
Regular alcohol consumption	21/706	0·37 (0·15-0·93)	0·03	34/617	1·58 (0·63-3·93)	0·33
Atrial fibrillation	21/706	3·73 (1·56-8·92)	0·003	34/617	1·40 (0·66-2·99)	0·38
Prior history of stroke	21/706	2·61 (0·98-6·94)	0·05	34/617	1·27 (0·47-3·43)	0·64
Ischaemic heart disease	21/706	1·87 (0·67-5·22)	0·23	34/617	1·77 (0·75-4·18)	0·19
BMI (kg/m^2)	21/706	1·03 (0·91-1·16)	0.62	34/617	0·99 (0·90-1·09)	0·83
Systolic blood pressure (mmHg)	21/701	1·02 (0·99-1·04)	0·22	34/612	0·99 (0·97-1·01)	0·37
Diastolic blood pressure (mmHg)	21/701	1·01 (0·97-1·04)	0·60	34/612	1·00 (0·97-1·03)	0·94
HbA_{1c} (%)	20/658	1·08 (0·90-1·28)	0·41	32/573	1·03 (0·90-1·17)	0·69
LDL cholesterol (mg/dL)	20/684	0·99 (0·98-1·00)	0·27	33/598	1·00 (0·99-1·01)	0·37
HDL cholesterol (mg/dL)	20/679	0·98 (0·94-1·01)	0·21	32/593	0·98 (0·95-1·01)	0·22
Triglycerides (mg/dL)	20/663	1·00 (0·99-1·01)	0·90	32/578	1·00 (1·00-1·01)	0·05
**Metabolic syndrome components*[2]**						
Abdominal obesity	16/666	0·45 (0·15-1·34)	0·15	32/587	1·76 (0·81-3·81)	0·15
Elevated triglycerides	20/663	0·91 (0·33-2·51)	0·85	32/578	1·93 (0·92-4·03)	0·08
Reduced HDL cholesterol	20/679	2·33 (0·91-5·98)	0·08	32/593	2·81 (1·36-5·80)	0·005
Elevated blood pressure	21/705	0·78 (0·10-6·04)	0·81	34/616	0·28 (0·09-0·86)	0·03
Prediabetes/Diabetes mellitus	20/666	2·36 (0·84-6·65)	0·10	33/580	2·19 (0·98-4·88)	0·05
Metabolic syndrome (≥3 of the above components present)	21/706	1·05 (0·44-2·54)	0·90	34/617	3·49 (1·54-7·92)	0·003
Per count of components increase	21/706	1·09 (0·74-1·59)	0·66	34/617	1·46 (1·10-1·94)	0·009
**Index stroke classification**						
Haemorrhagic stroke	21/706	1·16 (0·15-9·14)	0·89	34/617	2·82 (0·83-9·56)	0·09
**Neuroimaging parameters**						

Normalised brain volume (per SD)	19/634	0·67 (0·36-1·27)	0·22	31/559	0·59 (0·37-0·94)	0·03
Normalised infarct volume (per SD)	19/634	1·43 (1·03-1·97)	0·03	31/559	0·97 (0·64-1·47)	0·89
Total SVD score (per SD)	20/643	1·34 (0·83-2·17)	0·23	31/567	1·06 (0·71-1·56)	0·79
Lacune count (per SD)	21/647	1·37 (1·15-1·63)	0·0005	32/570	1·48 (1·17-1·85)	0·0008
Presence of ≥3 lacunes	21/647	7·27 (1·61-32·70)	0·01	32/570	15·20 (4·16-55·23)	< 0·0001
Normalised WMH volume (per SD)	19/633	1·24 (0·89-1·73)	0·21	29/558	1·48 (1·16-1·87)	0·001
Cerebral microbleed count (per SD)	20/642	1·07 (0·78-1·46)	0·67	31/566	1·17 (0·97-1·42)	0·10
Perivascular space grade (per SD)	21/646	1·30 (0·87-1·95)	0·20	32/569	1·12 (0·79-1·58)	0·52
Mean skeletonised mean diffusivity (per SD)	17/606	1·62 (0·97-2·70)	0·06	28/536	2·01 (1·37-2·96)	0·0004
**Genetic risk factors**						
APOE genotype						
0 ε4 alleles	ref	ref	··	ref	ref	··
1 ε4 allele	16/576	1·61 (0·54-4·82)	0·39	27/508	0·71 (0·25-1·98)	0·51
2 ε4 alleles	16/576	7·47 (0·94-59·61)	0·06	27/508	2·75 (0·36-21·07)	0·33
**Pre-stroke clinical/cognitive function**						
mRS before stroke	21/706	0·92 (0·53-1·59)	0·77	34/617	1·19 (0·76-1·88)	0·44
IQCODE score	19/655	1·05 (0·94-1·16)	0·36	30/575	1·11 (0·99-1·25)	0·08
**Recurrent events**						
Stroke recurrence	21/757	0·69 (0·09-5·17)	0·72	34/650	4·70 (2·08-10·62)	0·0002

Cox proportional hazards regression models for the association between risk factors and early-onset (left) and delayed-onset PSD (right), adjusting for age, sex, education, admission NIHSS, and acute stroke treatment (IVT and/or EVT). Recurrent stroke was included as a time-dependent variable as described in the **Supplementary Methods**. APOE=apolipoprotein E. BMI=body-mass index. CMB = cerebral microbleed. DBP = diastolic blood pressure. EVT=endovascular thrombectomy. HbA_{1c}=glycated haemoglobin. HDL-C=high-density lipoprotein cholesterol. IQCODE=Informant Questionnaire on Cognitive Decline in the Elderly. IVT=intravenous thrombolysis. LDL-C=low-density lipoprotein cholesterol. MoCA=Montreal Cognitive Assessment. mRS=Modified Rankin Scale. NIHSS=National Institutes of Health Stroke Scale. PVS = perivascular space. SBP = systolic blood pressure. SD = standard deviation. SVD = small vessel disease. WMH = white matter hyperintensity.

*[1] MoCA <26 or mini-mental state examination <27 when MoCA was not available (n=73).

*[2] Defined according to Alberti et al.[2]

Table S12: Sensitivity analysis additionally adjusting the main analyses for stroke recurrence

Baseline risk factor	Cases/N	HR (95% CI)	P-value
**Sociodemographic factors**			
Age (per year)	55/757	1·13 (1·09-1·18)	<0·0001
Age ≥74	55/757	4·70 (2·64-8·38)	<0·0001
Female sex	55/757	0·52 (0·28-0·98)	0·04
Education (per year)	55/757	0·86 (0·79-0·95)	0·002
Education ≤12	55/757	1·91 (1·09-3·36)	0·02
**Clinical acute phase deficits**			
Stroke severity (per point on **admission** NHSS)	55/757	1·08 (1·02-1·13)	0·004
Admission NIHSS ≥3	55/757	2·49 (1·33-4·67)	0·01
Barthel Index (per point)	55/755	0·98 (0·97-0·99)	<0·0001
Delirious symptoms (per point on DRS)	55/757	1·18 (1·05-1·32)	0·004
Acute phase cognitive function (per point on MoCA)	41/669	0·84 (0·78-0·90)	<0·0001
Acute phase cognitive impairment*[1]	49/733	6·38 (2·50-16·30)	0·0001
**Cardiovascular risk factors**			
Hypertension	55/757	0·95 (0·42-2·15)	0·91
Diabetes mellitus	55/757	2·06 (1·18-3·61)	0·01
Dyslipidaemia	55/757	1·23 (0·72-2·12)	0·45
Current smoking	55/757	1·01 (0·41-2·46)	0·98
Regular alcohol consumption	55/757	0·81 (0·44-1·50)	0·51
Atrial fibrillation	55/757	2·10 (1·21-3·65)	0·008
Prior stroke	55/757	1·85 (0·94-3·66)	0·07
Ischaemic heart disease	55/757	1·63 (0·84-3·18)	0·15
BMI (kg/m^2)	55/757	1·00 (0·92-1·08)	0·92
SBP (mmHg)	55/752	1·00 (0·99-1·02)	0·80
DBP (mmHg)	55/752	1·00 (0·98-1·03)	0·67
HbA_{1c} (%)	52/703	1·06 (0·96-1·18)	0·24
LDL-C (mg/dL)	53/731	1·00 (0·99-1·01)	0·66
HDL-C (mg/dL)	52/726	0·98 (0·96-1·00)	0·08
Triglycerides (mg/dL)	52/709	1·00 (1·00-1·01)	0·03
**Metabolic syndrome components*[2]**			
Abdominal obesity	48/716	0·95 (0·51-1·74)	0·86
Elevated triglycerides	52/709	1·50 (0·83-2·68)	0·18
Reduced HDL-C	52/726	2·56 (1·45-4·53)	0·001
Elevated blood pressure	55/756	0·43 (0·17-1·12)	0·08
Prediabetes or diabetes mellitus	53/712	2·14 (1·14-4·01)	0·02
Metabolic syndrome (≥3 of the above components present)	55/757	1·96 (1·09-3·50)	0·02
Per count of components increase	55/757	1·28 (1·02-1·60)	0·03
**Index stroke classification**			
Haemorrhagic stroke	55/757	3·04 (1·08-8·57)	0·03
**Acute stroke treatment**			
Any reperfusion therapy (IVT and/or EVT)	55/757	0·31 (0·14-0·66)	0·003
**Neuroimaging parameters**			
Normalised brain volume (per SD)	50/677	0·62 (0·43-0·91)	0·01
Normalised infarct volume (per SD)	50/677	1·21 (0·95-1·55)	0·13
Total SVD score (per SD)	51/686	1·23 (0·95-1·70)	0·10
Lacune count (per SD)	53/691	1·34 (1·17-1·53)	<0·0001
Presence of ≥3 lacunes	53/691	7·61 (2·71-21·33)	0·0001
Normalised WMH volume (per SD)	48/675	1·41 (1·16-1·71)	0·0006
CMB count (per SD)	51/686	1·17 (1·00-1·38)	0·05
PVS grade (per SD)	53/690	1·28 (0·99-1·66)	0·06
Mean skeletonised mean diffusivity (per SD)	45/647	1·91 (1·42-2·59)	<0·0001
**APOE genotype**			
1 ε4 allele	43/621	1·15 (0·55-2·42)	0·71
2 ε4 alleles	43/621	5·06 (1·19-21·50)	0·03
**Pre-stroke clinical/cognitive function**			
mRS before stroke	55/757	1·14 (0·81-1·62)	0·45
IQCODE score	49/704	1·08 (0·98-1·18)	0·13

Cox proportional hazards regression models for the association between risk factors and post-stroke dementia, adjusted for age, sex, education, admission NIHSS, and stroke recurrence. Recurrent stroke was included as a time-dependent covariable as described in the **Supplementary Methods**. APOE=apolipoprotein E. BMI=body-mass index. CMB = cerebral microbleed. DBP = diastolic blood pressure. EVT=Endovascular thrombectomy. HbA_{1c}=glycated haemoglobin. HDL-C=high-density lipoprotein cholesterol. IQCODE=Informant Questionnaire on Cognitive Decline in the Elderly. IVT=Intravenous thrombolysis. LDL-C=low-density lipoprotein cholesterol. MoCA=Montreal Cognitive Assessment. mRS=Modified Rankin Scale. NIHSS=National Institutes of Health Stroke Scale. PVS = perivascular space. SBP = systolic blood pressure. SD = standard deviation. SVD = small vessel disease. WMH = white matter hyperintensity.
*[1] MoCA <26 or mini-mental state examination <27 when MoCA was not available (n=73).
*[2] Defined according to Alberti et al.[2]

Table S13: Sensitivity analysis adjusting the analysis split by post-stroke time period for age, sex, education, NIHSS, and stroke recurrence

	Dementia risk 3-6 months			*Dementia risk >6 months*		
**Baseline risk factor**	**Cases/N**	**HR (95% CI)**	**P-value**	**Cases/N**	**HR (95% CI)**	**P-value**
Age (per year)	21/757	1·19 (1·12-1·27)	<0·0001	34/650	1·10 (1·05-1·15)	<0·0001
Age ≥74	21/757	8·13 (2·73-24·20)	0·0002	34/650	3·59 (1·78-7·22)	0·0003
Female sex	21/757	0·56 (0·22-1·45)	0·23	34/650	0·50 (0·23-1·08)	0·08
Education (per year)	21/757	0·90 (0·79-1·02)	0·11	34/650	0·84 (0·75-0·95)	0·004
Education ≤12	21/757	1·42 (0·59-3·41)	0·43	34/650	2·31 (1·14-4·70)	0·02
**Clinical acute phase deficits**						
Stroke severity (per point on admissionNIHSS)	21/757	1·09 (1·01-1·18)	0·03	34/650	1·07 (1·00-1·14)	0·05
Admission NIHSS ≥3	21/757	2·50 (0·91-6·83)	0·07	34/650	2·49 (1·12-5·53)	0·02
Barthel Index (per point)	21/755	0·97 (0·96-0·98)	<0·0001	34/648	0·98 (0·97-1·00)	0·009
Delirious symptoms (per point on DRS)	21/757	1·30 (1·14-1·48)	<0·0001	34/650	1·01 (0·80-1·27)	0·95
Acute phase cognitive function (per point on MoCA)	15/669	0·80 (0·71-0·90)	0·0002	26/581	0·86 (0·78-0·94)	0·001
Acute phase cognitive impairment*[1]	20/733	5·38 (1·24-23·43)	0·02	29/632	7·07 (2·12-23·56)	0·001
**Cardiovascular risk factors**						
Hypertension	21/757	0·77 (0·22-2·65)	0·67	34/650	1·09 (0·38-3·16)	0·87
Diabetes mellitus	21/757	2·16 (0·90-5·17)	0·08	34/650	2·00 (0·98-4·10)	0·06
Current smoking	21/757	0·00 (0·00-Inf)	0·99	34/650	1·80 (0·71-4·53)	0·21
Dyslipidaemia	21/757	0·86 (0·35-2·09)	0·78	34/650	1·53 (0·77-3·04)	0·22
Atrial fibrillation	21/757	4·01 (1·68-9·59)	0·002	34/650	1·34 (0·63-2·84)	0·45
Prior history of stroke	21/757	2·45 (0·93-6·43)	0·07	34/650	1·46 (0·56-3·82)	0·44
Ischaemic heart disease	21/757	1·67 (0·60-4·65)	0·32	34/650	1·60 (0·68-3·76)	0·28
BMI, kg/m2	21/757	1·00 (0·89-1·13)	0·97	34/650	0·99 (0·90-1·09)	0·88
SBP, mmHg	21/752	1·02 (0·99-1·04)	0·18	34/645	0·99 (0·97-1·01)	0·48
DBP, mmHg	21/752	1·00 (0·97-1·04)	0·80	34/645	1·00 (0·98-1·03)	0·73
HbA_{1c}, %	20/703	1·07 (0·90-1·27)	0·47	32/602	1·06 (0·93-1·22)	0·35
LDL-C, mg/dL	20/731	0·99 (0·98-1·00)	0·28	33/627	1·01 (1·00-1·01)	0·16
HDL-C, mg/dL	20/726	0·98 (0·94-1·01)	0·23	32/622	0·98 (0·97-1·01)	0·17
Triglycerides, mg/dL	20/708	1·00 (0·99-1·01)	0·88	32/605	1·00 (1·00-1·01)	0·01
**Metabolic syndrome components*[2]**						
Abdominal obesity	16/716	0·56 (0·20-1·53)	0·26	32/620	1·26 (0·58-2·69)	0·56
Elevated triglycerides	20/708	0·90 (0·33-2·48)	0·84	32/605	2·01 (0·97-4·16)	0·06
Reduced HDL-C	20/726	2·20 (0·90-5·38)	0·08	32/622	2·81 (1·37-5·76)	0·005
Elevated blood pressure	21/756	0·92 (0·12-6·97)	0·94	34/649	0·31 (0·10-0·90)	0·03
Prediabetes/Diabetes mellitus	20/712	2·02 (0·73-5·57)	0·17	33/610	2·21 (1·00-4·91)	0·05
≥ 3 of the above criteria present	21/757	0·97 (0·41-2·31)	0·95	34/650	3·33 (1·46- 7·57)	0·004
Per count of components increase	21/757	1·06 (0·74-1·52)	0·76	34/650	1·43 (1·08-1·90)	0·01
**Index stroke classification**						
Haemorrhagic stroke	21/757	1·59 (0·21-11·95)	0·65	34/650	4·35 (1·30-14·59)	0·02
**Acute stroke treatment**						
Any reperfusion therapy (IVT and/or EVT)	21/757	0·41 (0·14-1·20)	0·10	34/650	0·25 (0·10-0·67)	0·006

**Neuroimaging parameters**						
Normalised brain volume (per SD)	19/677	0·54 (0·30-0·96)	0·04	31/587	0·67 (0·43-1·06)	0·09
Normalised stroke lesion volume (per SD)	19/677	1·42 (1·06-1·90)	0·02	31/587	1·01 (0·66-1·54)	0·98
Total SVD score (per SD)	20/686	1·51 (0·99-2·31)	0·06	31/595	1·12 (0·76-1·64)	0·57
Lacune count (per SD)	21/691	1·30 (1·10-1·54)	0·002	32/599	1·44 (1·14-1·81)	0·002
**Presence of ≥3 lacunes**	21/691	5·07 (1·11-23·09)	0·04	32/599	11·24 (3·08-41·09)	0·0002
Normalised WMH volume (per SD)	19/675	1·30 (0·96-1·76)	0·09	29/585	1·50 (1·17-1·91)	0·001
CMB count (per SD)	20/686	1·11 (0·82-1·49)	0·50	31/595	1·22 (1·00-1·48)	0·04
PVS grade (per SD)	21/690	1·46 (1·00-2·14)	0·05	32/598	1·16 (0·83-1·62)	0·39
Mean skeletonised mean diffusivity (per SD)	17/647	1·84 (1·20-2·83)	0·005	28/563	1·98 (1·33-2·95)	0·0008
**Genetic risk factors**						
APOE genotype						
1 ε4 allele	16/621	1·94 (0·65-5·74)	0·23	27/536	0·80 (0·29-2·19)	0·66
2 ε4 alleles	16/621	9·33 (1·18-73·67)	0·03	27/536	3·43 (0·46-25·86)	0·23
**Pre-stroke clinical/cognitive function**						
mRS before stroke	21/757	1·10 (0·64-1·86)	0·76	34/650	1·19 (0·76-1·85)	0·45
IQCODE score	19/704	1·04 (0·91-1·20)	0·54	30/606	1·10 (0·98-1·25)	0·11

Cox proportional hazards regression models for the association between risk factors and early-onset (left) and delayed-onset PSD (right), adjusting for age, sex, education, admission NIHSS, and stroke recurrence. Recurrent stroke was included as a time-dependent variable as described in the **Supplementary Methods**. APOE=apolipoprotein E. BMI=body-mass index. CMB = cerebral microbleed. DBP = diastolic blood pressure. EVT=endovascular thrombectomy. HbA_{1c}=glycated haemoglobin. HDL-C=high-density lipoprotein cholesterol. IQCODE=Informant Questionnaire on Cognitive Decline in the Elderly. IVT=intravenous thrombolysis. LDL-C=low-density lipoprotein cholesterol. MoCA=Montreal Cognitive Assessment. mRS=Modified Rankin Scale. NIHSS=National Institutes of Health Stroke Scale. PVS = perivascular space. SBP = systolic blood pressure. SD = standard deviation. SVD = small vessel disease. WMH = white matter hyperintensity.

*[1] MoCA <26 or mini-mental state examination <27 when MoCA was not available (n=73).

*[2] Defined according to Alberti et al.[2]

Table S14: Sensitivity analysis additionally adjusting the 5-year PSD analyses for acute phase cognitive impairment

	Cases/N	HR (95% CI)	P-value
**Sociodemographic factors**			
Age (per year)	49/683	1·11 (1·07-1·15)	<0·0001
Age ≥74 years	49/683	3·62 (1·97-6·65)	<0·0001
Female sex	49/683	0·65 (0·33-1·27)	0·20
Education (per year)	49/683	0·90 (0·81-0·99)	0·03
Education ≤12 years	49/683	1·56 (0·85-2·87)	0·15
**Clinical acute phase deficits**			
Stroke severity (per point on admission NHSS)	49/683	1·07 (1·02-1·13)	0·006
Admission NIHSS ≥3	49/683	2·43 (1·28-4·62)	0·007
Barthel Index (per point)	49/682	0·98 (0·97-0·99)	0·002
Delirious symptoms (per point on DRS)	49/683	1·14 (1·01-1·28)	0·03
**Cardiovascular risk factors**			
Hypertension	49/683	1·06 (0·44-2·54)	0·90
Diabetes mellitus	49/683	2·71 (1·52-4·85)	0·0007
Dyslipidaemia	49/683	1·24 (0·70-2·22)	0·46
Current smoking	49/683	0·87 (0·36-2·14)	0·77
Regular alcohol consumption	49/683	0·70 (0·37-1·32)	0·27
Atrial fibrillation	49/683	2·22 (1·24-3·96)	0·007
Prior stroke	49/683	1·95 (0·98-3·289)	0·06
Ischaemic heart disease	49/683	1·65 (0·83-3·25)	0·15
BMI (kg/m^2)	49/683	1·02 (0·94-1·10)	0·71
SBP (mmHg)	49/678	1·00 (0·98-1·02)	0·99
DBP (mmHg)	49/678	1·01 (0·99-1·03)	0·43
HbA_{1c} (%)	46/635	1·09 (0·98-1·20)	0·12
LDL-C (mg/dL)	47/661	1·00 (0·99-1·01)	0·53
HDL-C (mg/dL)	46/656	0·97 (0·95-1·00)	0·02
Triglycerides (mg/dL)	46/639	1·00 (1·00-1·01)	0·06
**Metabolic syndrome components***			
Abdominal obesity	42/646	0·87 (0·46-1·66)	0·67
Elevated triglycerides	46/639	1·55 (0·82-2·95)	0·18
Reduced HDL-C	46/656	2·50 (1·38-4·56)	0·003
Elevated blood pressure	49/682	0·76 (0·27-2·16)	0·60
Prediabetes or diabetes mellitus	47/643	2·82 (1·37-5·84)	0·005
Metabolic syndrome (≥3 of the above components present)	49/683	2·14 (1·16-3·96)	0·02
Per count of components increase	49/683	1·34 (1·05-1·71)	0·02
**Index stroke classification**			
Haemorrhagic stroke	49/683	2·34 (0·83-6·60)	0·11
**Acute stroke treatment**			
Any acute reperfusion treatment (IVT and/or EVT)	49/683	0·49 (0·23-1·06)	0·07
**Neuroimaging parameters**			
Normalised brain volume (per SD)	45/613	0·61 (0·41-0·93)	0·02
Normalised infarct volume (per SD)	45/613	1·09 (0·82-1·45)	0·54
Total SVD score (per SD)	46/621	1·27 (0·94-1·72)	0·12
Lacune count (per SD)	48/626	1·32 (1·16-1·50)	<0·0001
Presence of ≥3 lacunes	48/626	10·33 (3·90-27·40)	<0·0001
Normalised WMH volume (per SD)	43/613	1·45 (1·20-1·76)	<0·0001
CMB count (per SD)	46/621	1·12 (0·95-1·32)	0·17
PVS grade (per SD)	48/625	1·23 (0·94-1·60)	0·13
Mean skeletonised mean diffusivity (per SD)	41/587	1·95 (1·43-2·67)	<0·0001
**Genetic risk factors**			
**APOE genotype**			
0 ε4 alleles	40/557	ref	ref
1 ε4 allele	40/557	0·91 (0·42-2·00)	0·82
2 ε4 alleles	40/557	9·51 (1·08-84·04)	0·04
**Pre-stroke clinical/cognitive function**			
mRS before stroke	49/683	0·88 (0·58-1·33)	0·55
IQCODE score	43/633	1·05 (0·96-1·16)	0·29
**Recurrent events**			

Stroke recurrence	49/732	3·19 (1·54-6·64)	0·002

Cox proportional hazards regression models for the association between risk factors and post-stroke dementia, adjusted for age, sex, education, admission NIHSS, and acute phase cognitive impairment (MoCA <26 or MMSE <27 [n=73]). Recurrent stroke was included as a time-dependent covariable as described in the **Supplementary Methods**. APOE=apolipoprotein E. BMI=body-mass index. CMB = cerebral microbleed. DBP = diastolic blood pressure. EVT=Endovascular thrombectomy. HbA_{1c}=glycated haemoglobin. HDL-C=high-density lipoprotein cholesterol. IQCODE=Informant Questionnaire on Cognitive Decline in the Elderly. IVT=Intravenous thrombolysis. LDL-C=low-density lipoprotein cholesterol. MoCA=Montreal Cognitive Assessment. mRS=Modified Rankin Scale. NIHSS=National Institutes of Health Stroke Scale. PVS = perivascular space. SBP = systolic blood pressure. SD = standard deviation. SVD = small vessel disease. WMH = white matter hyperintensity.

* Defined according to Alberti et al.[2]

Table S15: Sensitivity analysis adjusting the analysis split by post-stroke time period for age, sex, education, NIHSS, and acute phase cognitive impairment

	Dementia risk 3-6 months			*Dementia risk > 6 months*		
**Risk factors**	**Cases/N**	**HR (95% CI)**	**P-value**	**Cases/N**	**HR (95% CI)**	**P-value**
**Sociodemographic factors**						
Age (per year)	20/683	1·17 (1·09- 1·25)	<0·0001	29/599	1·08 (1·03-1·13)	0·002
Age ≥74 years	20/683	6·50 (2·16-19·53)	<0·0001	29/599	2·61 (1·23-5·52)	0·01
Female sex	20/683	0·76 (0·29-2·00)	0·58	29/599	0·58 (0·25-1·34)	0·20
Education (per year)	20/683	0·91 (0·79-1·04)	0·18	29/599	0·88 (0·78-1·00)	0·06
Education ≤12 years	20/683	1·42 (0·58-3·52)	0·44	29/599	1·66 (0·76-3·63)	0·20
**Clinical acute phase deficits**						
Stroke severity (per point on admission NIHSS)	20/683	1·09 (1·01-1·18)	0·03	29/599	1·06 (0·99-1·14)	0·08
Admission NIHSS ≥3	20/683	2·74 (0·99-7·58)	0·05	29/599	2·23 (0·98-5·09)	0·06
Barthel Index (per point)	20/682	0·97 (0·96-0·99)	0·001	29/598	0·99 (0·98-1·00)	0·22
Delirious symptoms (per point on DRS)	20/683	1·28 (1·12-1·46)	0·0003	29/599	0·94 (0·72-1·23)	0·66
**Vascular risk factors**						
Hypertension	20/683	0·65 (0·18-2·35)	0·51	29/599	1·44 (0·42-4·89)	0·56
Diabetes mellitus	20/683	2·70 (1·10-6·63)	0·03	29/599	2·49 (1·16-5·38)	0·02
Dyslipidaemia	20/683	0·78 (0·31-1·96)	0·60	29/599	1·877 (0·83-3·77)	0·14
Current smoking	20/683	0·00 (0·00-Inf)	1·00	29/599	1·47 (0·56-3·86)	0·43
Regular alcohol consumption	20/683	0·41 (0·16-1·04)	0·06	29/599	1·15 (0·45-2·89)	0·77
Atrial fibrillation	20/683	3·42 (1·40-8·35)	0·007	29/599	1·62 (0·72-3·60)	0·24
Prior history of stroke	20/683	2·40 (0·90-6·39)	0·08	29/599	1·57 (0·58-4·20)	0·37
Ischaemic heart disease	20/683	1·33 (0·44-4·39)	0·61	29/599	1·84 (0·78-4·38)	0·17
BMI, kg/m2	20/683	1·03 (0·91-1·17)	0·65	29/599	1·00 (0·90-1·10)	0·97
SBP, mmHg	20/678	1·01 (0·99-1·04)	0·34	29/594	0·99 (0·97-1·01)	0·44
DBP, mmHg	20/678	1·01 (0·97-1·05)	0·57	29/594	1·01 (0·98-1·04)	0·59
HbA_{1c}, %	19/635	1·11 (0·94-1·32)	0·22	27/555	1·07 (0·92-1·24)	0·36
LDL-C, mg/dL	19/661	0·99 (0·98-1·01)	0·31	28/580	1·01 (1·00-1·02)	0·12
HDL-C, mg/dL	19/656	0·97 (0·93-1·00)	0·08	27/575	0·98 (0·95-1·01)	0·16
Triglycerides, mg/dL	19/639	1·00 (1·00-1·01)	0·50	27/559	1·00 (1·00-1·01)	0·10
**Metabolic syndrome components***						
Abdominal obesity	15/646	0·50 (0·17-1·48)	0·21	27/571	1·08 (0·49-2·44)	0·85
Elevated triglycerides	19/639	1·22 (0·43-3·47)	0·70	27/559	1·71 (0·75-3·89)	0·20
Reduced HDL-C	19/656	2·973 (1·05-7·14)	0·04	27/575	2·42 (1·12-5·24)	0·02
Elevated blood pressure	20/682	1·17 (0·15-8·93)	0·88	29/598	0·65 (0·19-2·24)	0·49
Prediabetes/Diabetes mellitus	19/643	2·64 (0·86-8·17)	0·09	28/562	2·82 (1·10-7·20)	0·03
Metabolic syndrome (≥ 3 of the above components present)	20/683	1·18 (0·49-2·88)	0·71	29/599	3·32 (1·37-8·07)	0·008
Per count of components increase	20/683	1·20 (0·81-1·78)	0·36	29/599	1·41 (1·03-1·93)	0·03
**Index stroke classification**						
Haemorrhagic stroke	20/683	1·33 (0·17-10·20)	0·78	29/599	3·25 (0·96-10·98)	0·06
**Acute stroke treatment**						
**Any reperfusion therapy (IVT and/or EVT)**	20/683	0·61 (0·19-2·01)	0·42	29/599	0·41 (0·15-1·15)	0·09

**Neuroimaging parameters**						
Normalised brain volume (per SD)	18/613	0·72 (0·37-1·341)	0·34	27/542	0·58 (0·35-0·97)	0·04
Normalised infarct volume (per SD)	18/613	1·39 (0·99-1·93)	0·06	27/542	0·74 (0·39-1·40)	0·36
Total SVD score (per SD)	19/621	1·42 (0·89-2·78)	0·14	27/549	1·15 (0·76-1·74)	0·51
Lacune count (per SD)	20/626	1·33 (1·12-1·58)	0·001	28/553	1·43 (1·14-1·78)	0·002
Presence of ≥3 lacunes	20/626	6·81 (1·52-30·52)	0·01	28/553	17·70 (4·77-66·06)	<0·0001
Normalised WMH volume (per SD)	19/613	1·31 (0·95-1·80)	0·09	27/542	1·59 (1·24-2·03)	0·0002
CMB count (per SD)	18/621	1·03 (0·76-1·41)	0·83	28/549	1·15 (0·96-1·38)	0·14
PVS grade (per SD)	20/625	1·30 (0·87-1·95)	0·20	28/552	1·17 (0·81-1·68)	0·41
Mean skeletonised mean diffusivity (per SD)	16/587	1·71 (1·01-2·89)	0·05	25/521	2·08 (1·40-3·11)	0·0003
**APOE genotype**						
1 ε4 allele	15/557	1·65 (0·54-5·00)	0·38	25/494	0·59 (0·20-1·73)	0·37
2 ε4 alleles	15/557	32·70 (3·46-309·76)	0·002	25/494	0·00 (0·00-Inf)	1·00
**Pre-stroke clinical/cognitive function**						
mRS before stroke	20/683	0·71 (0·37-1·36)	0·30	29/599	1·01 (0·60-1·70)	0·97
IQCODE score	18/633	1·05 (0·95-1·16)	0·37	25/558	1·08 (0·94-1·23)	0·27
**Recurrent events**						
Stroke recurrence	20/732	0·69 (0·09-5·18)	0·72	29/631	5·78 (2·52-13·26)	<0·0001

Cox proportional hazards regression models for the association between risk factors and early-onset (left) and delayed-onset PSD (right), adjusted for age, sex, education, admission NIHSS, and acute phase cognitive impairment (MoCA <26 or MMSE <27 [n=73]). Recurrent stroke was included as a time-dependent covariable as described in the **Supplementary Methods**. APOE=apolipoprotein E. BMI=body-mass index. CMB = cerebral microbleed. DBP = diastolic blood pressure. EVT=Endovascular thrombectomy. HbA_{1c}=glycated haemoglobin. HDL-C=high-density lipoprotein cholesterol. IQCODE=Informant Questionnaire on Cognitive Decline in the Elderly. IVT=Intravenous thrombolysis. LDL-C=low-density lipoprotein cholesterol. MoCA=Montreal Cognitive Assessment. mRS=Modified Rankin Scale. NIHSS=National Institutes of Health Stroke Scale. PVS = perivascular space. SBP = systolic blood pressure. SD = standard deviation. SVD = small vessel disease. WMH = white matter hyperintensity.
* Defined according to Alberti et al.[2]

Table S16: Sensitivity analysis using the cut-off of 12 months for early- vs. delayed-onset dementia

	Early-onset dementia risk (3-12 months)			*Delayed-onset dementia risk (>12 months)*		
**Baseline risk factor**	**Cases/N**	**HR (95% CI)**	**P-value**	**Cases/N**	**HR (95% CI)**	**P-value**
Age (per year)	31/706	1·17 (1·11-1·23)	< 0·0001	24/589	1·09 (1·04- 1·15)	0·0006
Age ≥ 74	31/706	6·68 (2·87-15·56)	< 0·0001	24/589	3·30 (1·45-7·50)	0·004
Female sex	31/706	0·34 (0·14-0·79)	0·01	24/589	0·69 (0·29-1·64)	0·40
Education (per year)	31/706	0·92 (0·82-1·03)	0·13	24/589	0·78 (0·67-0·90)	0·001
Education ≤ 12	31/706	1·43 (0·69-2·96)	0·33	24/589	2·70(1·16-6·30)	0·02
**Clinical/cognitive acute phase deficits**						
Stroke severity (per point on admission NIHSS)	31/706	1·07 (1·00-1·14)	0·05	24/589	1·08 (1·01-1·16)	0·02
Admission NIHSS ≥3	31/706	2·86 (1·23-6·65)	0·01	24/589	2·45 (0·96-6·21)	0·06
Barthel Index (per point)	31/704	0·97 (0·96-0·99)	< 0·0001	24/587	0·99 (0·97-1·00)	0·12
Delirious symptoms (per point on DRS)	31/706	1·25 (1·11-1·41)	0·0002	24/589	0·92 (0·65-1·30)	0·64
Acute phase cognitive function (per point on MoCA)	24/625	0·82 (0·74-0·90)	< 0·0001	17/527	0·84 (0·75-0·94)	0·003
Acute phase cognitive impairment*[1]	30/683	4·44 (1·31-15·07)	0·02	19/572	8·37 (1·89-36·94)	0·005
**Cardiovascular risk factors**						
Hypertension	31/706	0·70 (0·26-1·89)	0·49	24/589	1·84 (0·42-8·02)	0·42
Diabetes mellitus	31/706	2·02 (0·97-4·20)	0·06	24/589	2·48 (1·08-5·72)	0·03
Dyslipidaemia	31/706	1·32 (0·65-2·70)	0·44	24/589	1·42 (0·63-3·23)	0·40
Current smoking	31/706	0·24 (0·03-1·83)	0·17	24/589	1·76 (0·61-5·13)	0·30
Regular alcohol consumption	31/706	0·54 (0·25-1·16)	0·11	24/589	1·29 (0·47-3·54)	0·62
Atrial fibrillation	31/706	2·56 (1·26-5·20)	0·009	24/589	1·32 (0·53-3·27)	0·54
Prior history of stroke	31/706	2·29 (0·98-5·35)	0·06	24/589	1·64 (0·55-4·89)	0·37
Ischaemic heart disease	31/706	2·21 (0·98-5·02)	0·06	24/589	1·52 (0·51-4·53)	0·45
BMI (kg/m^2)	31/706	0·99 (0·89-1·10)	0.86	24/589	0·99 (0·88-1·11)	0·86
Systolic blood pressure (mmHg)	31/701	1·01 (0·99-1·03)	0·18	24/584	0·99 (0·96-1·01)	0·22
Diastolic blood pressure (mmHg)	31/701	1·01 (0·98-1·04)	0·36	24/584	0·99 (0·96-1·03)	0·70
HbA_{1c} (%)	30/658	1·05 (0·87-1·27)	0·60	22/546	1·07 (0·94-1·21)	0·32
LDL cholesterol (mg/dL)	30/684	1·00 (0·99-1·01)	0·89	23/570	1·00 (0·99-1·01)	0·68
HDL cholesterol (mg/dL)	30/679	0·99 (0·96-1·02)	0·52	22/565	0·96 (0·93-1·00)	0·05
Triglycerides (mg/dL)	30/662	1·00 (0·99-1·00)	0·81	22/551	1·00 (1·00-1·01)	0·004
**Metabolic syndrome components*[2]**						
Abdominal obesity	25/666	0·60 (0·26-1·40)	0·24	23/560	1·70 (0·66-4·35)	0·27
Elevated triglycerides	30/662	0·84 (0·36-1·96)	0·69	22/551	3·25 (1·34-7·88)	0·009
Reduced HDL cholesterol	30/679	1·77 (0·83-3·78)	0·14	22/565	4·22 (1·71-10·42)	0·002
Elevated blood pressure	31/705	0·42 (0·12-1·43)	0·16	24/588	0·58 (0·13-2·57)	0·47
Prediabetes/Diabetes mellitus	30/666	1·94 (0·86-4·38)	0·11	23/552	2·26 (0·84-6·05)	0·10
Metabolic syndrome (≥3 of the above components present)	31/706	1·17 (0·57-2·40)	0·67	24/589	5·13 (1·70-15·50)	0·004
Per count of components increase	31/706	1·03 (0·76-1·41)	0·83	24/589	1·67 (1·19-2·35)	0·003
**Index stroke classification**						
Haemorrhagic stroke	31/706	1·80 (0·42-7·66)	0·42	24/589	4·71 (1·08-20·59)	0·04
**Acute stroke treatment**						
Any reperfusion therapy (IVT and/or EVT)	31/706	0·34 (0·12-0·99)	0·05	24/589	0·35 (0·11-1·09)	0·07

**Neuroimaging parameters**						
Normalised brain volume (per SD)	27/634	0·68 (0·40-1·15)	0·15	24/534	0·54 (0·32-0·92)	0·02
Normalised infarct volume (per SD)	27/634	1·25 (0·92-1·71)	0·16	24/534	1·04 (0·68-1·57)	0·87
Total SVD score (per SD)	29/642	1·36 (0·92-2·01)	0·12	22/541	1·06 (0·65-1·71)	0·82
Lacune count (per SD)	30/647	1·35 (1·16-1·57)	< 0·0001	23/544	1·61 (1·18-2·19)	0·002
Presence of ≥3 lacunes	30/647	9·31 (2·72-31·85)	0·0004	23/544	16·32 (3·47-76·67)	0·0004
Normalised WMH volume (per SD)	27/633	1·45 (1·14-1·84)	0·002	21/534	1·38 (0·96-1·98)	0·08
Cerebral microbleed count (per SD)	29/642	1·12 (0·89-1·42)	0·33	22/541	1·20 (0·96-1·50)	0·11
Perivascular space grade (per SD)	30/646	1·36 (0·97-1·90)	0·07	23/543	1·07 (0·70-1·64)	0·74
Mean skeletonised mean diffusivity (per SD)	25/606	1·87 (1·24-2·82)	0·003	20/512	1·98 (1·22-3·21)	0·006
**Genetic risk factors**						
APOE genotype						
0 ε4 alleles	ref	ref	··	ref	ref	··
1 ε4 allele	25/576	1·34 (0·54-3·30)	0·53	18/482	0·60 (0·16-2·22)	0·44
2 ε4 alleles	25/576	4·21 (0·55-32·36)	0·16	18/482	3·88 (0·49-30·55)	0·20
**Pre-stroke clinical/cognitive function**						
mRS before stroke	31/706	0·90 (0·56-1·45)	0·67	24/589	1·31 (0·81-2·13)	0·27
IQCODE score	29/655	1·04 (0·95-1·15)	0·12	20/548	1·16 (1·01-1·34)	0·03
**Recurrent events**						
Stroke recurrence	31/757	1·84 (0·64-5·31)	0·26	24/615	3·41 (1·25-9·27)	0·02

Cox proportional hazards regression models for the association between risk factors and early-onset (left) and delayed-onset PSD (right), when setting the cut-off at 1 year instead of 6 months post stroke. Recurrent stroke was included as a time-dependent variable as described in the **Supplementary Methods**.

APOE=apolipoprotein E. BMI=body-mass index. DRS=Delirious rating scale. EVT=Endovascular thrombectomy. HbA_{1c}=glycated haemoglobin. HDL=high-density lipoprotein. IQCODE=Informant Questionnaire on Cognitive Decline in the Elderly. IVT=Intravenous thrombolysis. LDL=low-density lipoprotein. MoCA=Montreal Cognitive Assessment. mRS=Modified Rankin Scale. NIHSS=National Institutes of Health Stroke Scale. WMH=white matter hyperintensity.

*[1] MoCA <26 or mini-mental state examination <27 when MoCA was not available (n=73).

*[2] Defined according to Alberti et al.[2]

Table S17: Sensitivity analysis with multiple imputation of the dementia onset date

Risk Factor	Cases/N	HR (95% CI)	P-Value
**Sociodemographic factors**			
Age (per year)	55/706	1·13 (1·09-1·18)	<0·0001
Age ≥74	55/706	4·74 (2·66-8·47)	<0·0001
Female sex	55/706	0·49 (0·26-0·92)	0·03
Education (per year)	55/706	0·87 (0·79-0·95)	0·004
Education ≤12	55/706	1·83 (1·05-3·21)	0·04
**Clinical acute phase deficits**			
Stroke severity (per point on admission NHSS)	55/706	1·07 (1·02-1·13)	0·005
Admission NIHSS ≥3	55/706	2·72 (1·45-5·10)	0·003
Barthel Index (per point)	55/704	0·98 (0·97-0·99)	0·0001
Delirious symptoms (per point on DRS)	55/706	1·17 (1·04-1·30)	0·009
Acute phase cognitive function (per point on MoCA)	41/625	0·83 (0·77-0·90)	<0·0001
Acute phase cognitive impairment*[1]	49/683	5·89 (2·30-15·10)	0·0006
**Vascular risk factors**			
Hypertension	55/706	1·05 (0·47-2·35)	0·91
Diabetes mellitus	55/706	2·26 (1·30-3·92)	0·006
Dyslipidaemia	55/706	1·35 (0·79-2·31)	0·28
Current smoking	55/706	0·84 (0·35-2·01)	0·69
Regular alcohol consumption	55/706	0·73 (0·40-1·34)	0·31
Atrial fibrillation	55/706	1·86 (1·08-3·22)	0·03
Prior stroke	55/706	2·00 (1·03-3·91)	0·05
Ischaemic heart disease	55/706	1·96 (1·03-3·75)	0·05
BMI (kg/m^2)	55/706	0·99 (0·92-1·08)	0·92
SBP (mmHg)	55/701	1·00 (0·99-1·02)	0·88
DBP (mmHg)	55/701	1·00 (0·98-1·03)	0·76
HbA1c (%)	52/658	1·06 (0·96-1·18)	0·26
LDL-C (mg/dL)	53/684	1·00 (0·99-1·01)	0·86
HDL-C (mg/dL)	52/679	0·98 (0·96-1·00)	0·08
Triglycerides (mg/dL)	52/662	1·00 (1·00-1·01)	0·04
**Metabolic syndrome components*[2]**			
Abdominal obesity	48/666	0·98 (0·53-1·81)	0·96
Elevated triglycerides	52/662	1·54 (0·86-2·75)	0·15
Reduced HDL-C	52/679	2·56 (1·46-4·51)	0·002
Elevated blood pressure	55/705	0·47 (0·18-1·22)	0·13
Prediabetes or diabetes mellitus	53/666	2·05 (1·10-3·82)	0·03
Metabolic syndrome (≥3 of the above components present)	55/706	1·99 (1·12-3·54)	0·02
Per count of components increase	55/706	1·30 (1·04-1·62)	0·02
**Index stroke classification**			
Haemorrhagic stroke	55/706	2·69 (0·96-7·55)	0·07
**Acute stroke treatment**			
Any acute reperfusion therapy (IVT and/or EVT)	55/706	0·35 (0·16-0·74)	0·009
**Neuroimaging parameters**			
Normalised brain volume (per SD)	50/634	0·60 (0·41-0·87)	0·01
Normalised infarct volume (per SD)	50/634	1·19 (0·93-1·51)	0·17
Total SVD score (per SD)	51/642	1·24 (0·92-1·66)	0·16
Lacune count (per SD)	53/647	1·36 (1·20-1·55)	<0·0001
Presence of ≥3 lacunes	53/647	11·15 (4·8-29·04)	0·001
Normalised WMH volume (per SD)	48/633	1·40 (1·16-1·70)	0·002
CMB count (per SD)	51/642	1·15 (0·98-1·36)	0·09
PVS grade (per SD)	53/646	1·19 (0·92-1·54)	0·19
Mean skeletonised mean diffusivity (per SD)	45/606	1·89 (1·39-2·57)	0·0002
**APOE genotype**			
1 ε4 allele	43/576	1·15 (0·55-2·40)	0·72
2 ε4 alleles	43/576	5·34 (1·25-22·91)	0·003
**Pre-stroke clinical/cognitive function**			
mRS before stroke	55/706	1·09 (0·78-1·54)	0·61
IQCODE score	49/655	1·07 (0·98-1·18)	0·15

Pooled results of the association between baseline risk factors and post-stroke dementia, based on 20 datasets, derived using multiple imputation for the date of dementia onset. APOE=apolipoprotein E. BMI=body-mass index. CMB = cerebral microbleed. DBP = diastolic blood pressure. HbA_{1c}=glycated haemoglobin. HDL-C=high-density lipoprotein cholesterol. IQCODE=Informant Questionnaire on Cognitive Decline in the Elderly. LDL-C=low-density lipoprotein cholesterol. MoCA=Montreal Cognitive Assessment. mRS=Modified Rankin Scale. NIHSS=National Institutes of Health Stroke Scale. PVS = perivascular space. SBP = systolic blood pressure. SD = standard deviation. SVD = small vessel disease. WMH = white matter hyperintensity.

*[1] MoCA <26 or mini-mental state examination <27 when MoCA was not available (n=73).

*[2] Defined according to Alberti et al.[2]

Table S18: Comparison of subdistribution and cause-specific hazard ratios for 5-year PSD risk

	Cases/N	Subdistribution HR (95% CI)	P-Value	P-Value PH Test	Cause-specific HR (95% CI)	P-Value	P-Value PH Test
**Sociodemographic factors**							
Age (per year)	55/706	1·13 (1·08-1·18)	<0·0001	0·21	1·13 (1·09-1·17)	<0·0001	0·09
Age ≥74	55/706	4·76 (2·65-8·55)	<0·0001	0·47	4·75 (2·66-8·49)	<0·0001	0·28
Female sex	55/706	0·40 (0·20-0·80)	0·009	0·82	0·40 (0·21-0·77)	0·006	0·75
Education (per year)	55/706	0·86 (0·77-0·95)	0·002	0·52	0·86 (0·78-0·94)	0·001	0·65
Education ≤12	55/706	1·89 (1·05-3·40)	0·03	0·63	1·88 (1·07-3·29)	0·03	0·58
**Clinical/cognitive acute phase deficits**							
Stroke severity (per point on admission NHSS)	55/706	1·12 (1·06-1·19)	0·0001	0·36	1·12 (1·06-1·18)	<0·0001	0·59
Admission NIHSS ≥3	55/706	2·68 (1·44-4·97)	0·002	0·93	2·67 (1·43-5·00)	0·002	0·93
Barthel Index (per point)	55/704	0·98 (0·97-0·99)	<0·0001	0·14	0·98 (0·97-0·99)	<0·0001	0·20
Delirious symptoms (per point on DRS)	55/706	1·15 (0·99-1·34)	0·07	0·05	1·15 (1·03-1·29)	0·01	0·03
Acute phase cognitive function (per point on MoCA)	41/625	0·84 (0·77-0·92)	<0·0001	0·26	0·84 (0·78-0·90)	<0·0001	0·29
Acute phase cognitive impairment*[1]	49/683	5·86 (2·21-15·58)	0·0004	0·90	5·91 (2·30-15·13)	0·0002	0·76
**Vascular risk factors**							
Hypertension	55/706	1·01 (0·44-2·35)	0·98	0·31	1·02 (0·45-2·28)	0·97	0·21
Diabetes mellitus	55/706	2·33 (1·38-3·94)	0·001	0·60	2·33 (1·34-4·05)	0·003	0·61
Dyslipidaemia	55/706	1·32 (0·75-2·30)	0·34	0·46	1·31 (0·76-2·25)	0·32	0·44
Current smoking	55/706	0·79 (0·34-1·82)	0·58	0·06	0·79 (0·33-1·89)	0·59	0·03
Regular alcohol consumption	55/706	0·82 (0·44-1·55)	0·55	0·10	0·83 (0·45-1·52)	0·54	0·06
Atrial fibrillation	55/706	2·07 (1·19-3·61)	0·01	0·07	2·07 (1·19-3·59)	0·01	0·05
Prior stroke	55/706	1·82 (0·91-3·65)	0·09	0·11	1·82 (0·91-3·63)	0·09	0·15
Ischaemic heart disease	55/706	1·86 (0·91-3·81)	0·09	0·26	1·86 (0·96-3·57)	0·06	0·42
BMI (per unit [kg/m^2])	55/706	1·01 (0·93-1·10)	0·81	0·60	1·01 (0·94-1·09)	0·79	0·57
SBP (per mmHg)	55/701	1·00 (0·99-1·02)	0·97	0·06	1·00 (0·98-1·02)	0·97	0·08
DBP (per mmHg)	55/701	1·00 (0·98-1·02)	0·79	0·48	1·00 (0·98-1·03)	0·80	0·64
HbA_{1c} (%)	52/658	1·05 (0·97-1·12)	0·22	0·65	1·05 (0·94-1·16)	0·39	0·72
LDL-C (per 10 mg/dL)	53/684	1·00 (0·99-1·01)	0·98	0·15	1·00 (0·99-1·01)	0·97	0·11
HDL-C (per 10 mg/dL)	52/679	0·98 (0·95-1·00)	0·11	0·29	0·98 (0·96-1·00)	0·06	0·67
Triglycerides (mg/dL)	52/663	1·00 (1·00-1·01)	0·03	0·06	1·00 (1·00-1·01)	0·07	0·05
**Metabolic syndrome components*[2]**							
Abdominal obesity	48/666	1·17 (0·59-2·33)	0·66	0·06	1·17 (0·63-2·16)	0·62	0·06
Elevated triglycerides	52/663	1·49 (0·86-2·60)	0·16	0·02	1·49 (0·83-2·68)	0·18	0·03
Reduced HDL-C	52/679	2·69 (1·55-4·66)	0·0004	0·61	2·68 (1·51-4·75)	0·0007	0·52
Elevated blood pressure	55/705	0·37 (0·14-0·98)	0·04	0·57	0·37 (0·14-0·98)	0·05	0·63
Prediabetes or diabetes mellitus	53/666	2·33 (1·24-4·38)	0·009	0·64	2·32 (1·23-4·38)	0·009	0·71
Metabolic syndrome (≥3 of the above components present)	55/706	2·18 (1·23-3·86)	0·008	0·07	2·18 (1·22-3·90)	0·008	0·03
Per count of components increase	55/706	1·33 (1·07-1·65)	0·01	0·06	1·33 (1·06-1·66)	0·01	0·05

**Index stroke classification**							
Haemorrhagic stroke	55/706	2·02 (0·76-5·43)	0·16	0·60	2·02 (0·71-5·77)	0·19	0·48
**Acute stroke treatment**							
Any reperfusion therapy (IVT and/or EVT)	55/706	0·35 (0·16-0·77)	0·009	0·41	0·35 (0·16-0·75)	0·007	0·52
**Neuroimaging parameters**							
Normalised brain volume (per SD)	50/634	0·61 (0·41-0·91)	0·02	0·31	0·61 (0·42-0·90)	0·01	0·32
Normalised infarct volume (per SD)	50/634	1·19 (0·93-1·52)	0·16	0·41	1·19 (0·93-1·52)	0·17	0·32
Total SVD score (per SD)	51/642	1·17 (0·84-1·62)	0·35	0·22	1·17 (0·87-1·57)	0·31	0·24
Lacune count (per SD)	53/647	1·38 (1·27-1·50)	<0·0001	0·14	1·38 (1·21-1·57)	<0·0001	0·12
Presence of ≥3 lacunes	53/647	10·39 (5·16-20·92)	<0·0001	0·10	10·38 (3·94-27·33)	<0·0001	0·07
Normalised WMH volume (per SD)	48/633	1·37 (1·17-1·61)	0·0001	0·68	1·37 (1·14-1·66)	0·001	0·90
CMB count (per SD)	51/642	1·15 (1·04-1·27)	0·007	0·88	1·15 (0·97-1·36)	0·10	0·95
PVS grade (per SD)	53/646	1·19 (0·90-1·57)	0·22	0·22	1·19 (0·92-1·54)	0·19	0·22
Mean skeletonised mean diffusivity (per SD)	45/606	1·88 (1·37-2·58)	<0·0001	0·29	1·88 (1·39-2·54)	<0·0001	0·50
**APOE genotype**				0·93			0·86
1 ε4 allele	43/576	1·11 (0·52-2·36)	0·78		1·11 (0·53-2·32)	0·78	
2 ε4 alleles	43/576	4·94 (1·36-17·90)	0·01		4·93 (1·15-21·04)	0·03	
**Pre-stroke clinical/cognitive function**							
mRS before stroke	55/706	1·09 (0·74-1·61)	0·66	0·78	1·09 (0·77-1·55)	0·62	0·89
IQCODE score	49/655	1·07 (0·89-1·30)	0·47	0·92	1·07 (0·98-1·18)	0·13	0·53
**Recurrent events**							
Stroke recurrence	55/757	2·36 (1·16-4·83)	0·02	0·18	2·47 (1·20-5·11)	0·01	0·15

Associations between risk factors and post-stroke dementia derived by competing risk (left) and standard Cox proportional hazards models. Death was included as a competing risk. Cox proportional hazards regression models for the association between risk factors and post-stroke dementia, adjusted for age, sex, education, and admission NIHSS. The proportional hazards assumption was tested using the Grambsch and Therneau test based on Schoenfeld residuals (p-values < 0.05 suggest a potential violation). Recurrent stroke was included as a time-dependent covariable as described in the **Supplementary Methods**. APOE=apolipoprotein E. BMI=body-mass index. CMB = cerebral microbleed. DBP = diastolic blood pressure. EVT=Endovascular Thrombectomy. HbA_{1c}=glycated haemoglobin. HDL-C=high-density lipoprotein cholesterol. IQCODE=Informant Questionnaire on Cognitive Decline in the Elderly. IVT=Intravenous Thrombolysis. LDL-C=low-density lipoprotein cholesterol. MoCA=Montreal Cognitive Assessment. mRS=Modified Rankin Scale. NIHSS=National Institutes of Health Stroke Scale. PH=Proportional Hazards. PVS = perivascular space. SBP = systolic blood pressure. SD = standard deviation. SVD = small vessel disease. WMH = white matter hyperintensity.

*[1] MoCA <26 or mini-mental state examination <27 when MoCA was not available (n=73).

*[2] Defined according to Alberti et al.[2]

Table S19: Time-dependent hazard ratios for PSD for selected risk factors

Variable	Time-varying	P (TVC)	HR (95% CI) 3 months	6 months	1 year	2 years	3 years	4 years	5 years
Delirium Rating Scale	Yes	0.003	1.45 (1.25-1.68)	1.45 (1.21-1.73)	1.00 (0.72-1.39)	0.47 (0.10-2.15)	0.87 (0.70-1.07)	0.90 (0.75-1.08)	0.88 (0.72-1.06)
Currently smoking	Yes	0.02	0.00 (0.00–32.19)	0.04 (0.00–4.91)	0.63 (0.09–4.33)	1.80 (0.54–6.00)	1.39 (0.55–3.53)	1.24 (0.40–3.86)	1.23 (0.30–5.01)
Atrial fibrillation	No	0.23	..	..	..	..	..	..	..
Triglycerides	No	0.07	..	..	..	..	..	..	..
Abdominal obesity	No	0.29	..	..	..	..	..	..	..
Elevated Triglycerides	No	0.16	..	..	..	..	..	..	..
Metabolic Syndrome (≥ 3 components present)	Yes	0.01	0.70 (0.23–2.16)	0.84 (0.40–1.76)	1.86 (0.88–3.90)	3.00 (1.50–6.00)	2.62 (1.48–4.65)	2.83 (1.58–5.06)	3.14 (1.71–5.76)
Per additional MetS component	Yes	0.006	0.92 (0.55–1.55)	0.76 (0.52–1.12)	1.20 (0.63–2.27)	3.01 (1.16–7.78)	1.84 (1.38–2.44)	1.83 (1.39–2.42)	1.95 (1.45–2.63)

Associations between selected variables and PSD using flexible parametric survival models[28] to assess potential time-varying effects. Variables were selected based on evidence of a potential violation of the proportional hazards assumption (**Table S18**). For each variable, hazard ratios and 95% confidence intervals are presented at 3 months, 6 months, and 1, 2, 3, 4, and 5 years after stroke. Time-varying effects were modelled using natural splines (df = 4 for baseline hazard; df = 2 for TVC) and adjusted for age, sex, education, and admission NIHSS. The global p-value for the time-varying effect was derived from a likelihood ratio test comparing models with and without time-varying covariate terms. The time-varying HRs for Delirium Rating Scale, smoking, Metabolic Syndrome, and per additional MetS component have been plotted and are presented in **Figure S6**. MetS=Metabolic Syndrome. NIHSS=National Institutes of Health Stroke Scale. TVC=time-varying coefficient.

Table S20: Risk factors associated with PSD and PSCI showing unadjusted hazard ratios and odds ratios

		Post-stroke dementia		Post-stoke cognitive impairment	
**Risk Factor**	**Cases/N**	**HR (95% CI)**	**P-Value**	**OR (95% CI)**	**P-Value**
**Sociodemographic factors**					
Age (per year)	55/706	1·12 (1·08-1·16)	<0·0001	1·03 (1·01-1·05)	<0·0001
Age ≥74	55/706	4·74 (2·67-8·40)	<0·0001	2·15 (1·71-2·69)	<0·0001
Female sex	55/706	1·00 (0·57-1·75)	1·00	1·22 (1·03-1·44)	0·02
Education (per year)	55/706	0·87 (0·80-0·95)	0·002	0·91 (0·89-0·94)	<0·0001
Education ≤12	55/706	1·98 (1·16-3·36)	0·01	2·02 (1·69-2·41)	<0·0001
**Clinical/cognitive acute phase deficits**					
Stroke severity (per point on admission NHSS)	55/706	1·05 (1·00-1·11)	0·07	1·03 (1·02-1·05)	0·0003
Admission NIHSS ≥3	55/706	2·65 (1·48-4·75)	0·001	0·98 (0·97-0·98)	<0·0001
Barthel Index (per point)	55/704	0·97 (0·96-0·98)	<0·0001	1·07 (1·01-1·14)	0·02
Delirious symptoms (per point on DRS)	55/706	1·21 (1·08-1·36)	0·001	0·80 (0·78-0·81)	<0·0001
Acute phase cognitive function (per point on MoCA)	41/625	0·78 (0·73-0·83)	<0·0001	0·98 (0·97-0·98)	<0·0001
Acute phase cognitive impairment*[1]	49/683	9·70 (3·83-24·54)	<0·0001	3·45 (2·91-4·08)	<0·0001
**Vascular risk factors**					
Hypertension	55/706	2·16 (0·98-4·77)	0·06	1·22 (0·92-1·62)	0·16
Diabetes mellitus	55/706	2·76 (1·60-4·75)	0·0003	1·74 (1·47-2·06)	<0·0001
Dyslipidaemia	55/706	1·83 (1·08-3·12)	0·03	1·22 (1·10-1·36)	0·0002
Current smoking	55/706	0·42 (0·18-0·99)	0·05	0·96 (0·75-1·21)	0·71
Regular alcohol consumption	55/706	0·84 (0·46-1·51)	0·55	0·87 (0·71-1·05)	0·15
Atrial fibrillation	55/706	3·22 (1·88-5·53)	<0·0001	1·99 (1·63-2·43)	<0·0001
Prior stroke	55/706	2·24 (1·16-4·35)	0·02	1·61 (1·23-2·12)	0·0006
Ischaemic heart disease	55/706	2·44 (1·29-4·64)	0·006	1·87 (1·50-2·34)	<0·0001
BMI (kg/m^2)	55/706	0·96 (0·90-1·03)	0·25	1·01 (0·98-1·03)	0·59
SBP (mmHg)	55/701	1·01 (0·99-1·02)	0·30	1·00 (0·99-1·00)	0·15
DBP (mmHg)	55/701	0·99 (0·97-1·01)	0·36	0·98 (0·98-0·99)	<0·0001
HbA1c (%)	52/658	1·03 (0·94-1·12)	0·55	1·04 (1·00-1·09)	0·04
LDL-C (mg/dL)	53/684	1·00 (0·99-1·00)	0·50	1·00 (1·00-1·00)	0·14
HDL-C (mg/dL)	52/679	0·98 (0·96-1·00)	0·12	1·00 (0·99-1·00)	0·14
Triglycerides (mg/dL)	52/662	1·00 (1·00-1·00)	0·80	1·00 (1·00-1·00)	0·70
**Metabolic syndrome components*[2]**					
Abdominal obesity	48/666	1·12 (0·63-1·99)	0·70	1·27 (1·04-1·56)	0·02
Elevated triglycerides	52/662	1·06 (0·60-1·88)	0·84	1·04 (0·87-1·24)	0·64
Reduced HDL-C	52/679	2·48 (1·44-4·27)	0·001	1·25 (1·04-1·49)	0·01
Elevated blood pressure	55/705	1·31 (0·52-3·29)	0·57	0·94 (0·69-1·27)	0·67
Prediabetes or diabetes mellitus	53/666	2·51 (1·36-4·64)	0·003	1·49 (1·26-1·76)	<0·0001
Metabolic syndrome (≥3 of the above components present)	55/706	2·22 (1·27-3·91)	0·005	1·24 (1·09-1·41)	0·0008
Per count of components increase	55/706	1·30 (1·06-1·59)	0·01	1·09 (1·02-1·16)	0·010
**Index stroke classification**					
Haemorrhagic stroke	55/706	2·95 (1·06-8·17)	0·04	1·18 (0·87-1·61)	0·28
**Acute stroke treatment**					
Any reperfusion therapy (IVT and/or EVT)	55/706	0·62 (0·32-1·20)	0·15	0·79 (0·68-0·17)	0·002
**Neuroimaging parameters**				0·65 (0·59-0·73)	<0·0001
Normalised brain volume (per SD)	50/634	0·40 (0·29-0·53)	<0·0001	1·12 (1·06-1·19)	<0·0001
Normalised infarct volume (per SD)	50/634	1·11 (0·90-1·37)	0·31	1·36 (1·22-1·51)	<0·0001
Total SVD score (per SD)	51/642	1·61 (1·26-2·05)	0·0002	1·43 (1·31-1·55)	<0·0001
Lacune count (per SD)	53/647	1·34 (1·20-1·51)	<0·0001	8·85 (4·38-17·92)	<0·0001
Presence of ≥3 lacunes	53/647	8·50 (3·36-21·51)	<0·0001	1·58 (1·38-1·81)	<0·0001
Normalised WMH volume (per SD)	48/633	1·64 (1·40-1·93)	<0·0001	1·03 (0·96-1·11)	0·40
CMB count (per SD)	51/642	1·13 (0·98-1·31)	0·09	1·24 (1·18-1·31)	<0·0001
PVS grade (per SD)	53/646	1·56 (1·24-1·96)	0·0001	1·85 (1·61-2·11)	<0·0001
Mean skeletonised mean diffusivity (per SD)	45/606	2·47 (1·92-3·18)	<0·0001	0·65 (0·59-0·73)	<0·0001
**APOE genotype**					
1 ε4 allele	43/594	1·29 (0·63-2·64)	0·48	0·96 (0·81-1·12)	0·59
2 ε4 alleles	43/594	3·58 (0·85-14·97)	0·08	2·58 (1·57-4·24)	0·0002
**Pre-stroke clinical/cognitive function**					
mRS before stroke	55/706	1·42 (1·03-1·97)	0·03	1·23 (1·10-1·37)	0·0004

IQCODE score	49/655	1·15 (1·05-1·25)	0·002	1·10 (1·00-1·21)	0·05
**Recurrent events**					
Stroke recurrence	55/757	2·55 (1·25-5·22)	0·01	··	··

Associations between risk factors and post-stroke dementia (PSD, left) and post-stroke cognitive impairment (PSCI, right). Shown are the unadjusted hazard ratios and odds ratios, derived from univariable Cox proportional hazards and GEE models, respectively. Recurrent stroke was included as a time-dependent covariable as described in the **Supplementary Methods**. APOE=apolipoprotein E. BMI=body-mass index. CMB = cerebral microbleed. DBP = diastolic blood pressure. HbA_{1c}=glycated haemoglobin. HDL-C=high-density lipoprotein cholesterol. IQCODE=Informant Questionnaire on Cognitive Decline in the Elderly. LDL-C=low-density lipoprotein cholesterol. MoCA=Montreal Cognitive Assessment. mRS=Modified Rankin Scale. NIHSS=National Institutes of Health Stroke Scale. PVS = perivascular space. SBP = systolic blood pressure. SD = standard deviation. SVD = small vessel disease. WMH = white matter hyperintensity.

*[1] MoCA <26 or mini-mental state examination <27 when MoCA was not available (n=73).

*[2] Defined according to Alberti et al.[2]

Table S21: STROBE checklist for the reporting of observational studies in epidemiology

	Item No	Recommendation	Page/Location
**Title and abstract**	1	(*a*) Indicate the study's design with a commonly used term in the title or the abstract	p.1, p.3
		(*b*) Provide in the abstract an informative and balanced summary of what was done and what was found	p.3
**Introduction**			
Background/rationale	2	Explain the scientific background and rationale for the investigation being reported	pp.7-8
Objectives	3	State specific objectives, including any prespecified hypotheses	p.8
**Methods**			
Study design	4	Present key elements of study design early in the paper	p.9
Setting	5	Describe the setting, locations, and relevant dates, including periods of recruitment, exposure, follow-up, and data collection	pp.9-10
Participants	6	(*a*) Give the eligibility criteria, and the sources and methods of selection of participants. Describe methods of follow-up	pp.9-10
		(*b*) For matched studies, give matching criteria and number of exposed and unexposed	NA
Variables	7	Clearly define all outcomes, exposures, predictors, potential confounders, and effect modifiers. Give diagnostic criteria, if applicable	p.10
Data sources/ measurement	8*	For each variable of interest, give sources of data and details of methods of assessment (measurement). Describe comparability of assessment methods if there is more than one group	pp.9-10, Suppl. methods
Bias	9	Describe any efforts to address potential sources of bias	p.10
Study size	10	Explain how the study size was arrived at	p.11, Suppl. methods
Quantitative variables	11	Explain how quantitative variables were handled in the analyses. If applicable, describe which groupings were chosen and why	Suppl. methods
Statistical methods	12	(*a*) Describe all statistical methods, including those used to control for confounding	pp.10-11, Suppl. methods
		(*b*) Describe any methods used to examine subgroups and interactions	p.11
		(*c*) Explain how missing data were addressed	p.11, Suppl. methods
		(*d*) If applicable, explain how loss to follow-up was addressed	Suppl. methods
		(*e*) Describe any sensitivity analyses	pp.10-11
**Results**			
Participants	13*	(a) Report numbers of individuals at each stage of study—eg numbers potentially eligible, examined for eligibility, confirmed eligible, included in the study, completing follow-up, and analysed	p.13, Figure 1
		(b) Give reasons for non-participation at each stage	Figure 1, Table S6
		(c) Consider use of a flow diagram	Figure 1
Descriptive data	14*	(a) Give characteristics of study participants (eg demographic, clinical, social) and information on exposures and potential confounders	p.13, Table 1, Table S1
		(b) Indicate number of participants with missing data for each variable of interest	Table S1
		(c) Summarise follow-up time (eg, average and total amount)	p.13
Outcome data	15*	Report numbers of outcome events or summary measures over time	pp.13-14, Figures S1, S3, S4, S5, and S6
Main results	16	(*a*) Give unadjusted estimates and, if applicable, confounder-adjusted estimates and their precision (eg, 95% confidence interval). Make clear which confounders were adjusted for and why they were included	pp.13-14, Table 2, Suppl. Methods, Table S7, Table S20
		(*b*) Report category boundaries when continuous variables were categorised	pp.13-14, Table 2, Suppl. methods, Table S7
		(*c*) If relevant, consider translating estimates of relative risk into absolute risk for a meaningful time period	NA
Other analyses	17	Report other analyses done—eg analyses of subgroups and interactions, and sensitivity analyses	p.15, Table S8, Tables S10-S17
**Discussion**			
Key results	18	Summarise key results with reference to study objectives	p.16
Limitations	19	Discuss limitations of the study, taking into account sources of potential bias or imprecision. Discuss both direction and magnitude of any potential bias	pp.19-20
Interpretation	20	Give a cautious overall interpretation of results considering objectives, limitations, multiplicity of analyses, results from similar studies, and other relevant evidence	pp.16-19
Generalisability	21	Discuss the generalisability (external validity) of the study results	pp.19-20
**Other information**			
Funding	22	Give the source of funding and the role of the funders for the present study and, if applicable, for the original study on which the present article is based	p.21

Table S22: The banner list of DEMDAS investigators

Last name	First name	Email	Affiliation 1	Affiliation 2	Affiliation 3	Affiliation 4
Endres	Matthias	matthias.endres@charite.de	Department of Neurology with Experimental Neurology, Charité - Univeristätsmedizin Berlin, Berlin, Germany	German Center for Neurodegenerative Diseases (DZNE), Berlin 10117, Germany	Center for Stroke Research Berlin (CSB), Charité - Universitätsmedizin Berlin, Berlin, Germany	German Centre for Cardiovascular Research (DZHK), partner site Berlin, Berlin, Germany
Liman	Thomas G.	thomas.liman@charite.de	Center for Stroke Research Berlin (CSB), Charité - Universitätsmedizin Berlin	German Center for Neurodegenerative Diseases (DZNE), Berlin 10117, Germany	Department of Neurology, Carl Von Ossietzky University, Oldenburg, Germany	
Nolte	Christian H.	christian.nolte@charite.de	Department of Neurology with Experimental Neurology, Charité - Univeristätsmedizin Berlin	German Center for Neurodegenerative Diseases (DZNE), Berlin 10117, Germany	Berlin Institute of Health (BIH), Germany	
Wittenberg	Tatjana	tatjana.wittenberg@charite.de	Center for Stroke Research Berlin (CSB), Charité - Universitätsmedizin Berlin			
Scheitz	Jan F.	jan.scheitz@charite.de	Department of Neurology with Experimental Neurology, Charité - Univeristätsmedizin Berlin	Center for Stroke Research Berlin (CSB), Charité - Universitätsmedizin Berlin		
Prüß	Harald	harald.pruess@charite.de	Department of Neurology with Experimental Neurology, Charité - Univeristätsmedizin Berlin	German Center for Neurodegenerative Diseases (DZNE), Berlin 10117, Germany		

Sperber	Pia Sophie	pia.sperber@charite.de	Center for Stroke Research Berlin (CSB), Charité - Universitätsmedizin Berlin	Department of Neurology with Experimental Neurology, Charité - Univeristätsmedizin Berlin		
Nave	Alexander H.	alexander-heinrich.nave@charite.de	Department of Neurology with Experimental Neurology, Charité - Universitätsmedizin Berlin	Center for Stroke Research Berlin (CSB), Charité - Universitätsmedizin Berlin	Berlin Institute of Health (BIH), Germany	
Kufner Ibaroule	Anna	anna.kufner@charite.de	Department of Neurology with Experimental Neurology, Charité - Universitätsmedizin Berlin	Center for Stroke Research Berlin (CSB), Charité - Universitätsmedizin Berlin	Berlin Institute of Health (BIH), Germany	
Kerti	Lucia	lucia.kerti@charite.de	Center for Stroke Research Berlin (CSB), Charité - Universitätsmedizin Berlin	German Center for Neurodegenerative Diseases (DZNE), Berlin 10117, Germany		
Petzold	Gabor C.	Gabor.Petzold@dzne.de	German Center for Neurodegenerative Diseases (DZNE), Bonn 53127, Germany	Division of Vascular Neurology, Department of Neurology, University Hospital Bonn, Bonn 53127, Germany		
Bode	Felix	felix.bode@ukbonn.de	German Center for Neurodegenerative Diseases (DZNE), Bonn 53127, Germany	Division of Vascular Neurology, Department of Neurology, University Hospital Bonn, Bonn 53127, Germany		
Stösser	Sebastian	sebastian.stoesser@ukbonn.de	German Center for Neurodegenerative Diseases (DZNE), Bonn 53127, Germany	Division of Vascular Neurology, Department of Neurology, University Hospital Bonn, Bonn 53127, Germany		

Kindler	Christine	christine.kindler @ukbonn.de	German Center for Neurodegenerative Diseases (DZNE), Bonn 53127, Germany	Division of Vascular Neurology, Department of Neurology, University Hospital Bonn, Bonn 53127, Germany		
Meißner	Julius N.	julius.meissner@ukbonn.de	German Center for Neurodegenerative Diseases (DZNE), Bonn 53127, Germany	Division of Vascular Neurology, Department of Neurology, University Hospital Bonn, Bonn 53127, Germany		
Ebrahimi	Taraneh	taraneh.ebrahimi@ukbonn.de	Division of Vascular Neurology, Department of Neurology, University Hospital Bonn, Bonn 53127, Germany			
Nordsiek	Julia	julia.nordsiek@ukbonn.de	German Center for Neurodegenerative Diseases (DZNE), Bonn 53127, Germany	Division of Vascular Neurology, Department of Neurology, University Hospital Bonn, Bonn 53127, Germany		
Beckonert	Niklas	niklas.beckonert@ukbonn.de	Division of Vascular Neurology, Department of Neurology, University Hospital Bonn, Bonn 53127, Germany			
Zerr	Inga	ingazerr@med.uni-goettingen.de	Department of Neurology, University Medical Center Göttingen, Göttingen 37075, Germany	German Center for Neurodegenerative Diseases (DZNE), Göttingen 37075, Germany		
Hermann	Peter	peter.hermann@med.uni-goettingen.de	Department of Neurology, University Medical Center Göttingen, Göttingen 37075, Germany			

Schmitz	Matthias	matthias.schmitz@med.uni-goettingen.de	Department of Neurology, University Medical Center Göttingen, Göttingen 37075, Germany			
Goebel	Stefan	stefan.goebel@med.uni-goettingen.de	Department of Neurology, University Medical Center Göttingen, Göttingen 37075, Germany			
Bunck	Timothy	timothy.bunck@med.uni-goettingen.de	Department of Neurology, University Medical Center Göttingen, Göttingen 37075, Germany			
Schütte-Schmidt	Julia	julia.schuette@med.uni-goettingen.de	Department of Neurology, University Medical Center Göttingen, Göttingen 37075, Germany			
Nuhn	Sabine	sabine.nuhn@med.uni-goettingen.de	Department of Neurology, University Medical Center Göttingen, Göttingen 37075, Germany			
Volpers	Corinna	corinna.volpers@med.uni-goettingen.de	Department of Neurology, University Medical Center Göttingen, Göttingen 37075, Germany			
Dechent	Peter	peter.dechent@med.uni-goettingen.de	Department of Cognitive Neurology, University Medical Center Göttingen, Göttingen 37075, Germany			

Bähr	Mathias	mbaehr@gwdg.de	Department of Neurology, University Medical Center Göttingen, Göttingen 37075, Germany	German Center for Neurodegenerative Diseases (DZNE), Göttingen 37075, Germany	Cluster of Excellence Nanoscale Microscopy and Molecular Physiology of the Brain (CNMPB), Göttingen, Germany	
Görtler	Michael	michael.goertler@med.ovgu.de	Department of Neurology, University Hospital, Otto-von-Guericke University Magdeburg, Magdeburg 39120, Germany	German Center for Neurodegenerative Diseases (DZNE), Magdeburg 39120, Germany		
Glanz	Wenzel	wenzel.glanz@dzne.de	Department of Neurology, University Hospital, Otto-von-Guericke University Magdeburg, Magdeburg 39120, Germany	German Center for Neurodegenerative Diseases (DZNE), Magdeburg 39120, Germany		
Dichgans	Martin	martin.dichgans@med.uni-muenchen.de	Institute for Stroke and Dementia Research (ISD), University Hospital, LMU Munich, 81377 Munich, Germany	German Center for Neurodegenerative Diseases (DZNE), Munich 81377, Germany		
Filler	Jule	jule.filler@med.uni-muenchen.de	Institute for Stroke and Dementia Research (ISD), University Hospital, LMU Munich, 81377 Munich, Germany			
Georgakis	Marios	marios.georgakis@med.uni-muenchen.de	Institute for Stroke and Dementia Research (ISD), University Hospital, LMU Munich, 81377 Munich, Germany			
Waegemann	Karin	karin.waegemann@med.uni-muenchen.de	Institute for Stroke and Dementia Research (ISD), University Hospital, LMU Munich, 81377 Munich, Germany	German Center for Neurodegenerative Diseases (DZNE), Munich 81377, Germany		

Janowitz	Daniel	daniel.janowitz@med.uni-muenchen.de	Institute for Stroke and Dementia Research (ISD), University Hospital, LMU Munich, 81377 Munich, Germany			
Fang	Rong	rong.fang@med.uni-muenchen.de	Institute for Stroke and Dementia Research (ISD), University Hospital, LMU Munich, 81377 Munich, Germany			
Dewenter	Anna	anna.dewenter@med.uni-muenchen.de	Institute for Stroke and Dementia Research (ISD), University Hospital, LMU Munich, 81377 Munich, Germany			
Kopczak	Anna	anna.kopczak@med.uni-muenchen.de	Institute for Stroke and Dementia Research (ISD), University Hospital, LMU Munich, 81377 Munich, Germany			
Wollenweber	Frank	frank.wollenweber@med.uni-muenchen.de	Institute for Stroke and Dementia Research (ISD), University Hospital, LMU Munich, 81377 Munich, Germany			
Wunderlich	Silke	silke.wunderlich@mri.tum.de	Department of Neurology, Klinikum rechts der Isar, School of Medicine, Technical University of Munich, Munich, Germany			
Ikenberg	Benno	benno.ikenberg@tum.de	Department of Neurology, Klinikum rechts der Isar, School of Medicine, Technical University of Munich, Munich, Germany			

Bernkopf	Kathleen	kathleen.bernkopf@mri.tum.de	Department of Neurology, Klinikum rechts der Isar, School of Medicine, Technical University of Munich, Munich, Germany			
Huber	Christiane	christiane.huber@mri.tum.de	Department of Neurology, Klinikum rechts der Isar, School of Medicine, Technical University of Munich, Munich, Germany			
Poppert	Holger	holger.poppert @helios-gesundheit.de	Neurology Department, Helios Klinikum München West, Munich, Germany			
Stöcker	Tony	tony.stoecker@dzne.de	German Center for Neurodegenerative Diseases (DZNE), Bonn 53127, Germany			
Düring	Marco	marco.duering@med.uni-muenchen.de	Institute for Stroke and Dementia Research (ISD), University Hospital, LMU Munich, 81377 Munich, Germany.	Medical Image Analysis Center (MIAC AG) and qbig, Department of Biomedical Engineering, University of Basel, Basel, Switzerland		
Spottke	Annika	annika.spottke@dzne.de	German Center for Neurodegenerative Diseases (DZNE), Bonn 53127, Germany			
Wagner	Michael	michael.wagner@dzne.de	German Center for Neurodegenerative Diseases (DZNE), Bonn 53127, Germany			
Neumann	Katja	Katja.Neumann @dzne.de	German Center for Neurodegenerative Diseases (DZNE), Magdeburg 39120, Germany			
Speck	Oliver	oliver.speck@ov gu.de	Department of Biomedical Magnetic Resonance, Institute for Physics, Otto-von-Guericke University Magdeburg, Magdeburg 39120, Germany	German Center for Neurodegenerative Diseases (DZNE), Magdeburg 39120, Germany	Leibniz Institute for Neurobiology, 39118 Magdeburg, Germany	Center for Behavioral Brain Sciences, 39106 Magdeburg, Germany

References

1. Adams HP, Jr., Bendixen BH, Kappelle LJ, et al. Classification of subtype of acute ischemic stroke. Definitions for use in a multicenter clinical trial. TOAST. Trial of Org 10172 in Acute Stroke Treatment. *Stroke* 1993; **24:** 35–41.
2. Alberti KGMM, Eckel RH, Grundy SM, et al. Harmonizing the Metabolic Syndrome. *Circulation* 2009; **120:** 1640–1645.
3. Georgakis MK, Fang R, Düring M, et al. Cerebral small vessel disease burden and cognitive and functional outcomes after stroke: A multicenter prospective cohort study. *Alzheimers Dement* 2023; **19:** 1152–1163.
4. Wardlaw JM, Smith EE, Biessels GJ, et al. Neuroimaging standards for research into small vessel disease and its contribution to ageing and neurodegeneration. *Lancet Neurol* 2013; **12:** 822–838.
5. Duering M, Biessels GJ, Brodtmann A, et al. Neuroimaging standards for research into small vessel disease-advances since 2013. *Lancet Neurol* 2023; **22:** 602–618.
6. Konieczny MJ, Dewenter A, Ter Telgte A, et al. Multi-shell Diffusion MRI Models for White Matter Characterization in Cerebral Small Vessel Disease. *Neurology* 2021; **96:** e698–e708.
7. Baykara E, Gesierich B, Adam R, et al. A Novel Imaging Marker for Small Vessel Disease Based on Skeletonization of White Matter Tracts and Diffusion Histograms. *Ann Neurol* 2016; **80:** 581–592.
8. Berres M, Monsch AU, Bernasconi F, Thalmann B, Stähelin HB. Normal ranges of neuropsychological tests for the diagnosis of Alzheimer's disease. *Stud Health Technol Inform* 2000; **77:** 195–199.
9. Bäumler G. Farbe-Wort-Interferenztest (FWIT) nach JR Stroop: Verlag für Psychologie Dr. CJ Hogrefe; 1985.
10. Somerville J, Tremont G, Stern RA. The Boston Qualitative Scoring System as a measure of executive functioning in Rey-Osterrieth Complex Figure performance. *J Clin Exp Neuropsychol* 2000; **22:** 613–621.
11. Young JC, Sawyer RJ, Roper BL, Baughman BC. Expansion and re-examination of Digit Span effort indices on the WAIS-IV. *Clin Neuropsychol* 2012; **26:** 147–159.
12. URL: https://www.memoryclinic.ch/de/main-navigation/neuropsychologen/cerad-plus/auswertungprogramm/cerad-plus-online/. (last accessed: 05.03.2025)
13. Tremblay MP, Potvin O, Callahan BL, et al. Normative data for the Rey-Osterrieth and the Taylor complex figure tests in Quebec-French people. *Arch Clin Neuropsychol* 2015; **30:** 78–87.
14. Van der Elst W, Van Boxtel MP, Van Breukelen GJ, Jolles J. The Stroop color-word test: influence of age, sex, and education; and normative data for a large sample across the adult age range. *Assessment* 2006; **13:** 62–79.
15. Wechsler D. Wechsler adult intelligence scale. *Front Psychol* 1997.
16. Morris JC. The Clinical Dementia Rating (CDR): current version and scoring rules. *Neurology* 1993; **43:** 2412–2414.
17. Zietemann V, Kopczak A, Müller C, Wollenweber FA, Dichgans M. Validation of the Telephone Interview of Cognitive Status and Telephone Montreal Cognitive Assessment Against Detailed Cognitive Testing and Clinical Diagnosis of Mild Cognitive Impairment After Stroke. *Stroke* 2017; **48:** 2952–2957.
18. Pendlebury ST, Rothwell PM. Incidence and prevalence of dementia associated with transient ischaemic attack and stroke: analysis of the population-based Oxford Vascular Study. *Lancet Neurol* 2019; **18:** 248–258.
19. Wollenweber FA, Zietemann V, Rominger A, et al. The Determinants of Dementia After Stroke (DEDEMAS) Study: protocol and pilot data. *Int J Stroke* 2014; **9:** 387–392.
20. Pendlebury ST, Rothwell PM. Prevalence, incidence, and factors associated with pre-stroke and post-stroke dementia: a systematic review and meta-analysis. *Lancet Neurol* 2009; **8:** 1006–1018.
21. Hothorn T. maxstat: Maximally Selected Rank Statistics 2017. Available from: https://CRAN.R-project.org/package=maxstat. (last accessed: 05.03.2025)
22. Mok VCT, Lam BYK, Wang Z, et al. Delayed-onset dementia after stroke or transient ischemic attack. *Alzheimers Dement* 2016; **12:** 1167–1176.
23. Ismail M, Mok VC, Wong A, et al. Risk factors for delayed-onset dementia after stroke or transient ischemic attack-A five-year longitudinal cohort study. *Int J Stroke* 2022; **17:** 517–525.
24. Fang R, Duering M, Bode FJ, et al. Risk factors and clinical significance of post-stroke incident ischemic lesions. *Alzheimers Dement* 2024; **20:** 8412–8428.
25. Therneau TM. A Package for Survival Analysis in R 2024. Available from: https://CRAN.R-project.org/package=survival. (last accessed: 05.03.2025)
26. Fine JP. Regression modeling of competing crude failure probabilities. *Biostatistics*. 2001;**2:**85–97.
27. Austin PC, Lee DS, Fine JP. Introduction to the Analysis of Survival Data in the Presence of Competing Risks. *Circulation* 2016; **133:** 601–609.
28. Royston P, Parmar MK. Flexible parametric proportional-hazards and proportional-odds models for censored survival data, with application to prognostic modelling and estimation of treatment effects. *Stat Med* 2002; **21:** 2175–2197.

29. Pendlebury ST, Poole D, Burgess A, Duerden J, Rothwell PM. APOE-ε4 Genotype and Dementia Before and After Transient Ischemic Attack and Stroke: Population-Based Cohort Study. *Stroke* 2020; **51:** 751–758.
30. Filler J, Georgakis MK, Dichgans M. Risk factors for cognitive impairment and dementia after stroke: a systematic review and meta-analysis. *Lancet Healthy Longev* 2024; **5:** e31–e44.
31. Jokinen H, Koikkalainen J, Laakso HM, et al. Global Burden of Small Vessel Disease-Related Brain Changes on MRI Predicts Cognitive and Functional Decline. *Stroke* 2020; **51:** 170–178.
32. Mok VCT, Lam BYK, Wong A, Ko H, Markus HS, Wong LKS. Early-onset and delayed-onset poststroke dementia — revisiting the mechanisms. *Nat Rev Neurol* 2017; **13:** 148–159.
33. Qureshi D, Collister J, Allen NE, Kuźma E, Littlejohns T. Association between metabolic syndrome and risk of incident dementia in UK Biobank. *Alzheimers Dement* 2024; **20:** 447–458.
34. Machado-Fragua MD, Fayosse A, Yerramalla MS, et al. Association of Metabolic Syndrome With Incident Dementia: Role of Number and Age at Measurement of Components in a 28-Year Follow-up of the Whitehall II Cohort Study. *Diabetes Care* 2022; **45:** 2127–2135.
