# Supplementary material for: Risk factors for dementia and cognitive impairment within 5 years after stroke: a prospective multicentre cohort study
Source: Lancet Reg Health Eur. 2025 Aug 19;56:101428. doi: 10.1016/j.lanepe.2025.101428 (PMC12396445; doi:10.1016/j.lanepe.2025.101428)
Supplement: DEMDAS Investigators [file mmc2.pdf]

**First Name(s)**	**Last Name**
Tatjana	Wittenberg
Jan F	Scheitz
Harald	Prüss
Pia Sophie	Sperber
Alexander H	Nave
Anna	Kufner Ibaroule
Julius N	Meißner
Taraneh	Ebrahimi
Julia	Nordsiek
Niklas	Beckonert
Matthias	Schmitz
Stefan	Goebel
Timothy	Bunck
Julia	Schütte-Schmidt
Sabine	Nuhn
Corinna	Volpers
Peter	Dechent
Matthias	Bähr
Anna	Kopczak
Frank	Wollenweber
Christiane	Huber
Holger	Poppert
Tony	Stöcker
Katja	Neumann
Oliver	Speck
