# Supplementary material for: Risk factors for dementia and cognitive impairment within 5 years after stroke: a prospective multicentre cohort study
Source: Lancet Reg Health Eur. 2025 Aug 19;56:101428. doi: 10.1016/j.lanepe.2025.101428 (PMC12396445; doi:10.1016/j.lanepe.2025.101428)
Supplement: German Abstract [file mmc3.pdf]

This translation in German was submitted by the authors and we reproduce it as supplied. It has not been peer reviewed. Our editorial processes have only been applied to the original abstract in English, which should serve as reference for this manuscript.

Risikofaktoren für Demenz und kognitive Störungen innerhalb von 5 Jahren nach Schlaganfall: eine prospektive multizentrische Kohortenstudie

Zusammenfassung

Hintergrund: Kognitive Störungen gehören zu den häufigsten Langzeitfolgen eines Schlaganfalls. Ziel dieser Studie war es, Risikofaktoren für Demenz und kognitive Störungen innerhalb von fünf Jahren nach Schlaganfall zu identifizieren.

Methoden: Die DEMDAS ("Deutsches Zentrum für Neurodegenerative Erkrankungen (DZNE) mechanisms of dementia after stroke") Studie ist eine prospektive Kohortenstudie von Schlaganfallpatient*innen, die zwischen dem 1. Mai 2011 und dem 31. Januar 2019 in eines von sechs tertiären Schlaganfallzentren in Deutschland eingewiesen wurden. Eingeschlossen wurden Patient*innen mit ischämischem oder hämorrhagischem Schlaganfall ohne vorbestehende Demenz. Sie erhielten eine Baseline-Untersuchung sowie regelmäßige klinische, neuropsychologische und bildgebende Follow-up-Untersuchungen über bis zu fünf Jahre. Die letzten Follow-ups wurden im Januar 2024 abgeschlossen. Der primäre Endpunkt war das Auftreten einer Demenz, bestimmt anhand ausführlicher kognitiver Testungen, Befragungen der Patient*innen und Angehörigen sowie Sichtung aller medizinischen Unterlagen. Sekundäre Endpunkte waren i) früh einsetzende Demenz (3-6 Monate nach Schlaganfall), ii) später einsetzende Demenz (>6 Monate) und iii) jegliche kognitive Störung (leichte kognitive Störung enschließlich Demenz). Assoziationen zwischen Baseline-Risikofaktoren und Demenz wurden mit Cox-Regressionsmodellen untersucht, adjustiert für Alter, Geschlecht, Bildungsgrad und Schlaganfallschwere.

Ergebnisse: Von 736 eingeschlossenen Patient*innen (33 % weiblich; mittleres Alter 68,0 Jahre [SD 11,2]; medianer National Institutes of Health Stroke Scale (NIHSS) Score bei Aufnahme 3 [IQR 1–5]) konnten 557 (76 %) bis zum Tod oder Studienende nachverfolgt werden. 706 (96 %) hatten mindestens ein Follow-up und gingen in die Demenzanalyse ein. Über einen medianen Follow-up-Zeitraum von 5,0 Jahren (IQR 3,3–5,1) wurden 55 neue Demenzfälle diagnostiziert (6-Monats-Inzidenz: 3,1 % [1,8–4,5]; 5-Jahres-Inzidenz: 8,8 % [6,5–11,1]); davon 21 (38 %) zwischen 3 und 6 Monaten nach dem Schlaganfall. Ein erhöhtes 5-Jahres-Demenzrisiko war assoziiert mit höherem Alter (HR 1,13 [95 %-KI 1,08–1,18] pro Jahr), größerer Schlaganfallschwere (1,08 [1,03–1,13] pro NIHSS-Punkt), geringerer Bildung (1,16 [1,05–1,28] pro Jahr weniger), kognitiver Beeinträchtigung in der Akutphase (5,86 [2,21–15,58]), niedrigerem Barthel-Index (1,10 [1,05-1,16] pro 5 Punkte weniger), Vorhofflimmern

(1,91 [1,10–3,30]), metabolischem Syndrom (2,05 [1,15–3,64]) – insbesondere niedrigem HDL-Cholesterin (2,61 [1,50–4,52]) und Prä-/Diabetes mellitus (2,13 [1,13–4,00]) – Bildgebungsmarkern für Small Vessel Disease sowie mit erneuten Schlaganfällen während des Follow-ups (2,36 [1,16–4,83]). Patientinnen, die eine akute Reperfusionstherapie erhielten, hatten ein um 65 % niedrigeres Demenzrisiko als solche ohne (0,35 [0,16–0,77]). Während Faktoren der Akutschwere des Schlaganfalls vor allem mit früh einsetzender Demenz assoziiert waren, war das metabolische Syndrom ein starker Risikofaktor für später einsetzende Demenz. Der Zusammenhang zwischen metabolischem Syndrom und Demenz blieb unabhängig von erneuten Schlaganfällen und über Altersgruppen hinweg bestehen: Die kumulative 5-Jahres-Inzidenz reichte von 1,7 % (0,0–4,0) bei ≤65-Jährigen ohne metabolisches Syndrom bis 24,5 % (14,3–33,4) bei ≥74-Jährigen mit metabolischem Syndrom.

Interpretation: Das Demenzrisiko nach Schlaganfall ist multifaktoriell, und die Risikoprofile für früh und später einsetzende Demenz unterscheiden sich. Das metabolische Syndrom und dessen Komponente niedriges HDL-Cholesterin stellen neu identifizierte Risikofaktoren und potenzielle Ziele für die Prävention kognitiver Verschlechterung und Demenz nach Schlaganfall dar.

Finanzierung: Deutsches Zentrum für Neurodegenerative Erkrankungen (DZNE)
